# Supplementary figures and images for: EDIL3 is a potential prognostic biomarker that correlates with immune infiltrates in gastric cancer (part 1 of 2)
Source: PeerJ. 2023 Aug 9;11:e15559. doi: 10.7717/peerj.15559 (PMC10422953; doi:10.7717/peerj.15559)

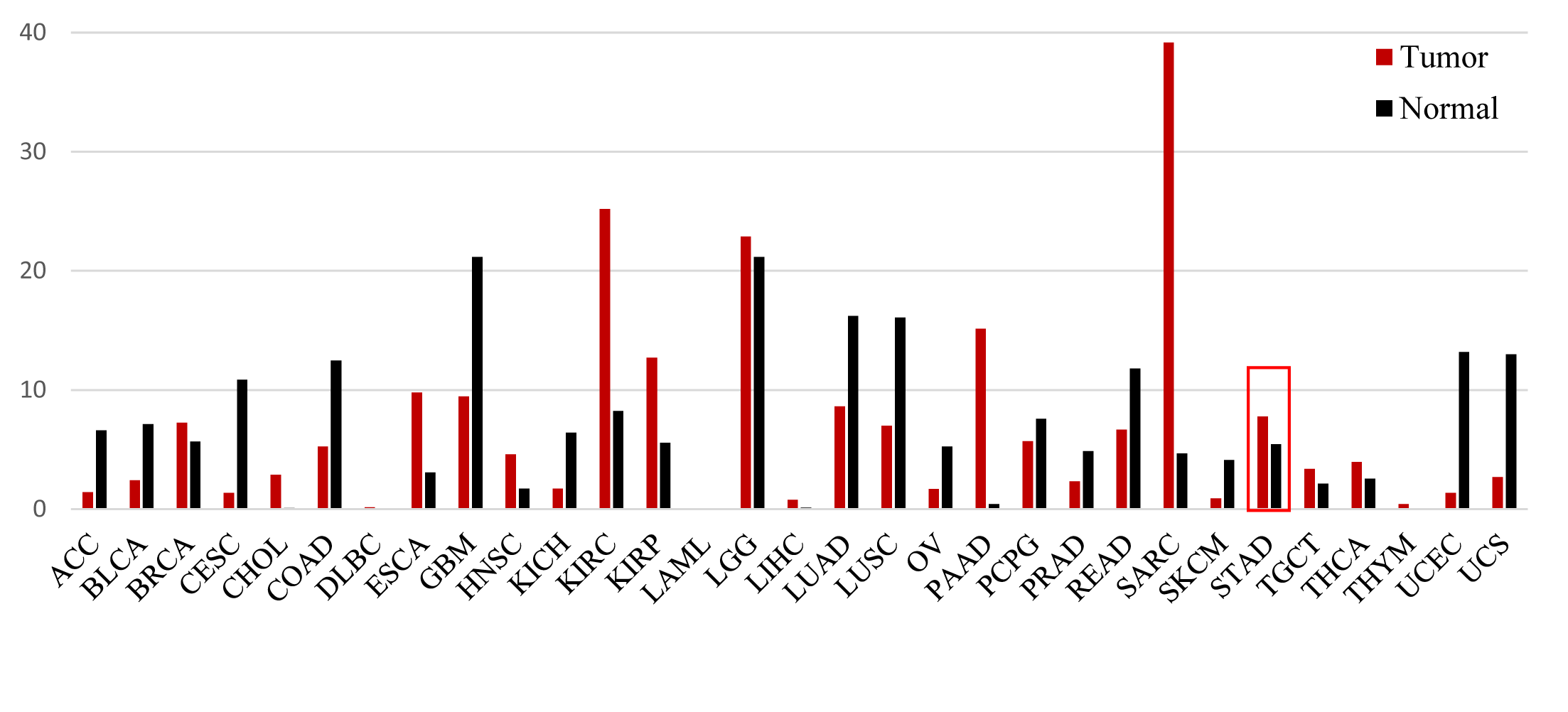

Supplement: Data S1 [file peerj-11-15559-s003.zip › Raw data 1/Raw figure 1-3/Figure 1/Fig 1A.tif]

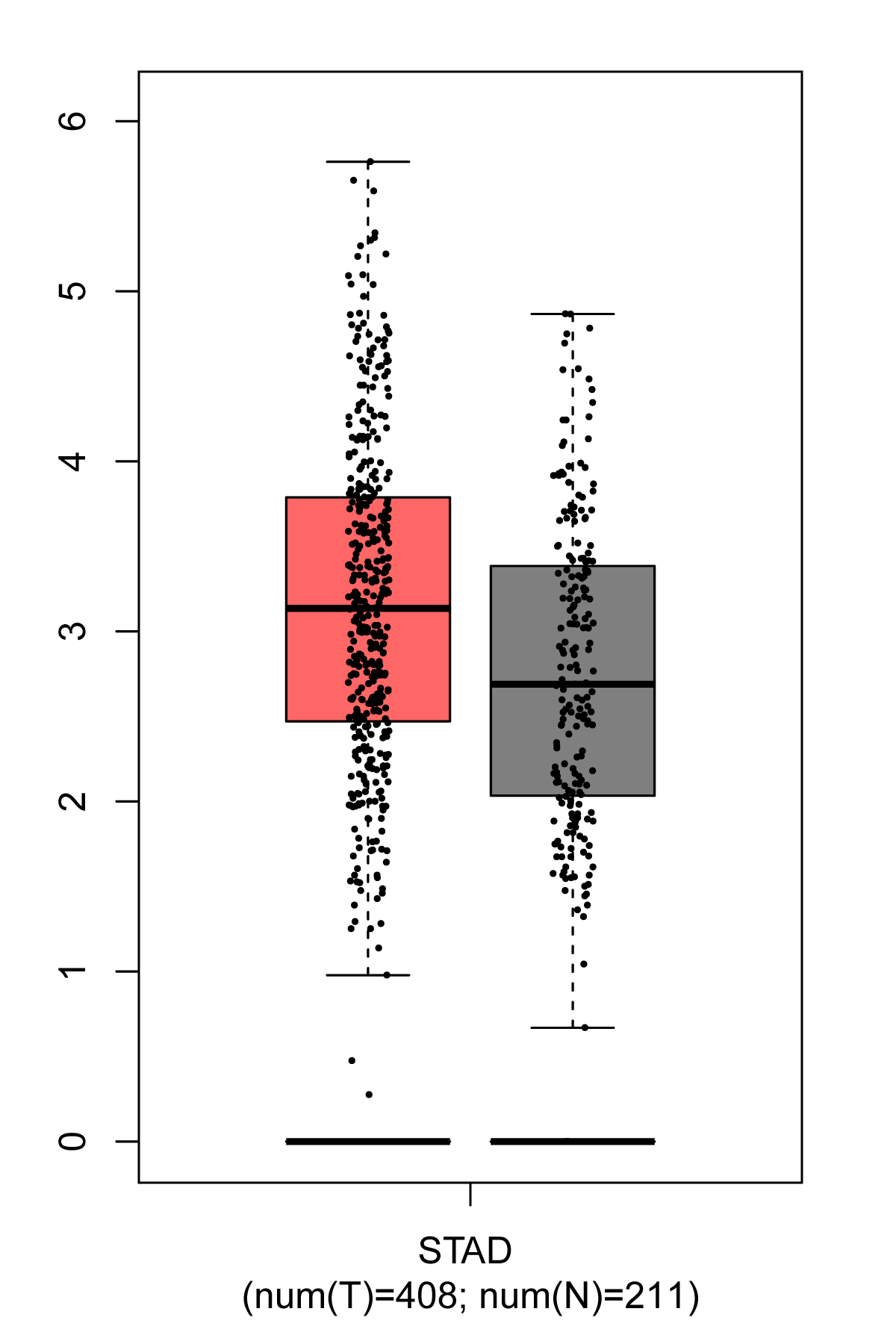

Supplement: Data S1 [file peerj-11-15559-s003.zip › Raw data 1/Raw figure 1-3/Figure 1/Fig 1B.tif]

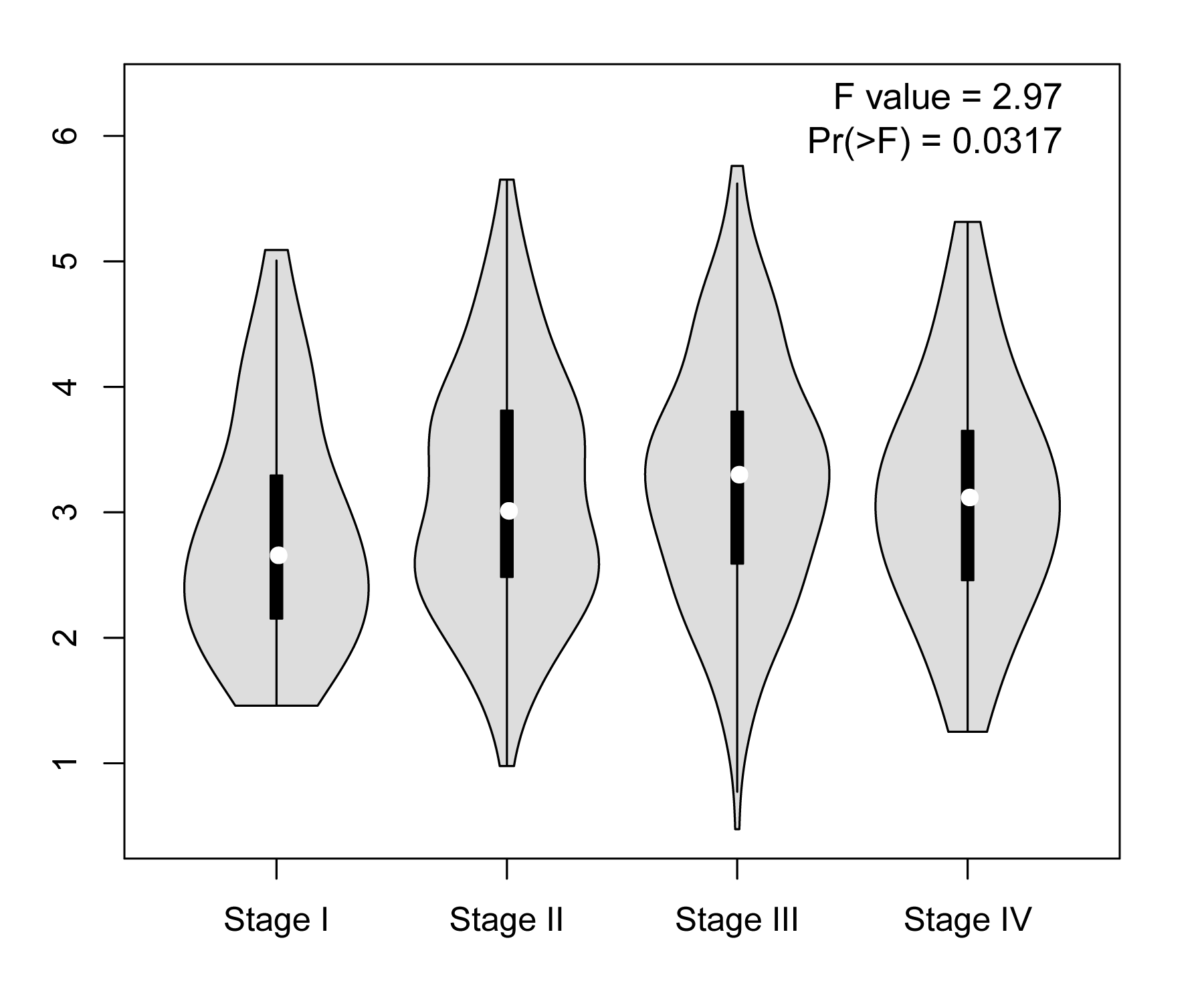

Supplement: Data S1 [file peerj-11-15559-s003.zip › Raw data 1/Raw figure 1-3/Figure 1/Fig 1C.tif]

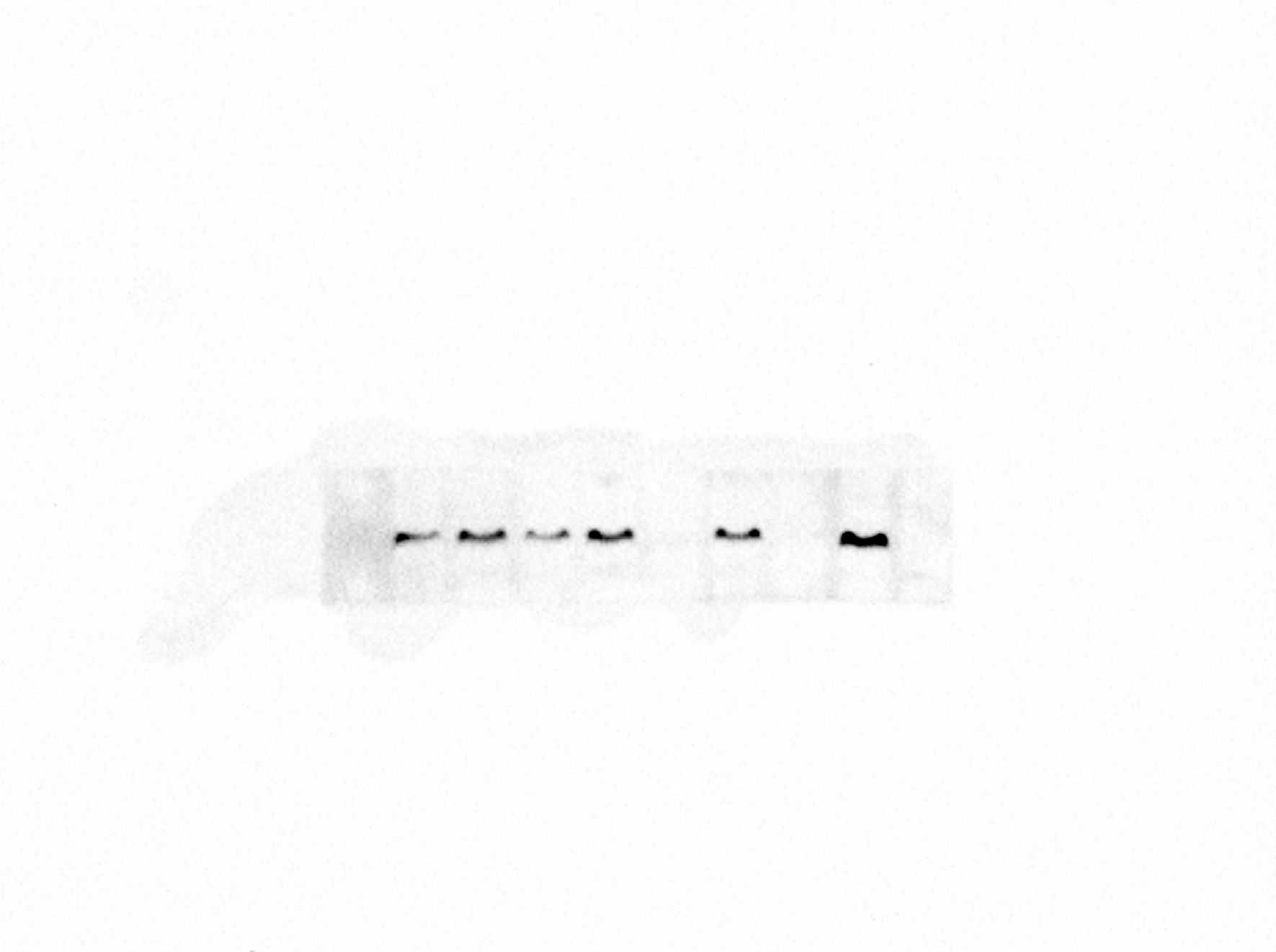

Supplement: Data S1 [file peerj-11-15559-s003.zip › Raw data 1/Raw figure 1-3/Figure 1/Fig 1F EDIL3.tif]

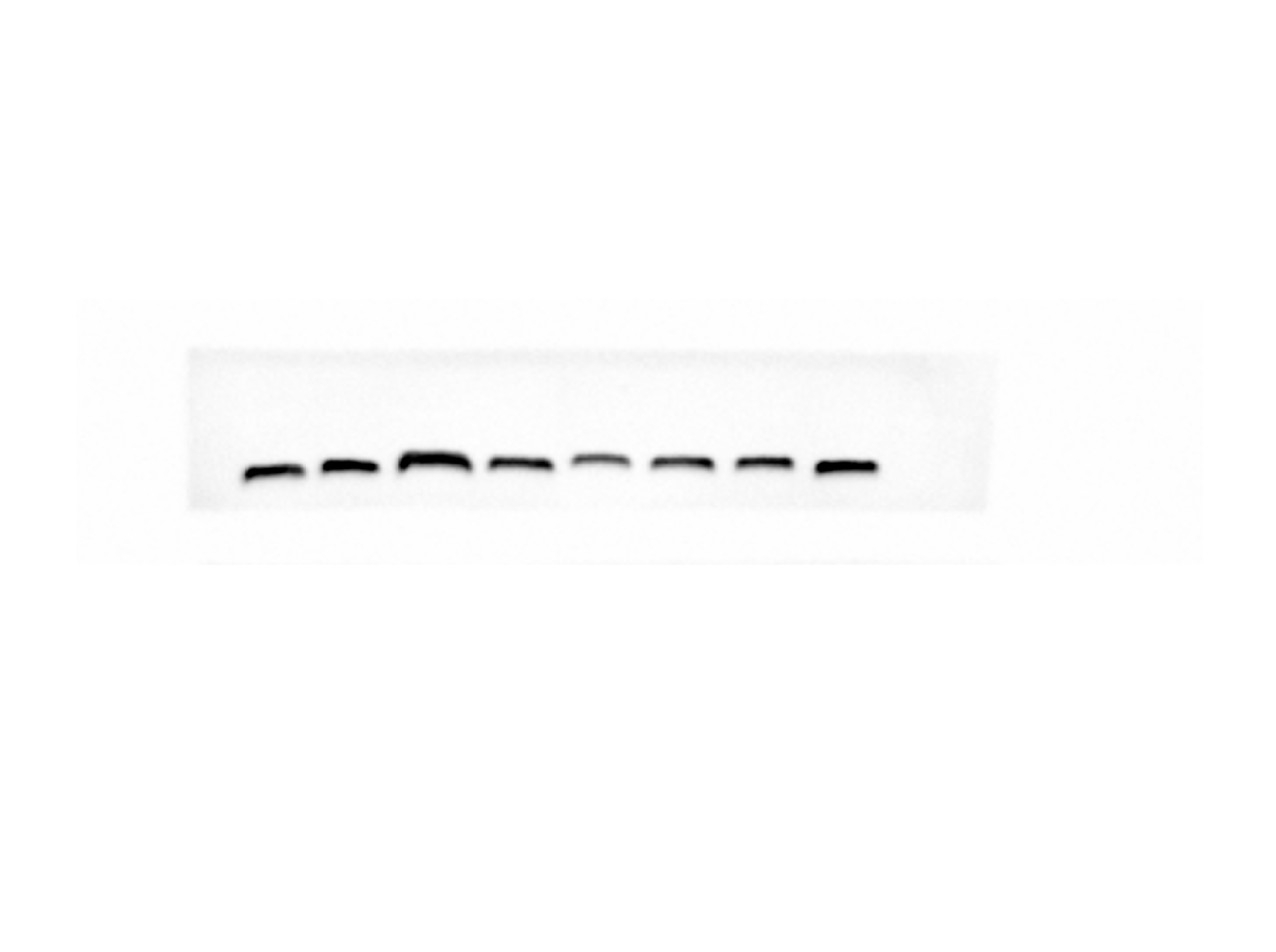

Supplement: Data S1 [file peerj-11-15559-s003.zip › Raw data 1/Raw figure 1-3/Figure 1/Fig 1F GAPDH.tif]

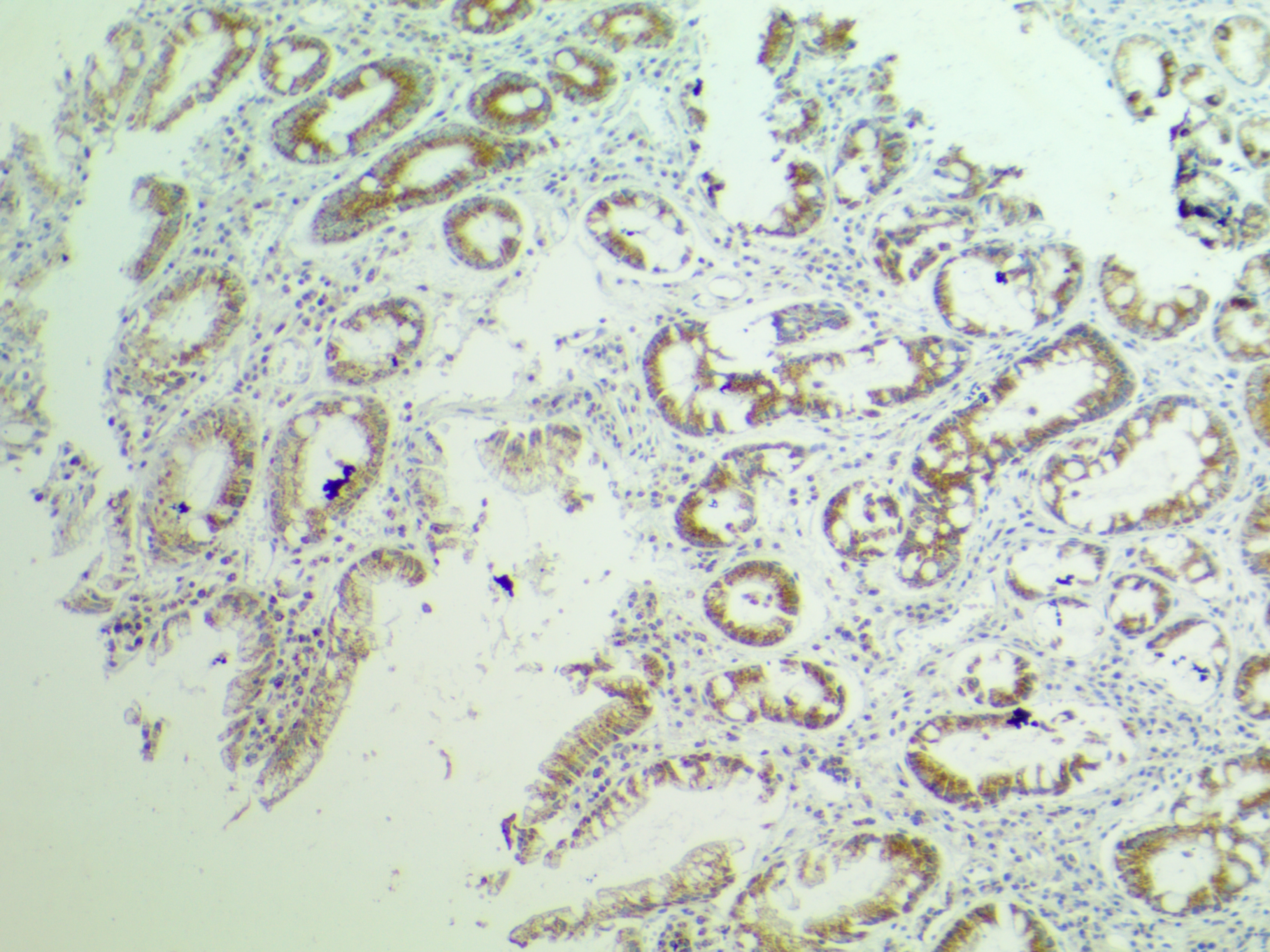

Supplement: Data S1 [file peerj-11-15559-s003.zip › Raw data 1/Raw figure 1-3/Figure 1/Fig 1G 100.jpg]

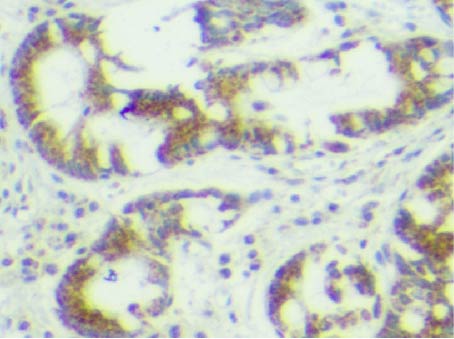

Supplement: Data S1 [file peerj-11-15559-s003.zip › Raw data 1/Raw figure 1-3/Figure 1/Fig 1G 400.jpg]

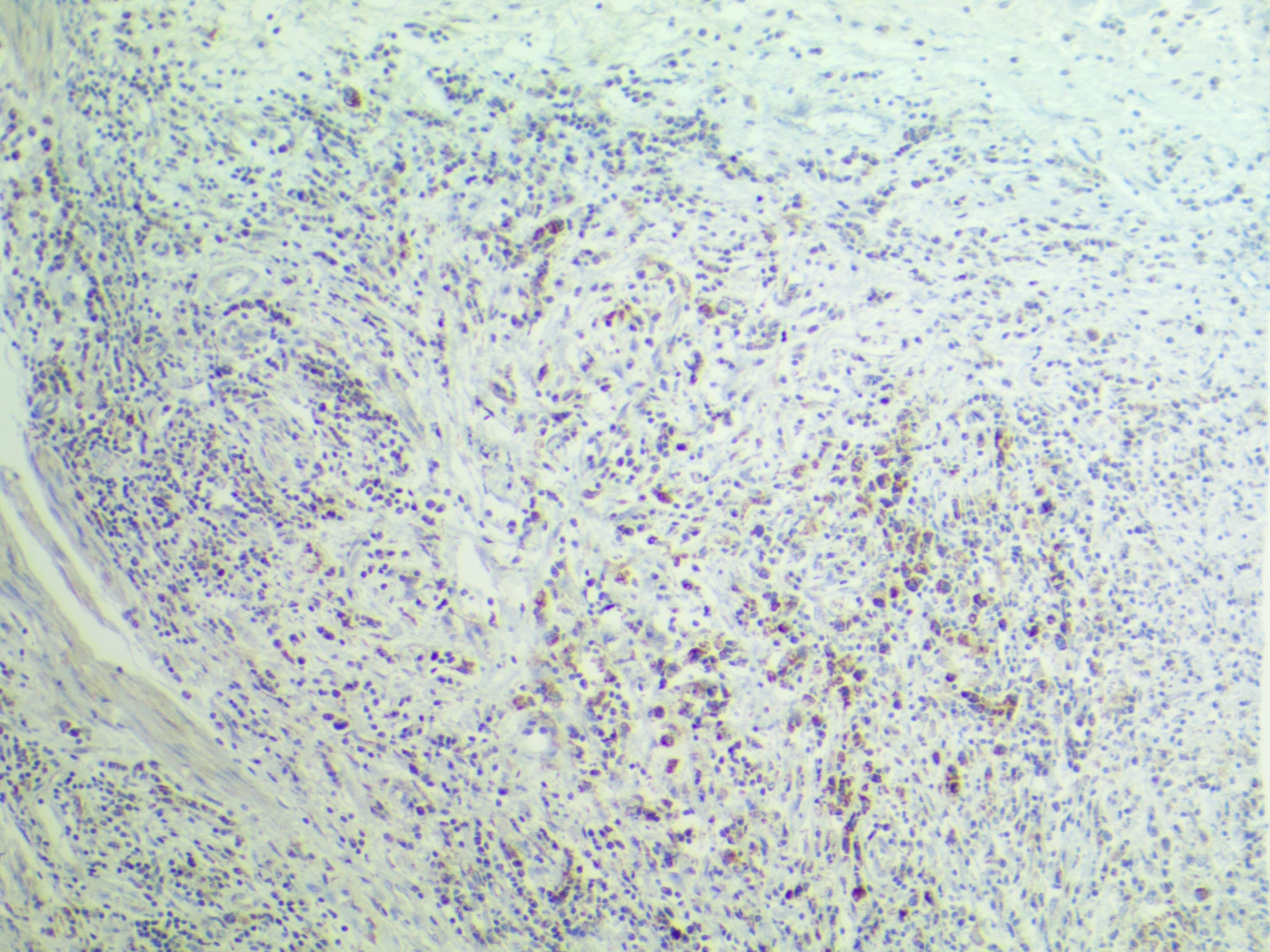

Supplement: Data S1 [file peerj-11-15559-s003.zip › Raw data 1/Raw figure 1-3/Figure 1/Fig 1H 100.jpg]

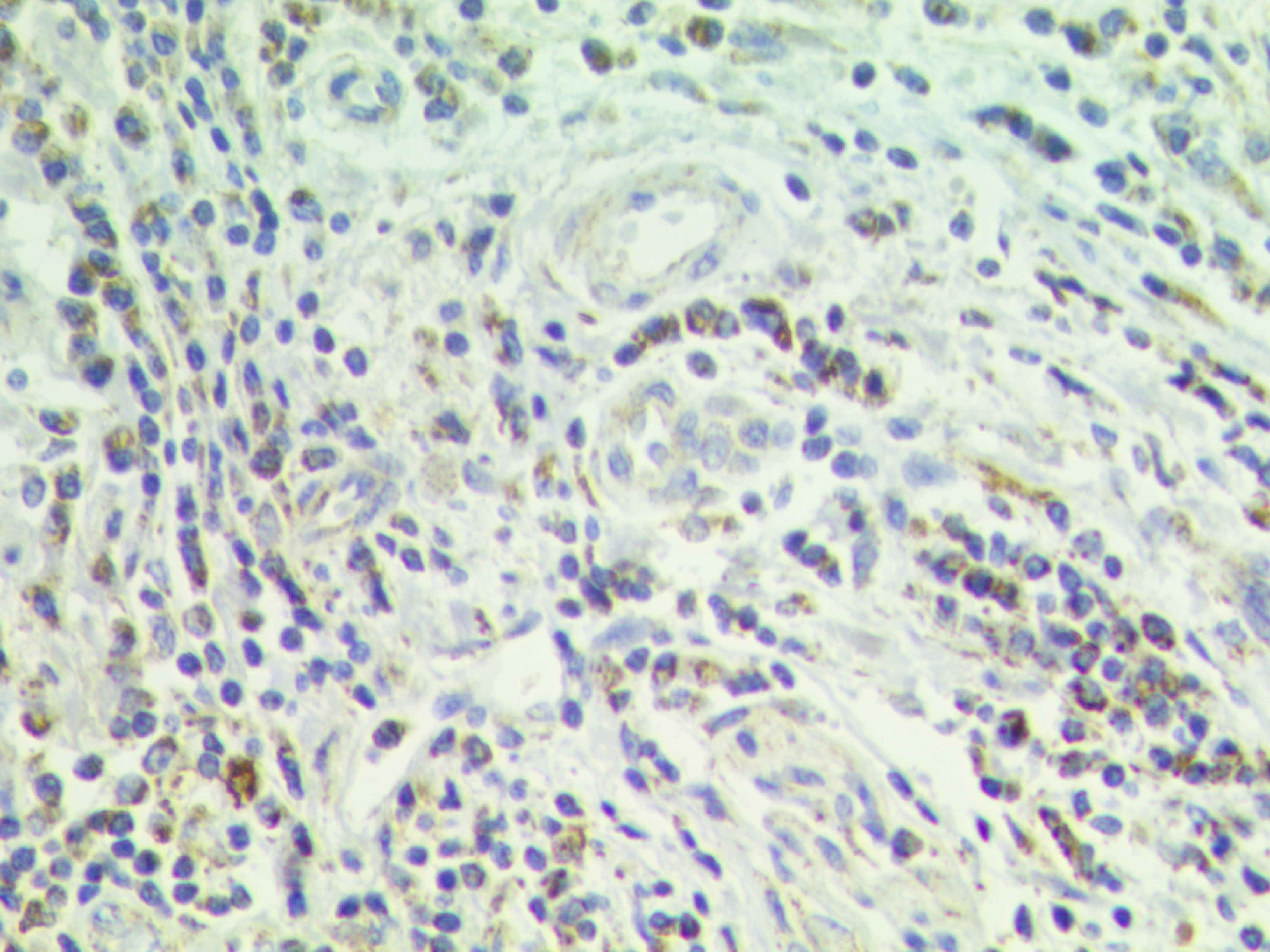

Supplement: Data S1 [file peerj-11-15559-s003.zip › Raw data 1/Raw figure 1-3/Figure 1/Fig 1H 400.jpg]

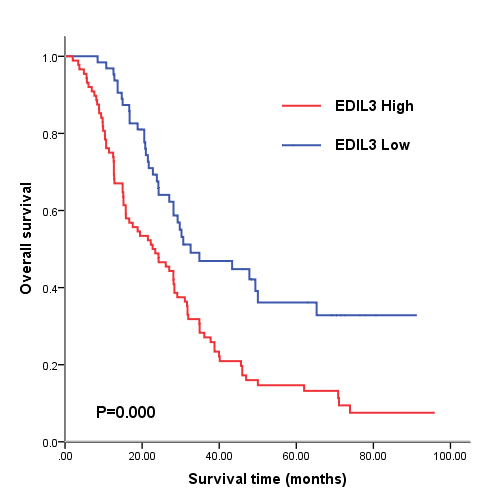

Supplement: Data S1 [file peerj-11-15559-s003.zip › Raw data 1/Raw figure 1-3/Figure 2/Fig 2A EDIL3.tif]

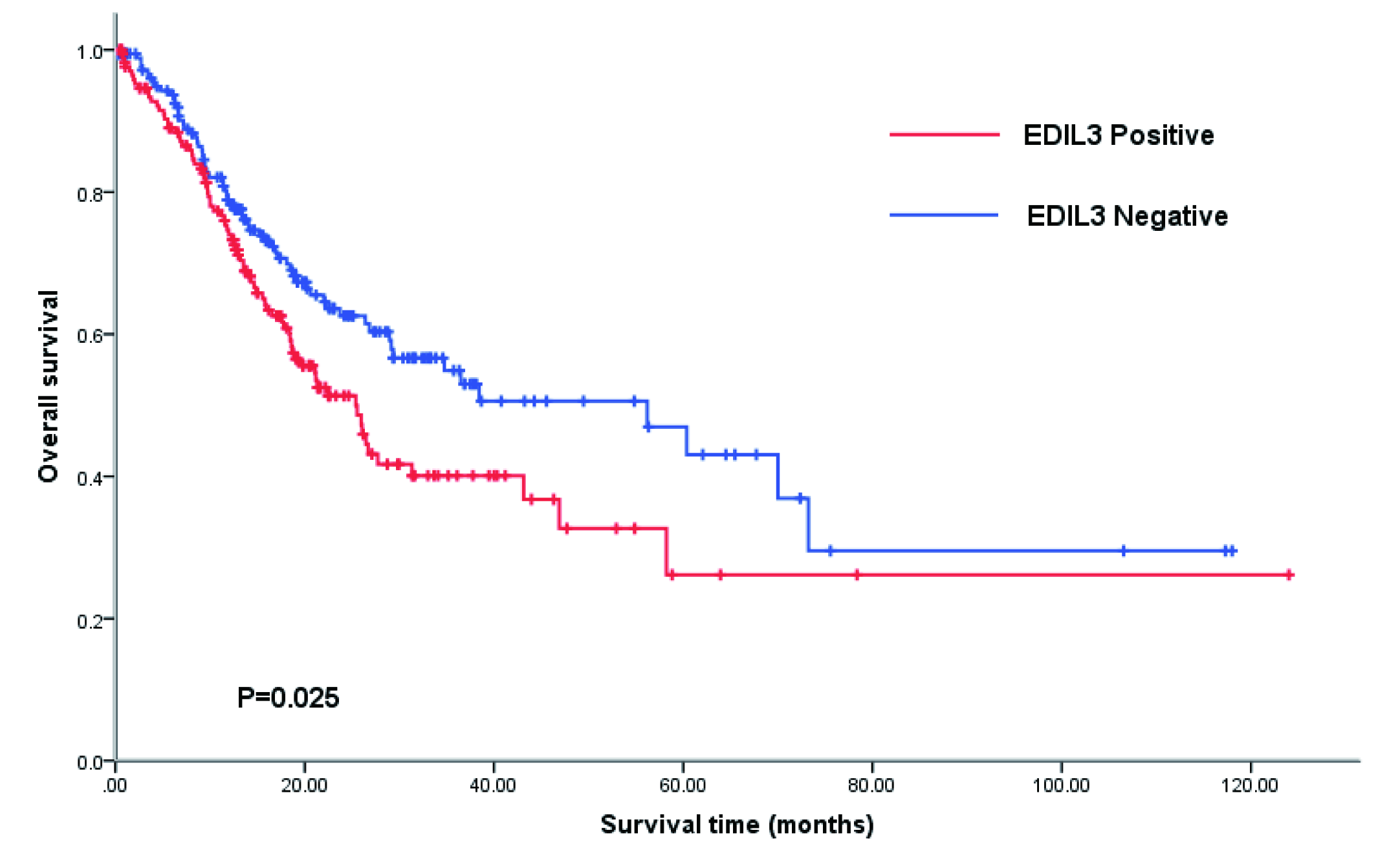

Supplement: Data S1 [file peerj-11-15559-s003.zip › Raw data 1/Raw figure 1-3/Figure 2/Fig 2B HPA.tif]

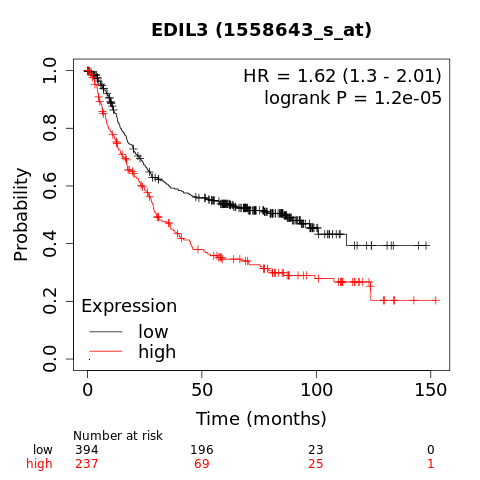

Supplement: Data S1 [file peerj-11-15559-s003.zip › Raw data 1/Raw figure 1-3/Figure 2/Fig 2C km.png]

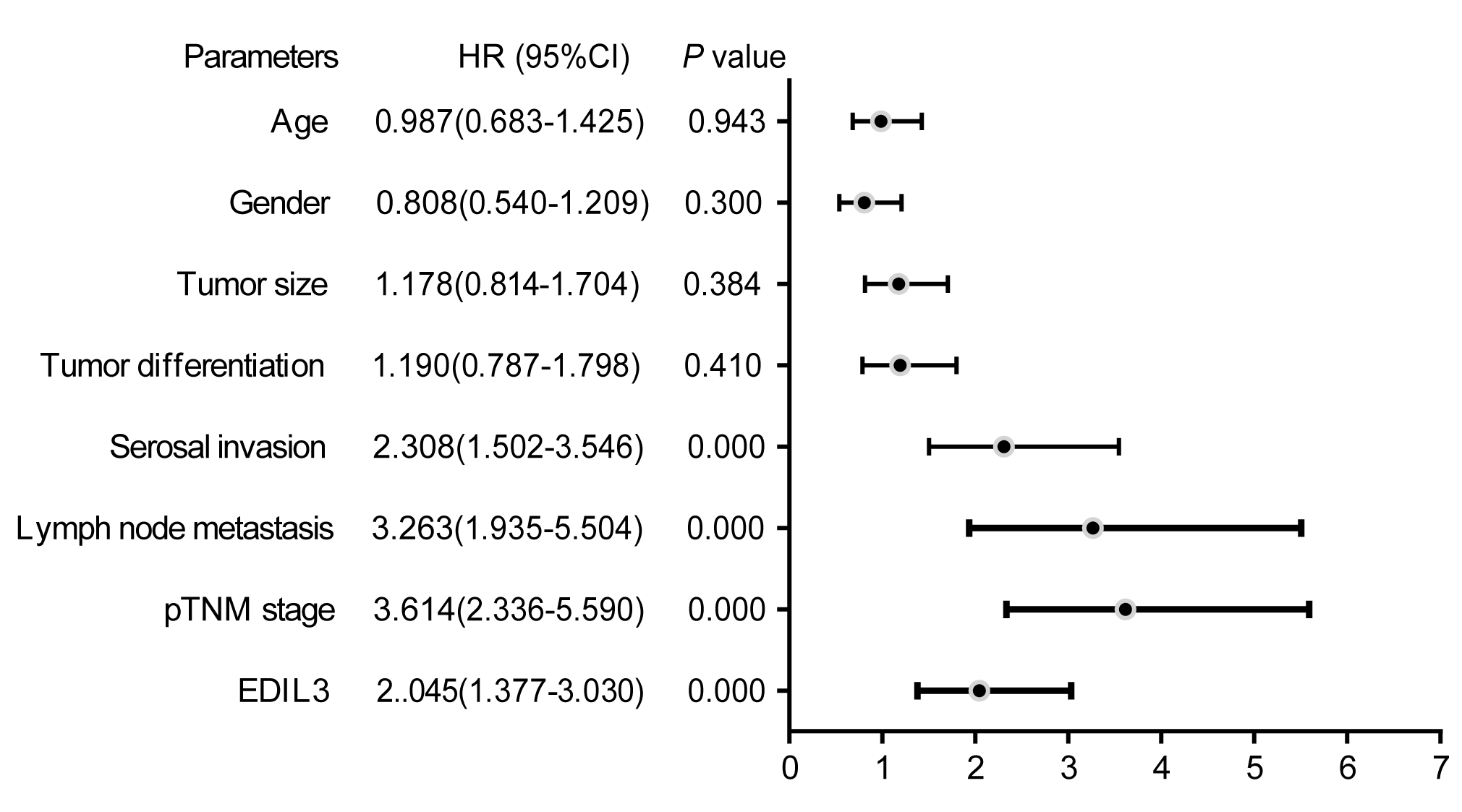

Supplement: Data S1 [file peerj-11-15559-s003.zip › Raw data 1/Raw figure 1-3/Figure 2/Fig 2D.tif]

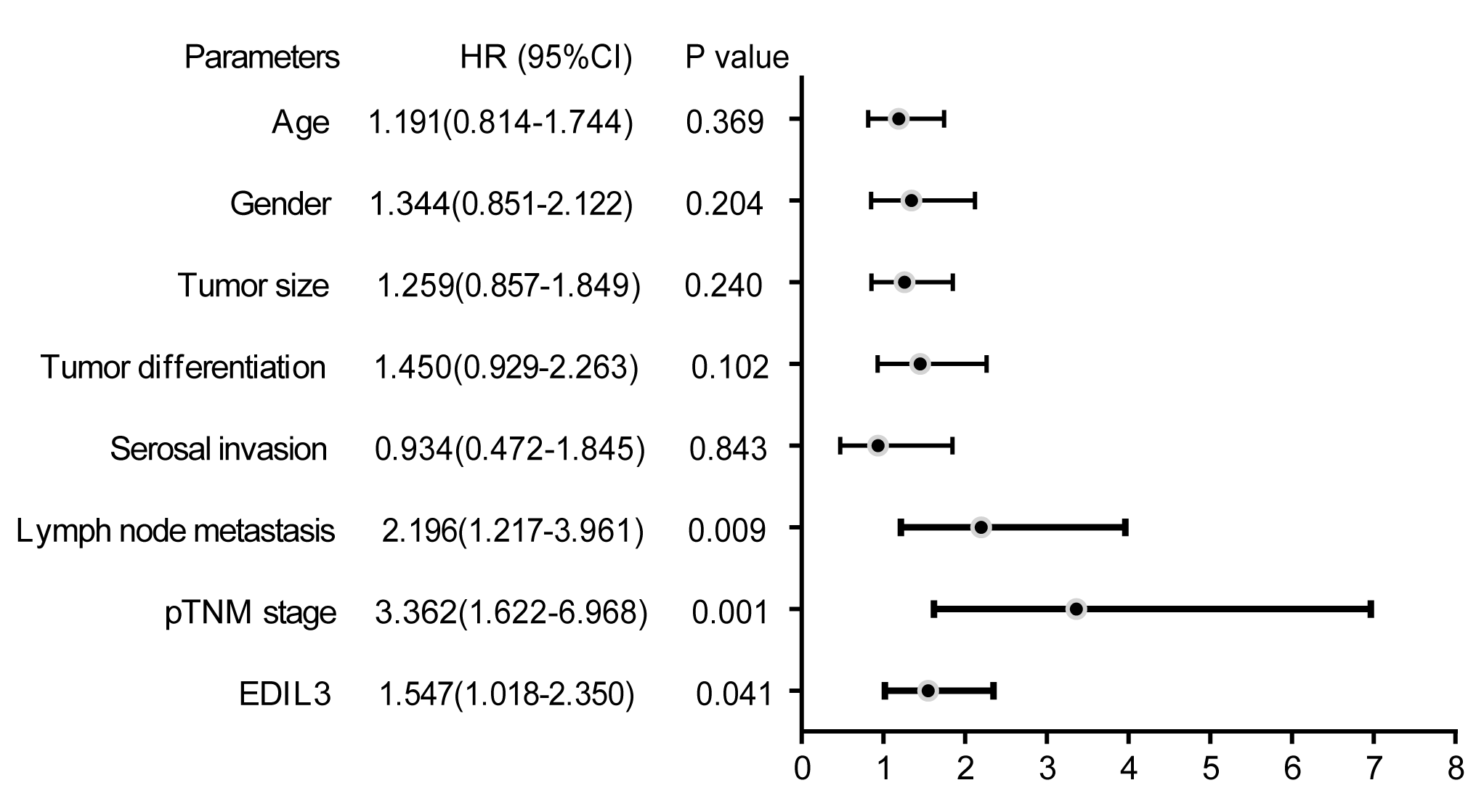

Supplement: Data S1 [file peerj-11-15559-s003.zip › Raw data 1/Raw figure 1-3/Figure 2/Fig 2E.tif]

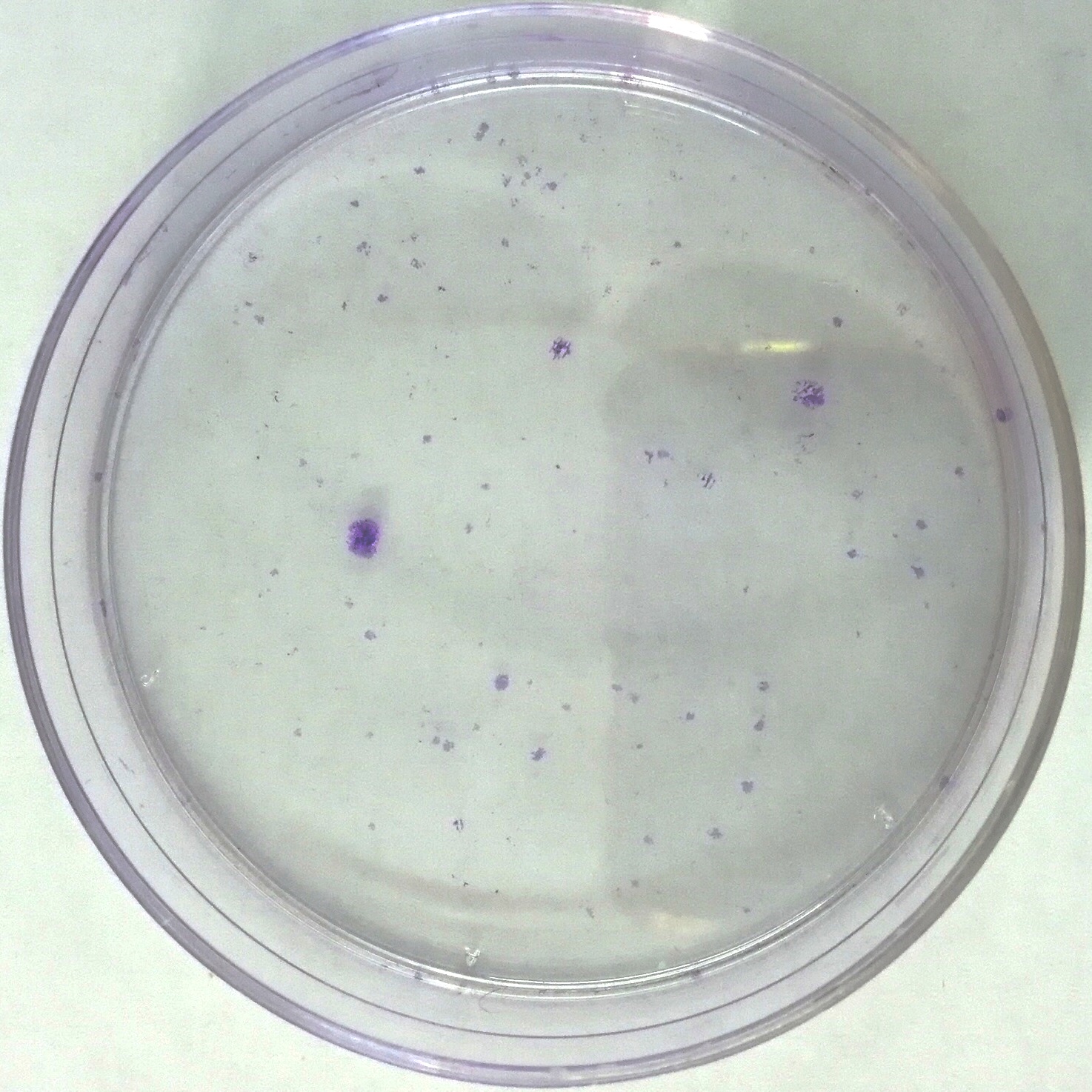

Supplement: Data S1 [file peerj-11-15559-s003.zip › Raw data 1/Raw figure 1-3/Figure 3/Fig 3C 823 sh-EDIL3.jpg]

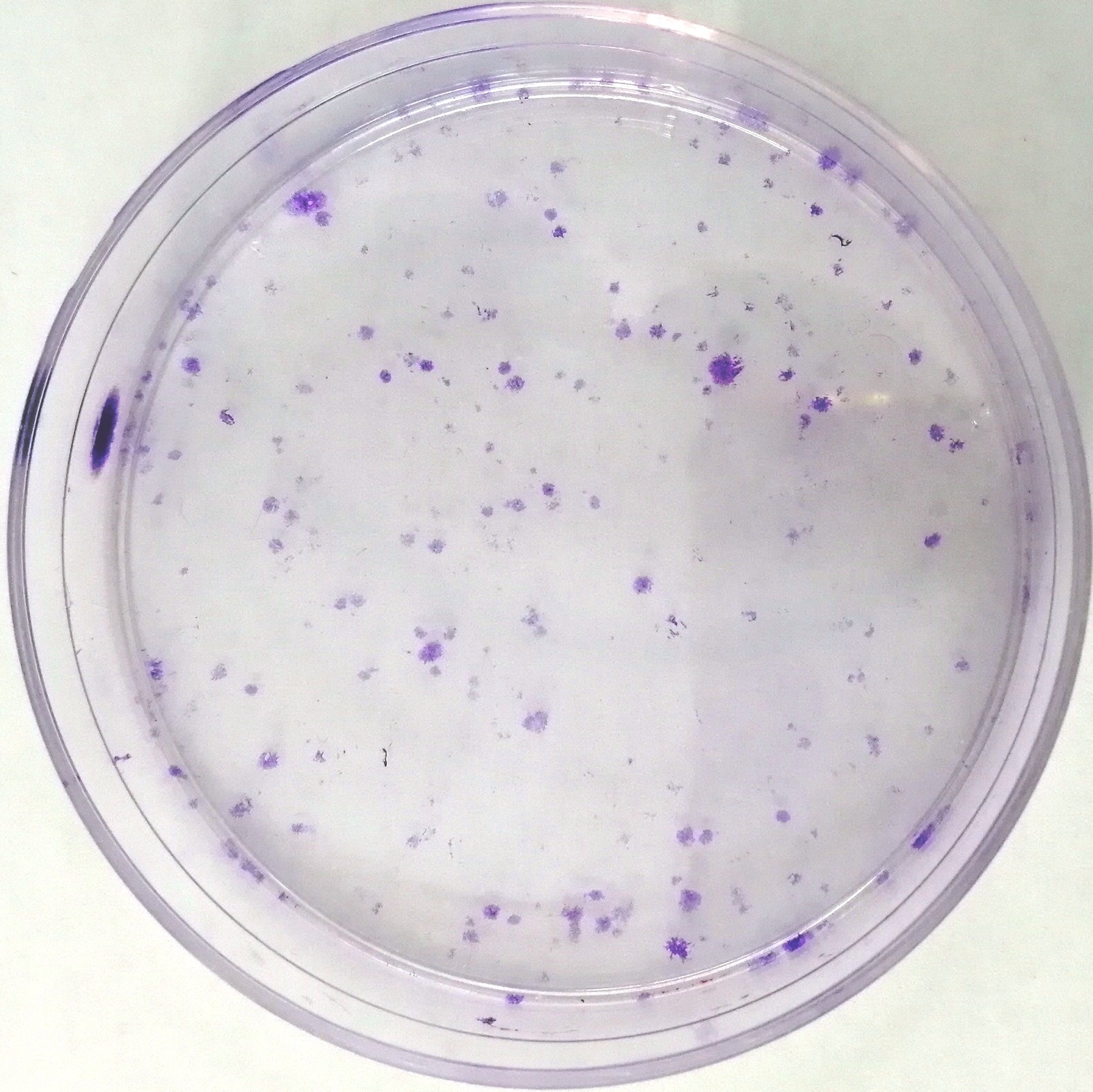

Supplement: Data S1 [file peerj-11-15559-s003.zip › Raw data 1/Raw figure 1-3/Figure 3/Fig 3C 823 sh-NA.jpg]

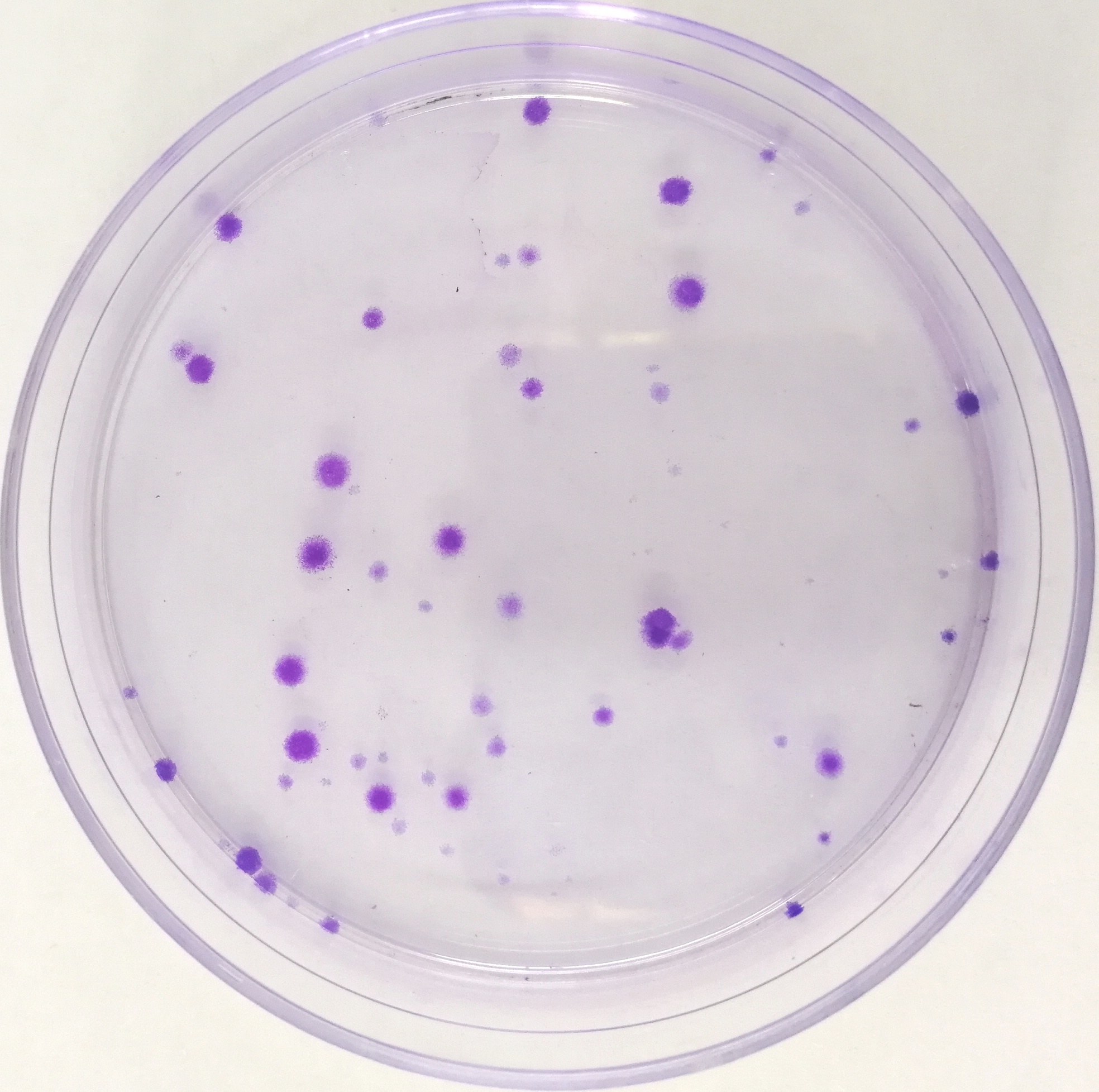

Supplement: Data S1 [file peerj-11-15559-s003.zip › Raw data 1/Raw figure 1-3/Figure 3/Fig 3C AGS sh-EDIL3.jpg]

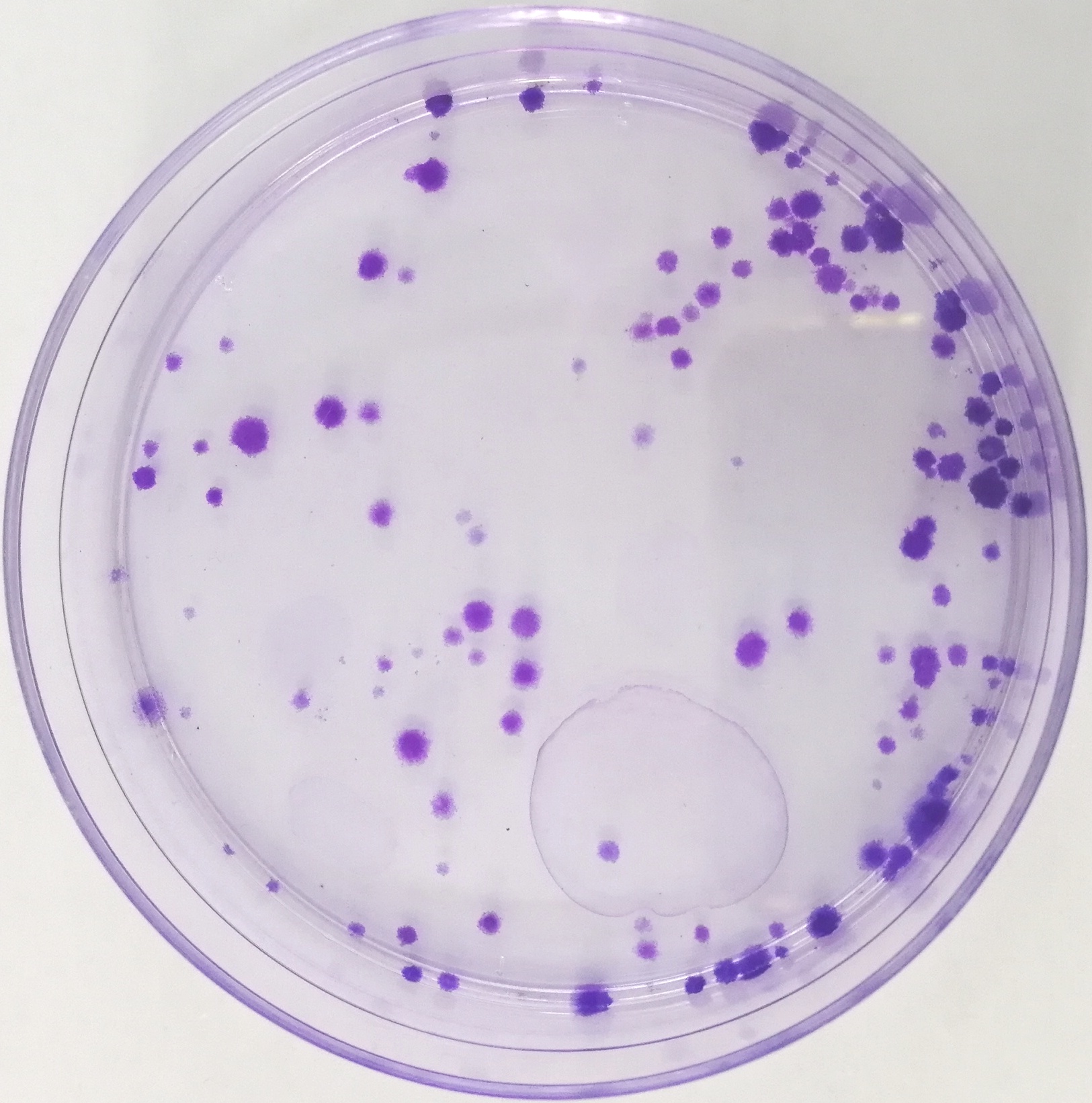

Supplement: Data S1 [file peerj-11-15559-s003.zip › Raw data 1/Raw figure 1-3/Figure 3/Fig 3C AGS sh-NA.jpg]

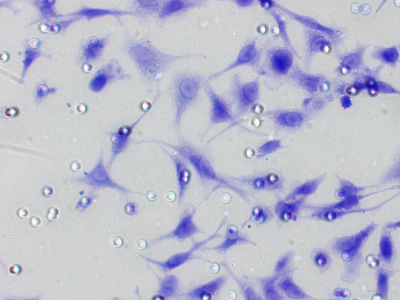

Supplement: Data S1 [file peerj-11-15559-s003.zip › Raw data 1/Raw figure 1-3/Figure 3/Fig 3D 823 Invasion sh-EDIL3.tif]

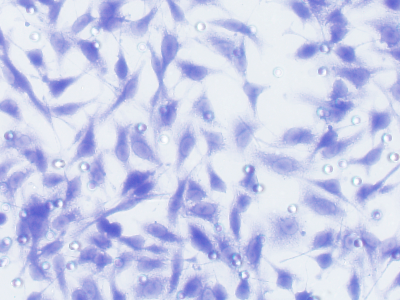

Supplement: Data S1 [file peerj-11-15559-s003.zip › Raw data 1/Raw figure 1-3/Figure 3/Fig 3D 823 Invasion sh-NC.tif]

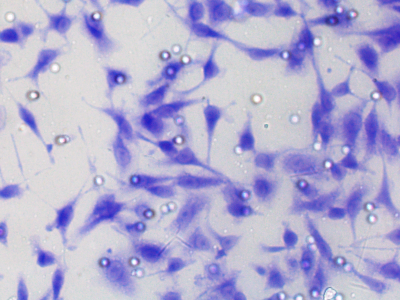

Supplement: Data S1 [file peerj-11-15559-s003.zip › Raw data 1/Raw figure 1-3/Figure 3/Fig 3D 823 migration sh-EDIL3.tif]

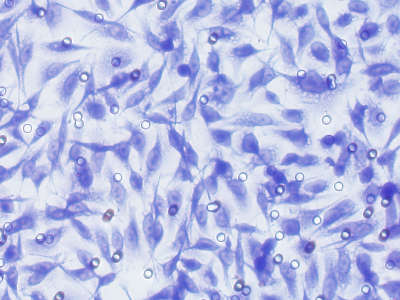

Supplement: Data S1 [file peerj-11-15559-s003.zip › Raw data 1/Raw figure 1-3/Figure 3/Fig 3D 823 migration sh-NA.tif]

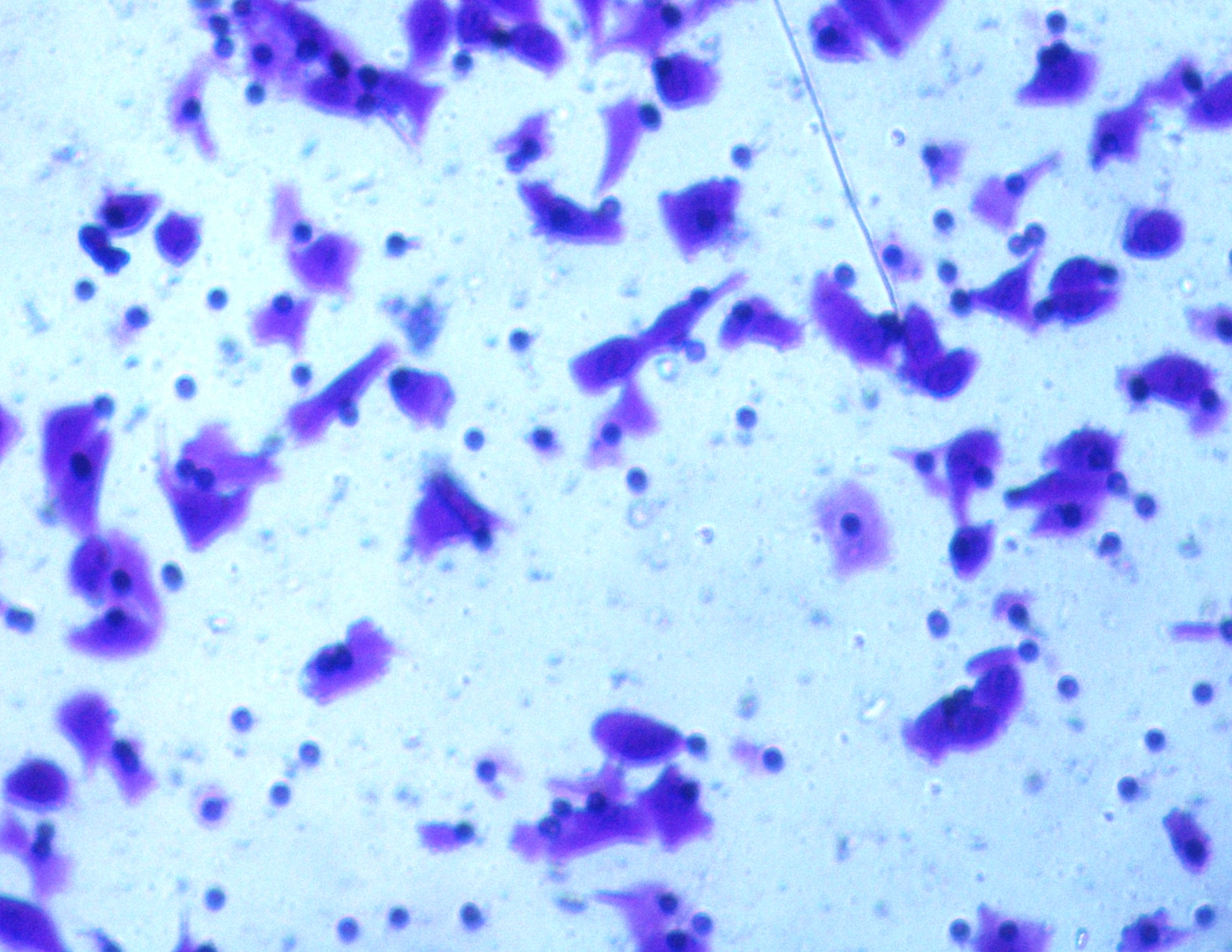

Supplement: Data S1 [file peerj-11-15559-s003.zip › Raw data 1/Raw figure 1-3/Figure 3/Fig 3D AGS invasion sh-EDIL3.jpg]

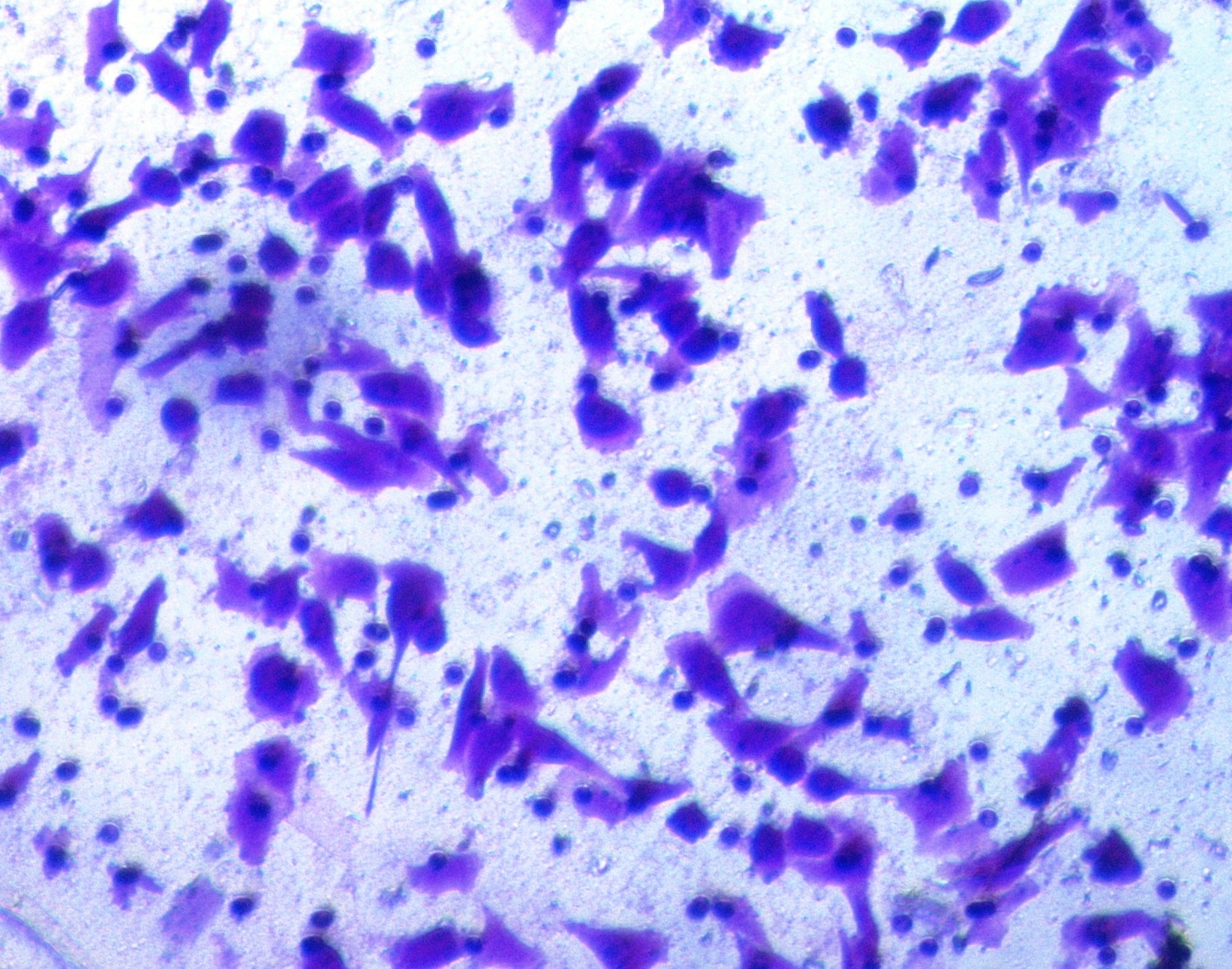

Supplement: Data S1 [file peerj-11-15559-s003.zip › Raw data 1/Raw figure 1-3/Figure 3/Fig 3D AGS invasion sh-NA.jpg]

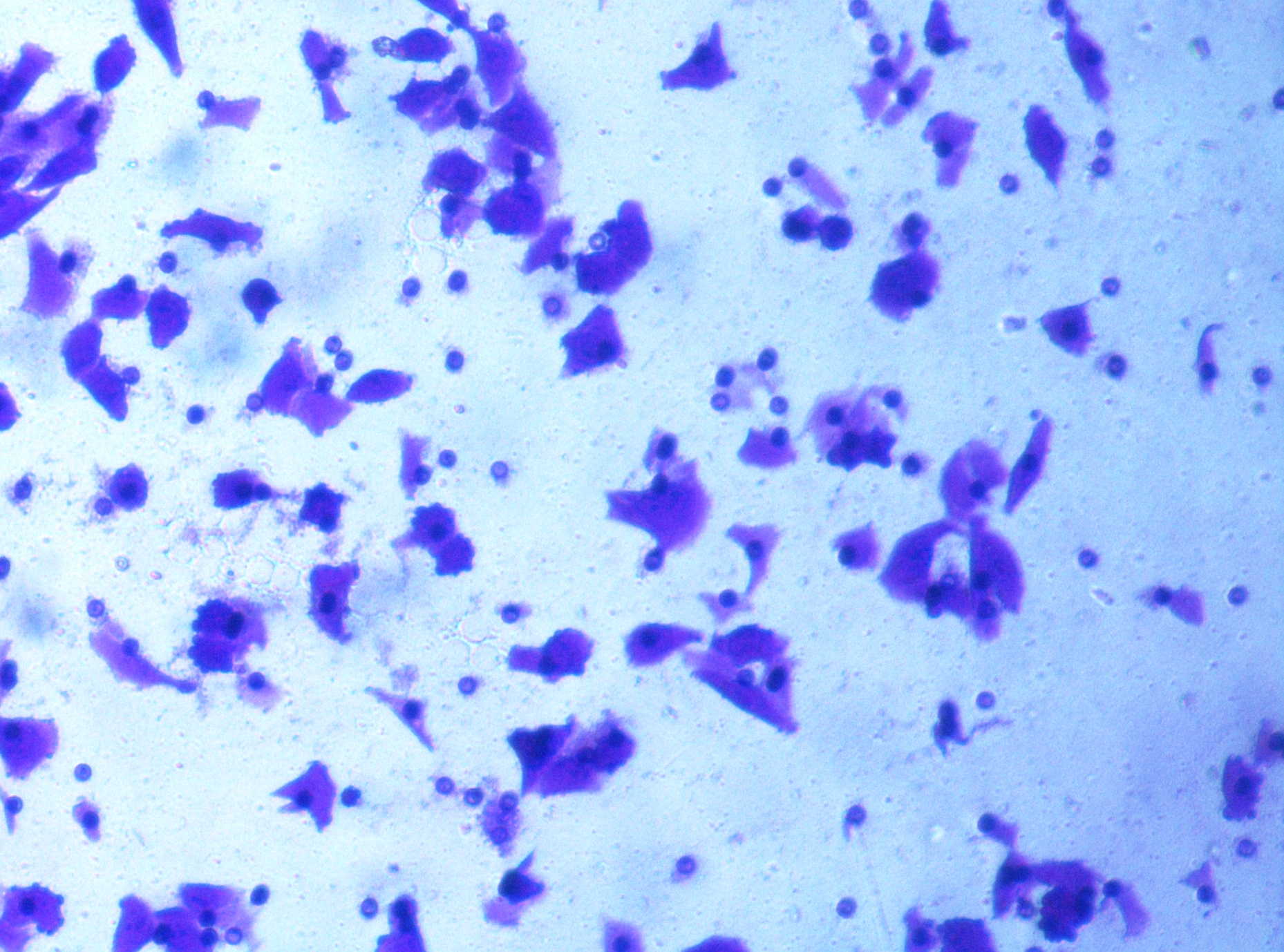

Supplement: Data S1 [file peerj-11-15559-s003.zip › Raw data 1/Raw figure 1-3/Figure 3/Fig 3D AGS migration sh-EDIL3.jpg]

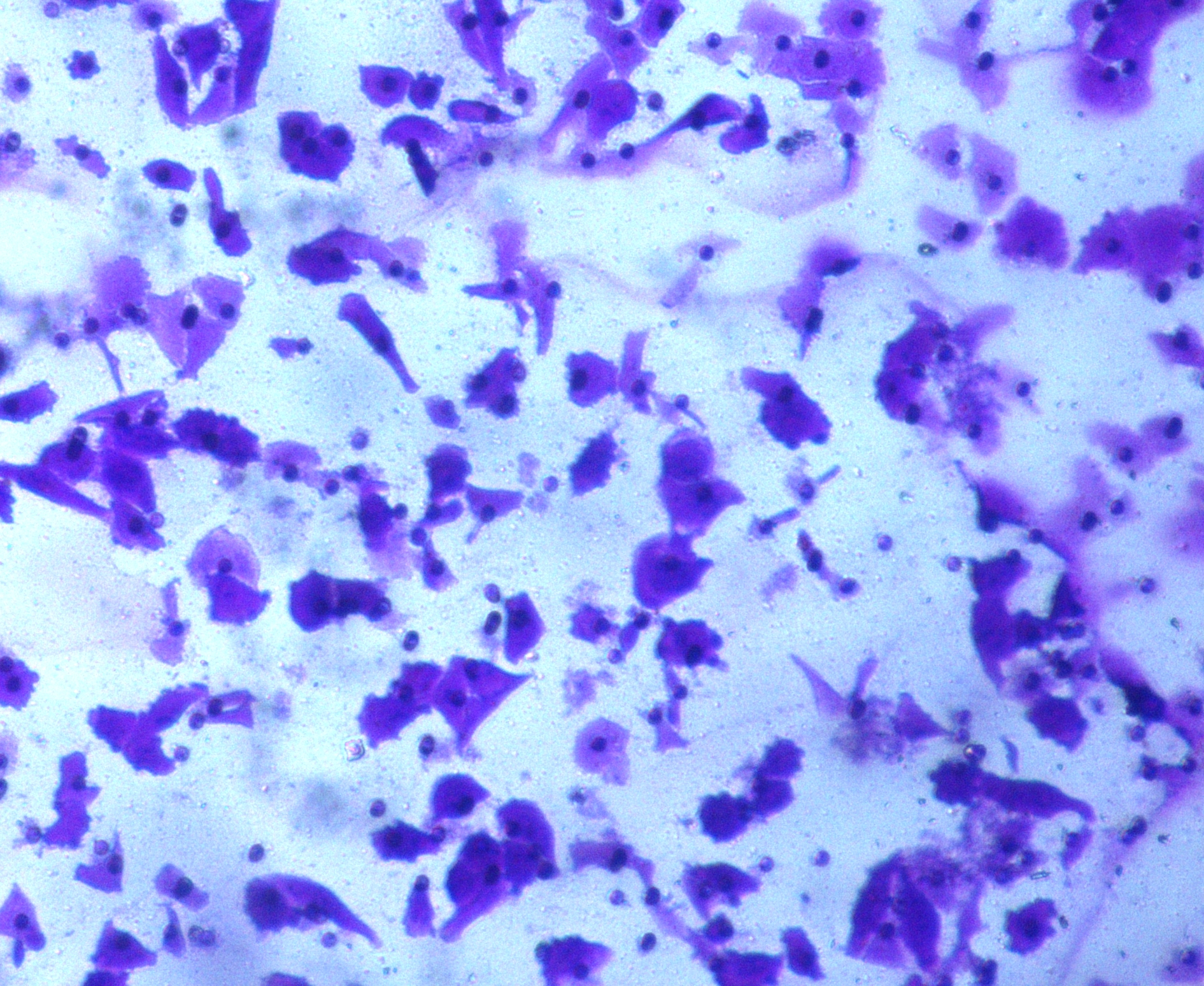

Supplement: Data S1 [file peerj-11-15559-s003.zip › Raw data 1/Raw figure 1-3/Figure 3/Fig 3D AGS migration sh-NA.jpg]

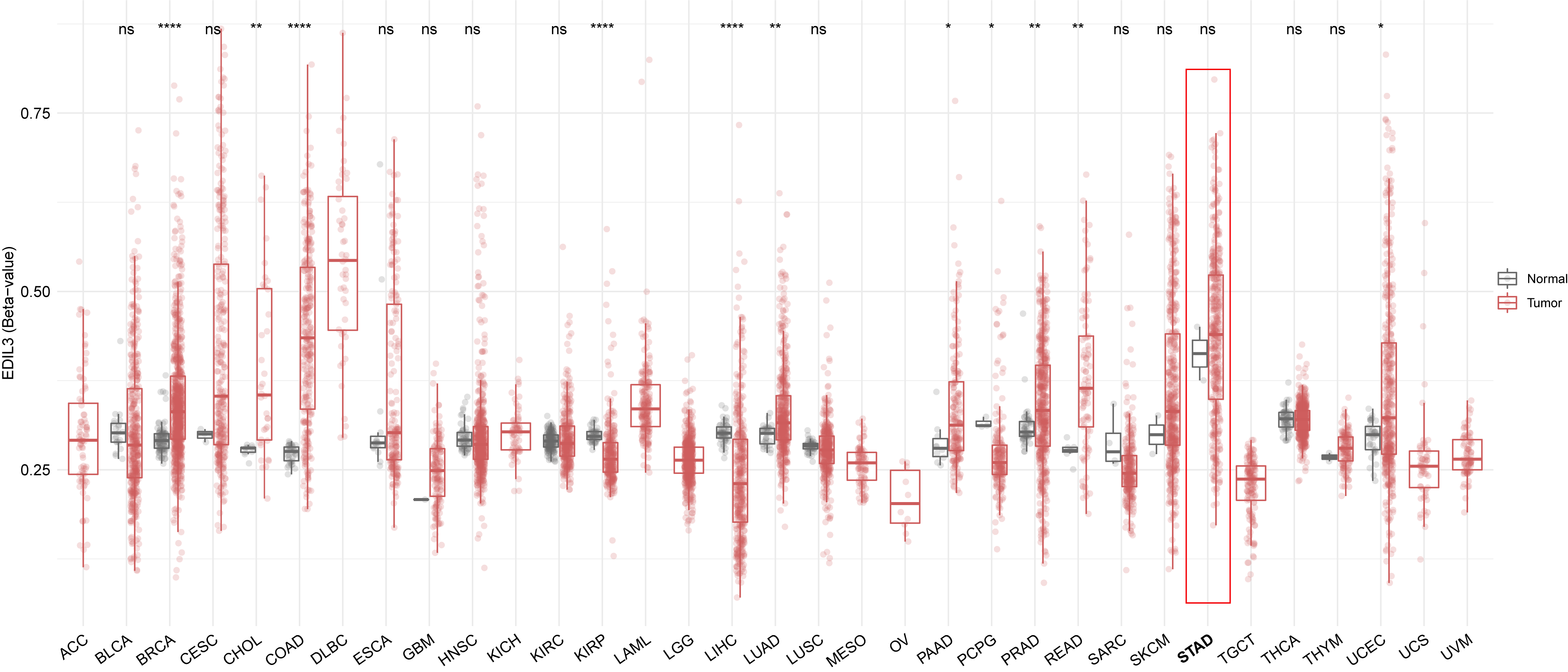

Supplement: Data S2 [file peerj-11-15559-s004.zip › Raw data 2/Raw figure 4-10/Figure 4/Fig 4A Aggregation-methylation-2022-03-16.tif]

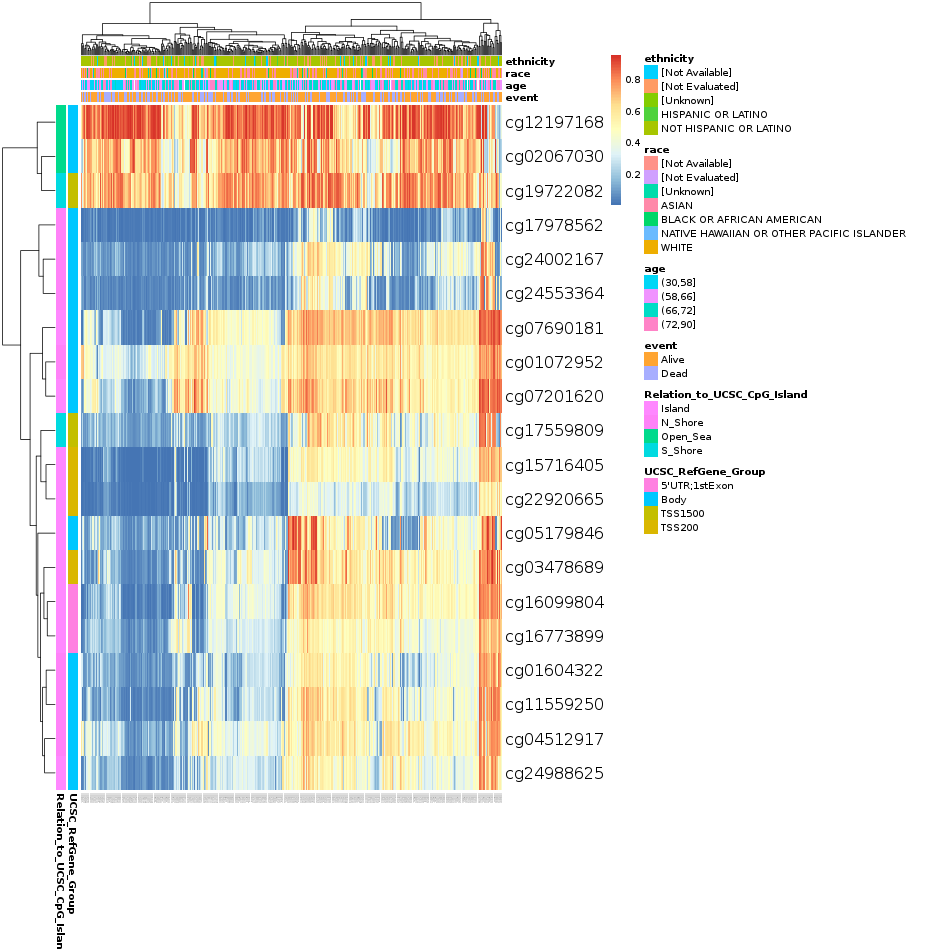

Supplement: Data S2 [file peerj-11-15559-s004.zip › Raw data 2/Raw figure 4-10/Figure 4/Fig 4B heatmap.png]

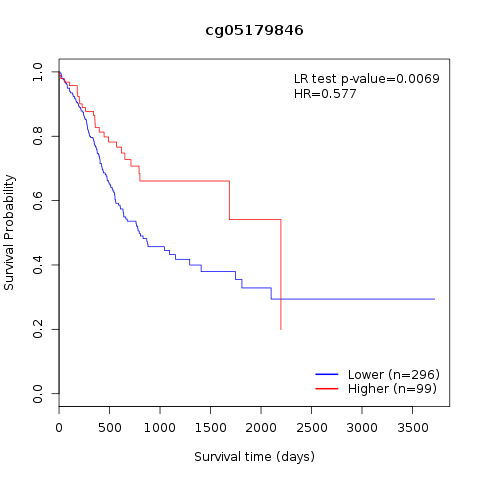

Supplement: Data S2 [file peerj-11-15559-s004.zip › Raw data 2/Raw figure 4-10/Figure 4/Fig 4C survplot cg05179846.png]

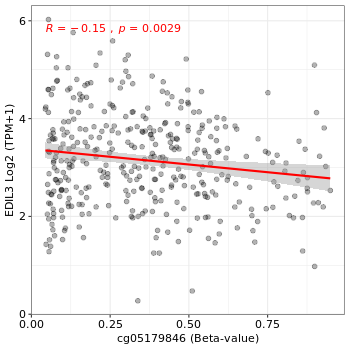

Supplement: Data S2 [file peerj-11-15559-s004.zip › Raw data 2/Raw figure 4-10/Figure 4/Fig 4D cg05179846╧┬╘╪.png]

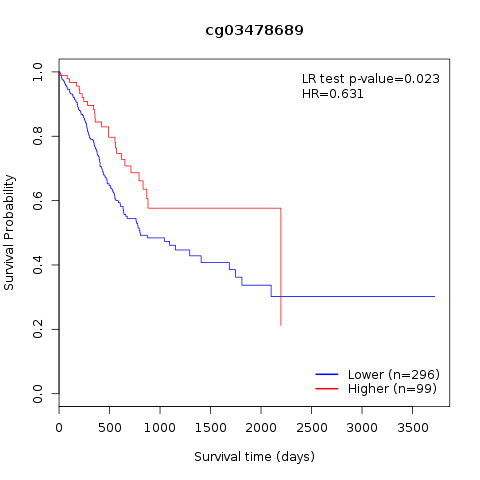

Supplement: Data S2 [file peerj-11-15559-s004.zip › Raw data 2/Raw figure 4-10/Figure 4/Fig 4E survplot cg03478689.png]

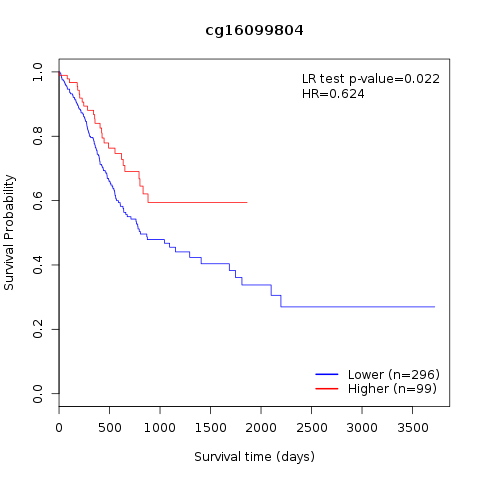

Supplement: Data S2 [file peerj-11-15559-s004.zip › Raw data 2/Raw figure 4-10/Figure 4/Fig 4G survplot cg16099804.png]

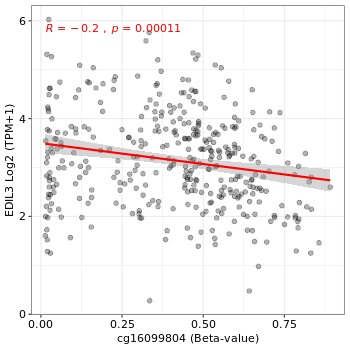

Supplement: Data S2 [file peerj-11-15559-s004.zip › Raw data 2/Raw figure 4-10/Figure 4/Fig 4H cg16099804╧┬╘╪.png]

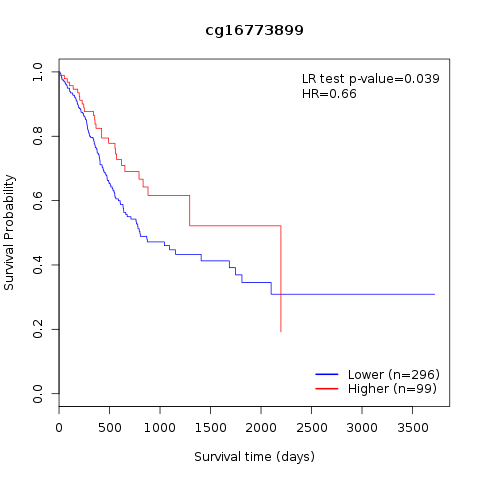

Supplement: Data S2 [file peerj-11-15559-s004.zip › Raw data 2/Raw figure 4-10/Figure 4/Fig 4I survplot cg16773899.png]

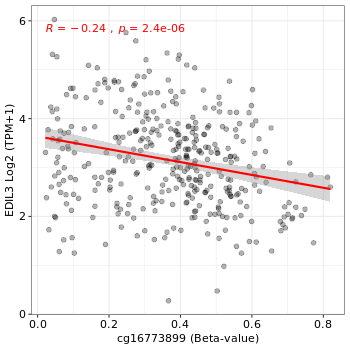

Supplement: Data S2 [file peerj-11-15559-s004.zip › Raw data 2/Raw figure 4-10/Figure 4/Fig 4J cg16773899 ╧┬╘╪.png]

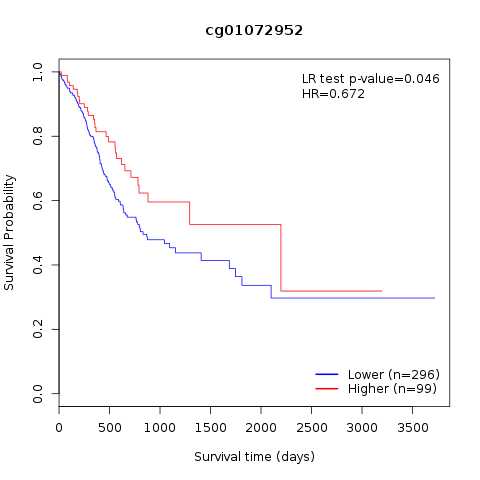

Supplement: Data S2 [file peerj-11-15559-s004.zip › Raw data 2/Raw figure 4-10/Figure 4/Fig 4K survplot cg01072952.png]

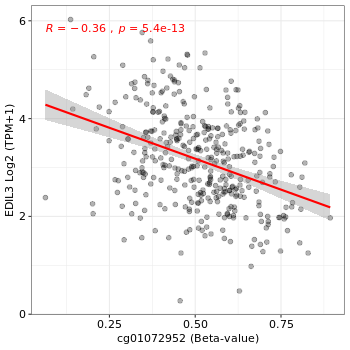

Supplement: Data S2 [file peerj-11-15559-s004.zip › Raw data 2/Raw figure 4-10/Figure 4/Fig 4L cg01072952╧┬╘╪.png]

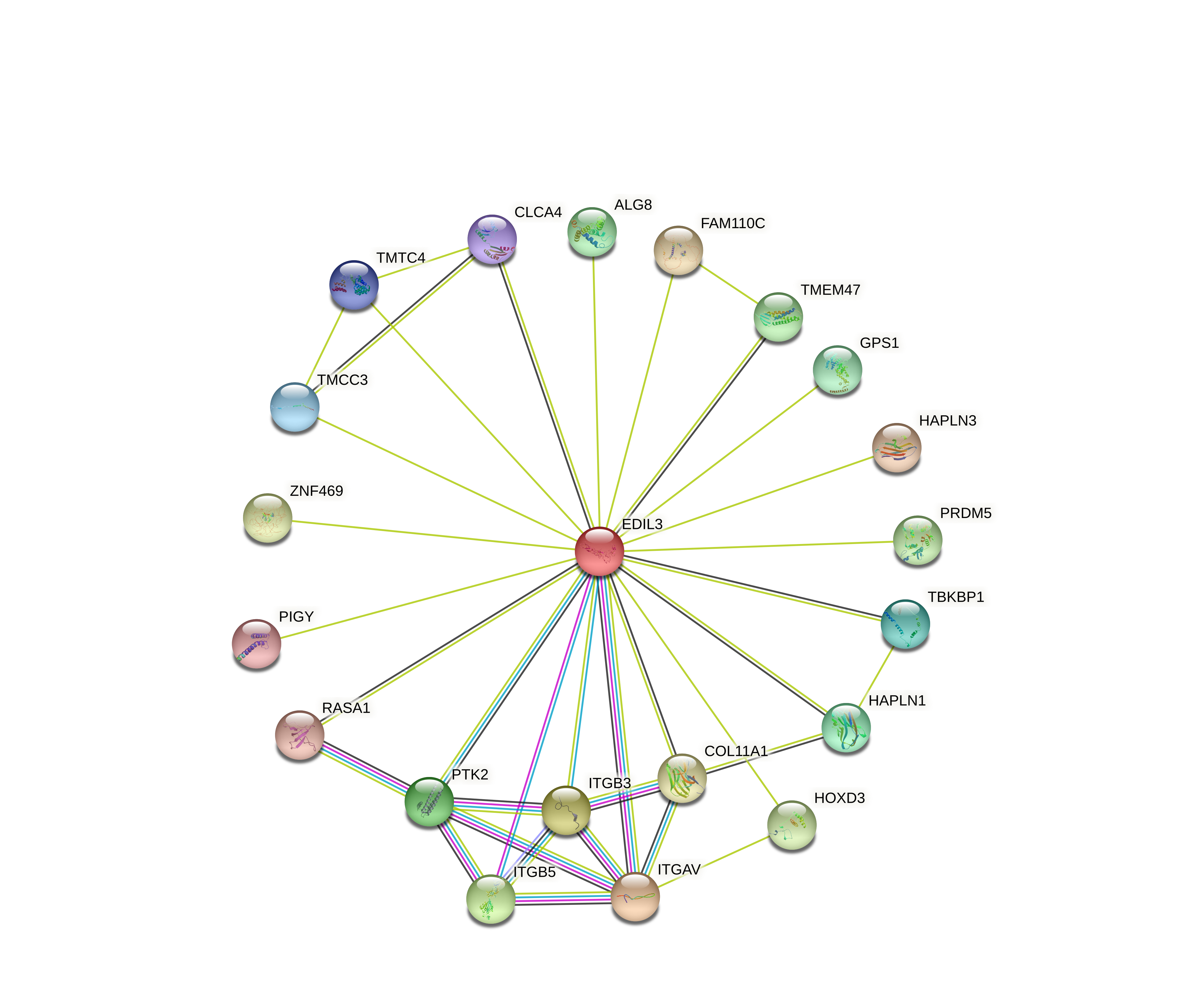

Supplement: Data S2 [file peerj-11-15559-s004.zip › Raw data 2/Raw figure 4-10/Figure 5/Fig 5A.png]

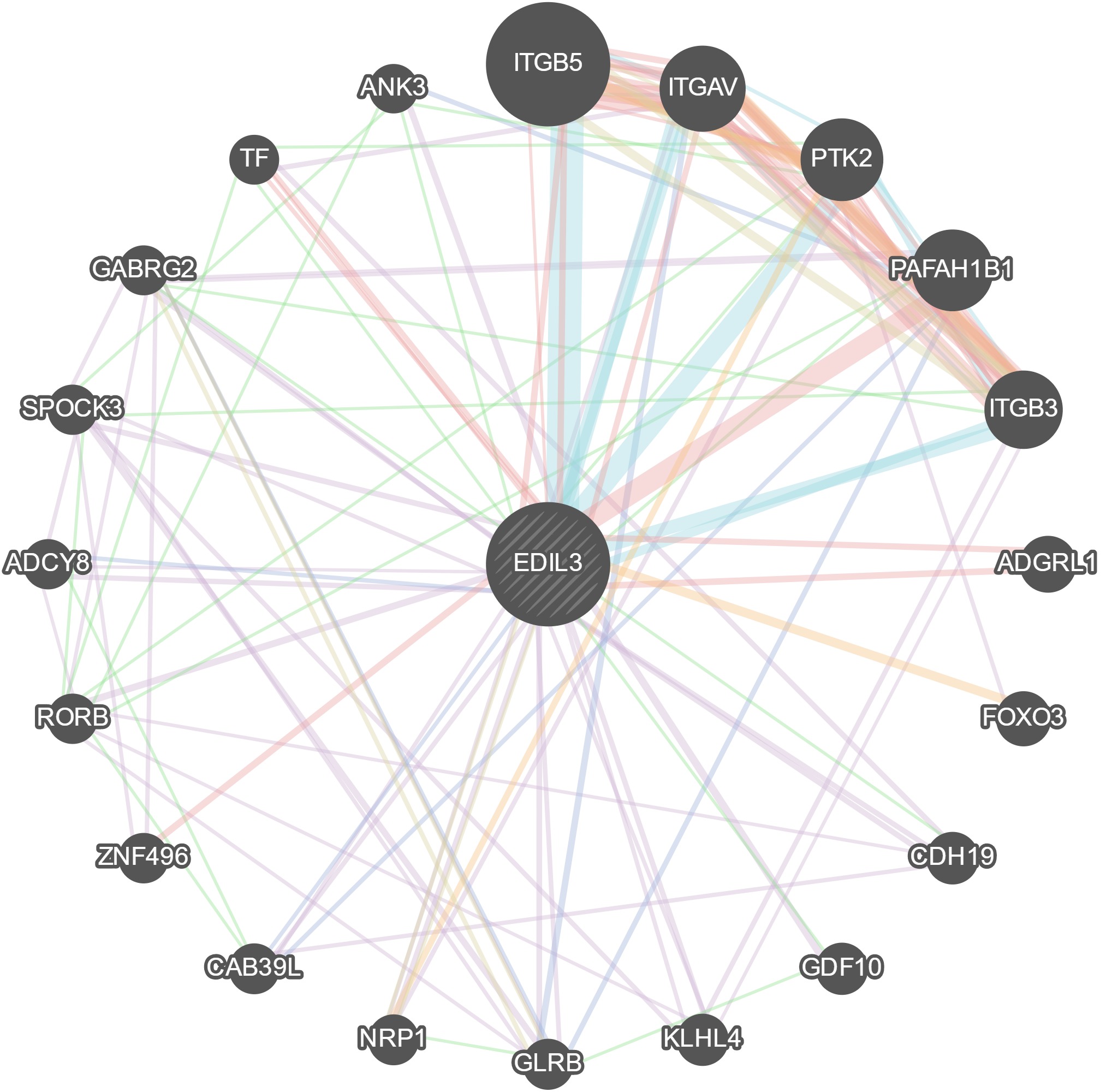

Supplement: Data S2 [file peerj-11-15559-s004.zip › Raw data 2/Raw figure 4-10/Figure 5/Fig 5B.jpg]

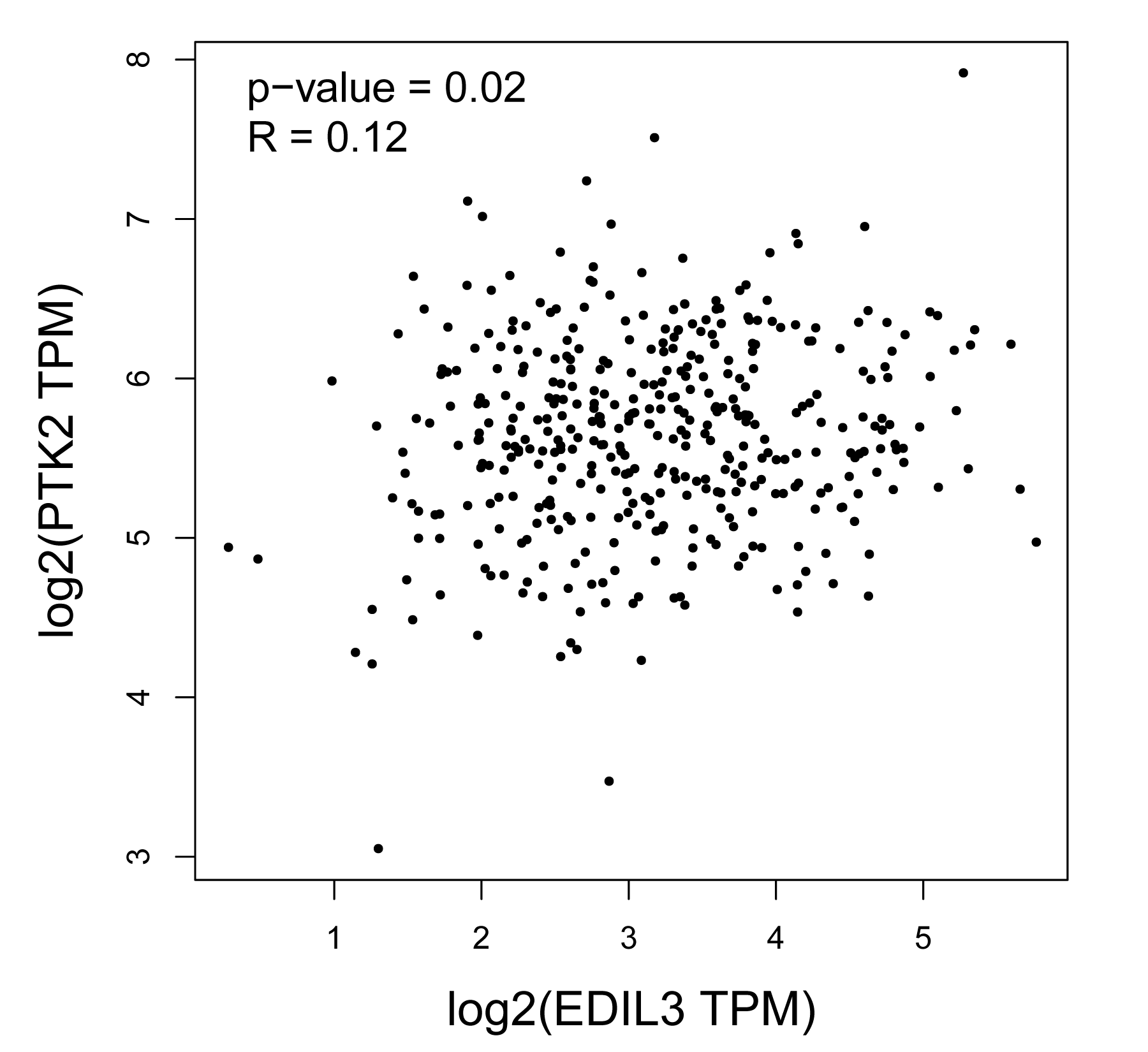

Supplement: Data S2 [file peerj-11-15559-s004.zip › Raw data 2/Raw figure 4-10/Figure 5/Fig 5C EDIL3_PTK2_correlation_o0qYE-01.tif]

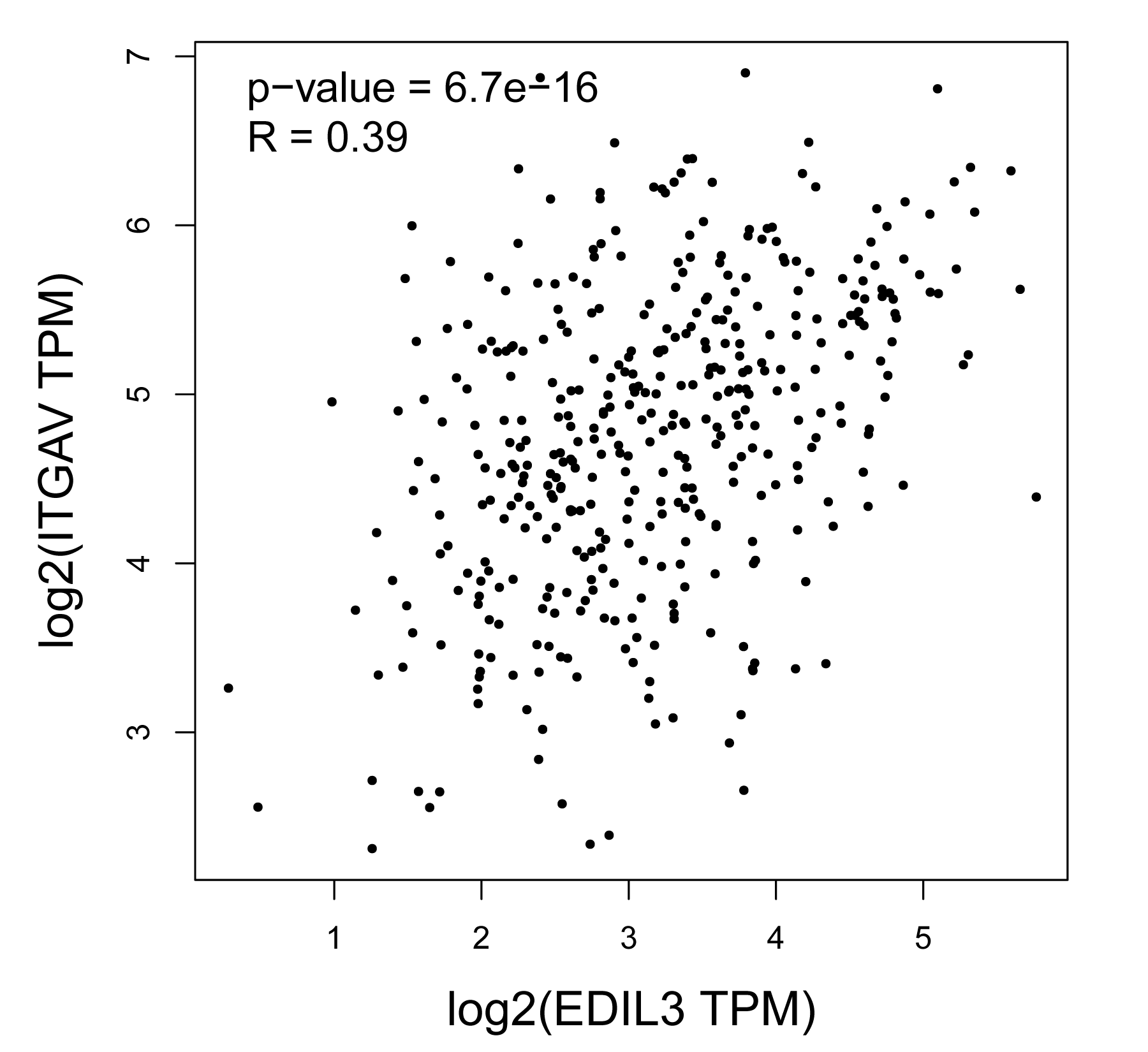

Supplement: Data S2 [file peerj-11-15559-s004.zip › Raw data 2/Raw figure 4-10/Figure 5/Fig 5C EDIL3_ITGAV_correlation_uHu03-01.tif]

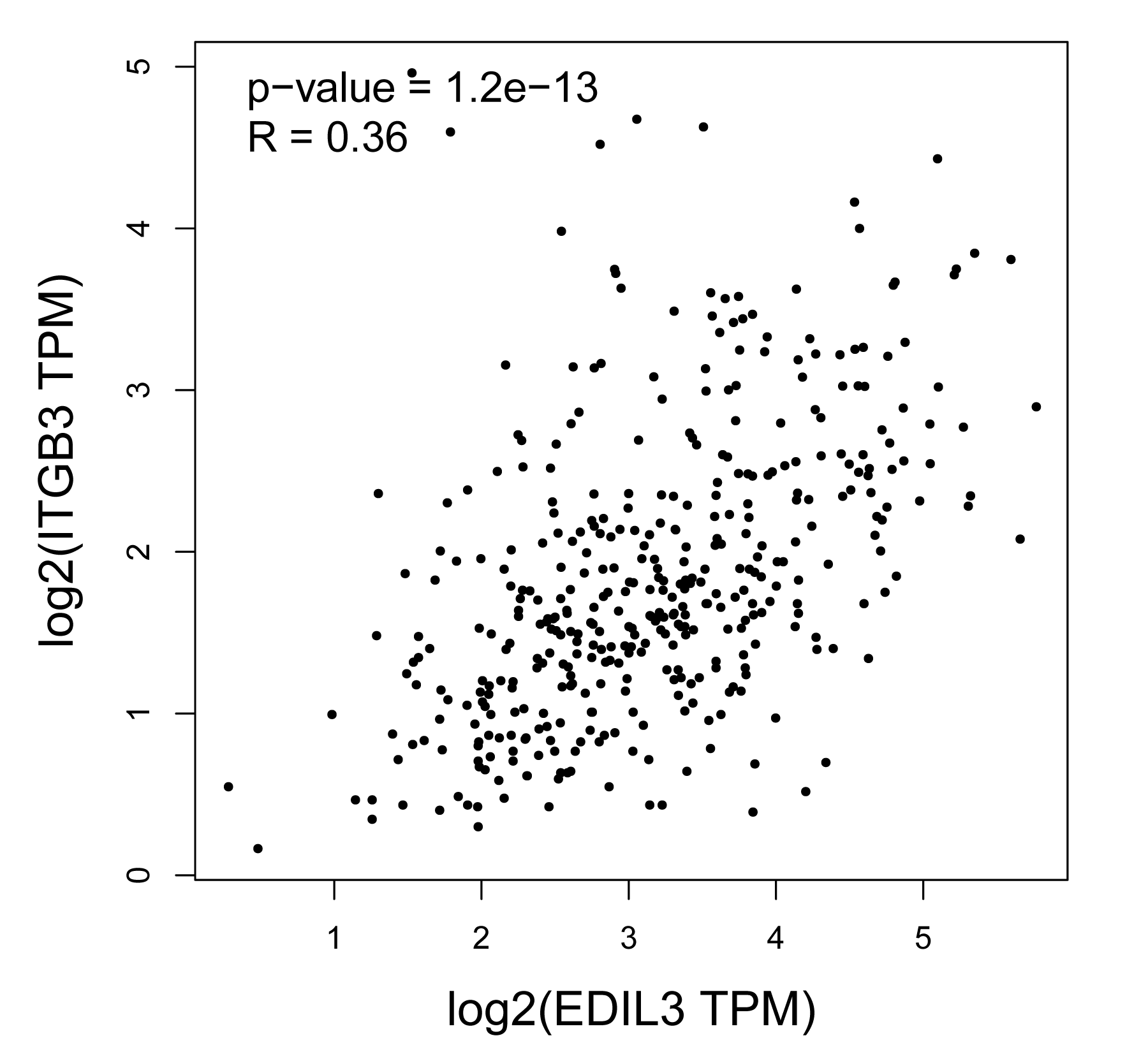

Supplement: Data S2 [file peerj-11-15559-s004.zip › Raw data 2/Raw figure 4-10/Figure 5/Fig 5C EDIL3_ITGB3_correlation_LvCZa-01.tif]

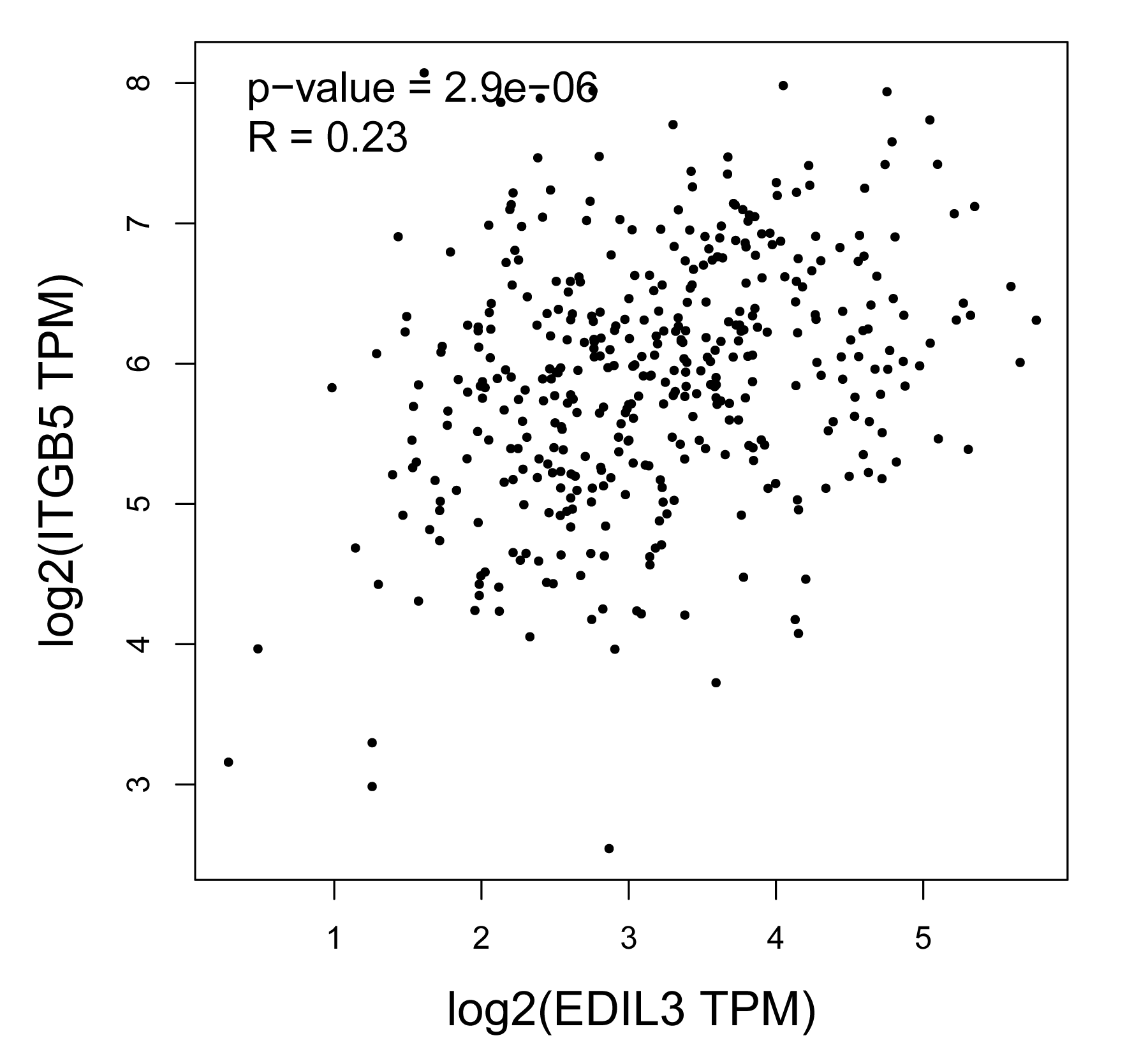

Supplement: Data S2 [file peerj-11-15559-s004.zip › Raw data 2/Raw figure 4-10/Figure 5/Fig 5C EDIL3_ITGB5_correlation_0qNMu-01.tif]

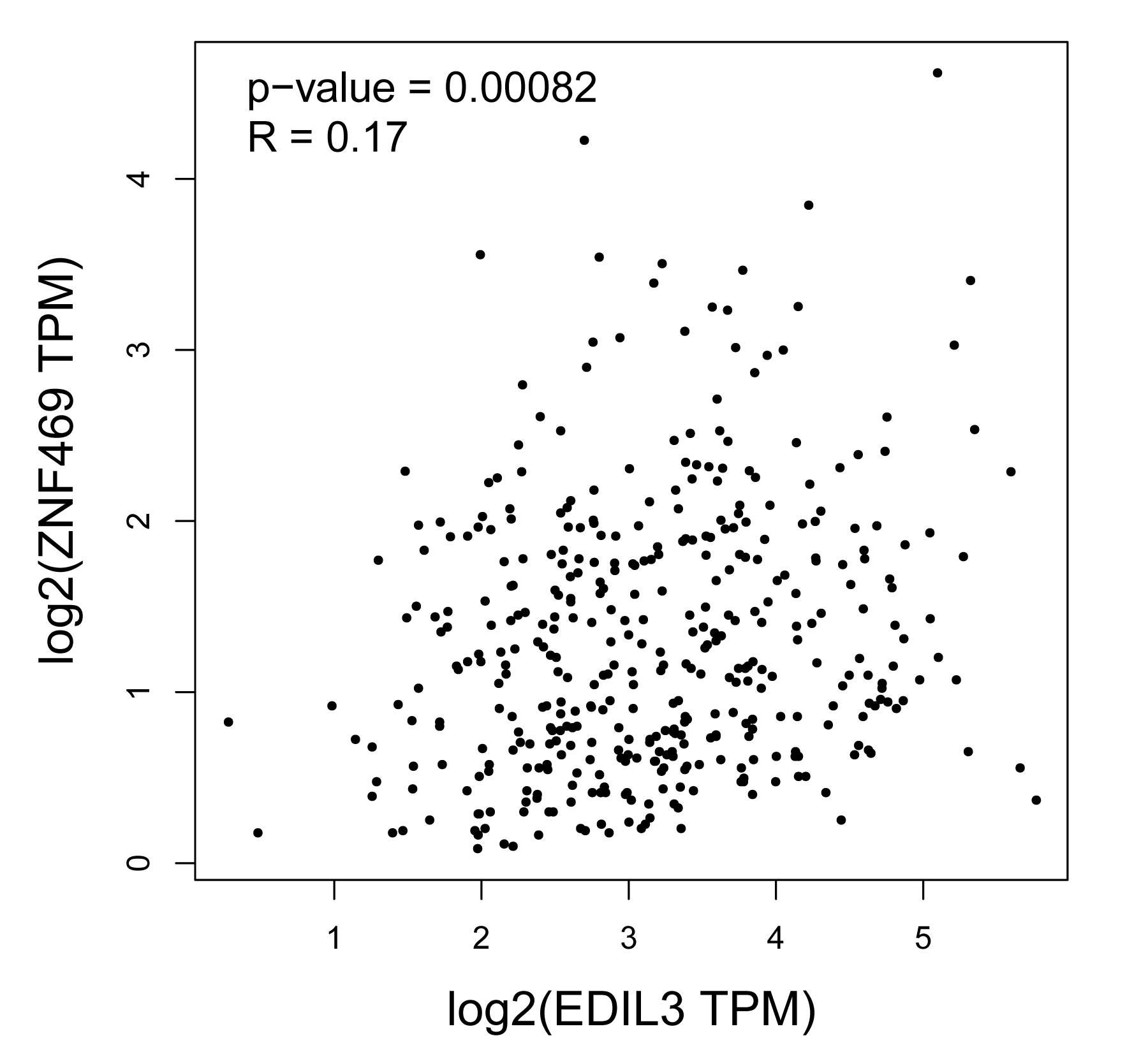

Supplement: Data S2 [file peerj-11-15559-s004.zip › Raw data 2/Raw figure 4-10/Figure 5/Fig 5C EDIL3_ZNF469_correlation_7vlCo-01.tif]

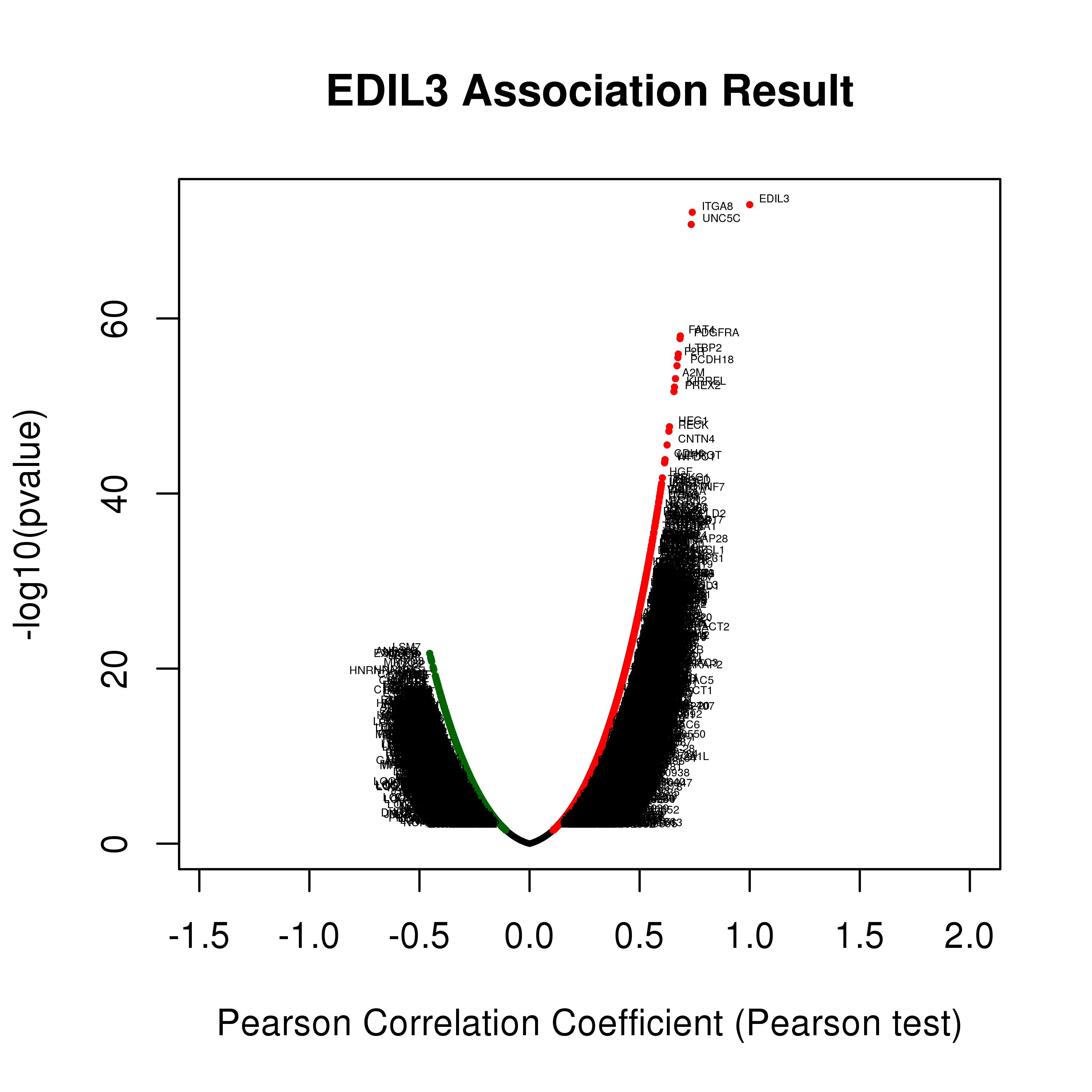

Supplement: Data S2 [file peerj-11-15559-s004.zip › Raw data 2/Raw figure 4-10/Figure 6/Fig 6A.png]

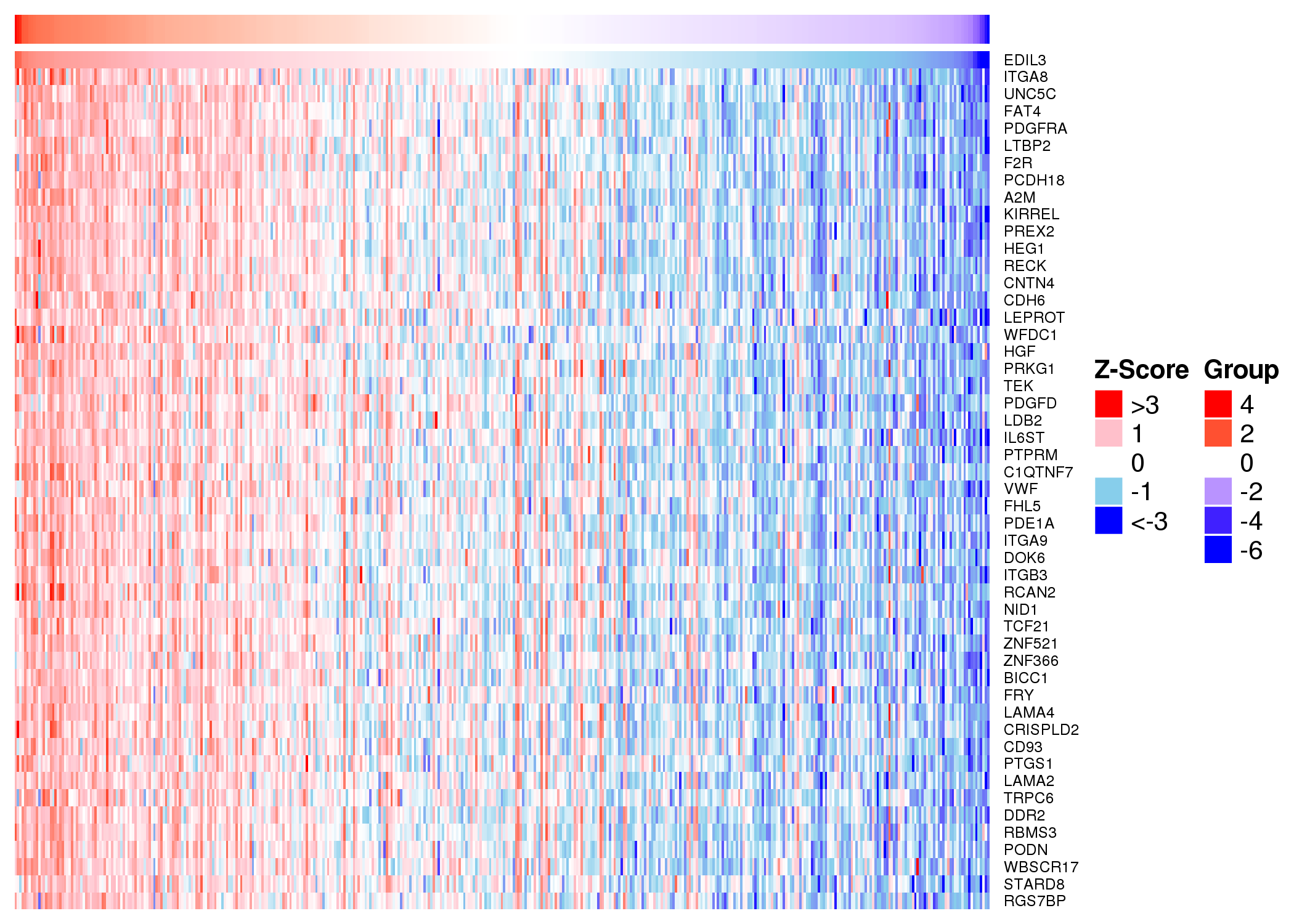

Supplement: Data S2 [file peerj-11-15559-s004.zip › Raw data 2/Raw figure 4-10/Figure 6/Fig 6B positive.png]

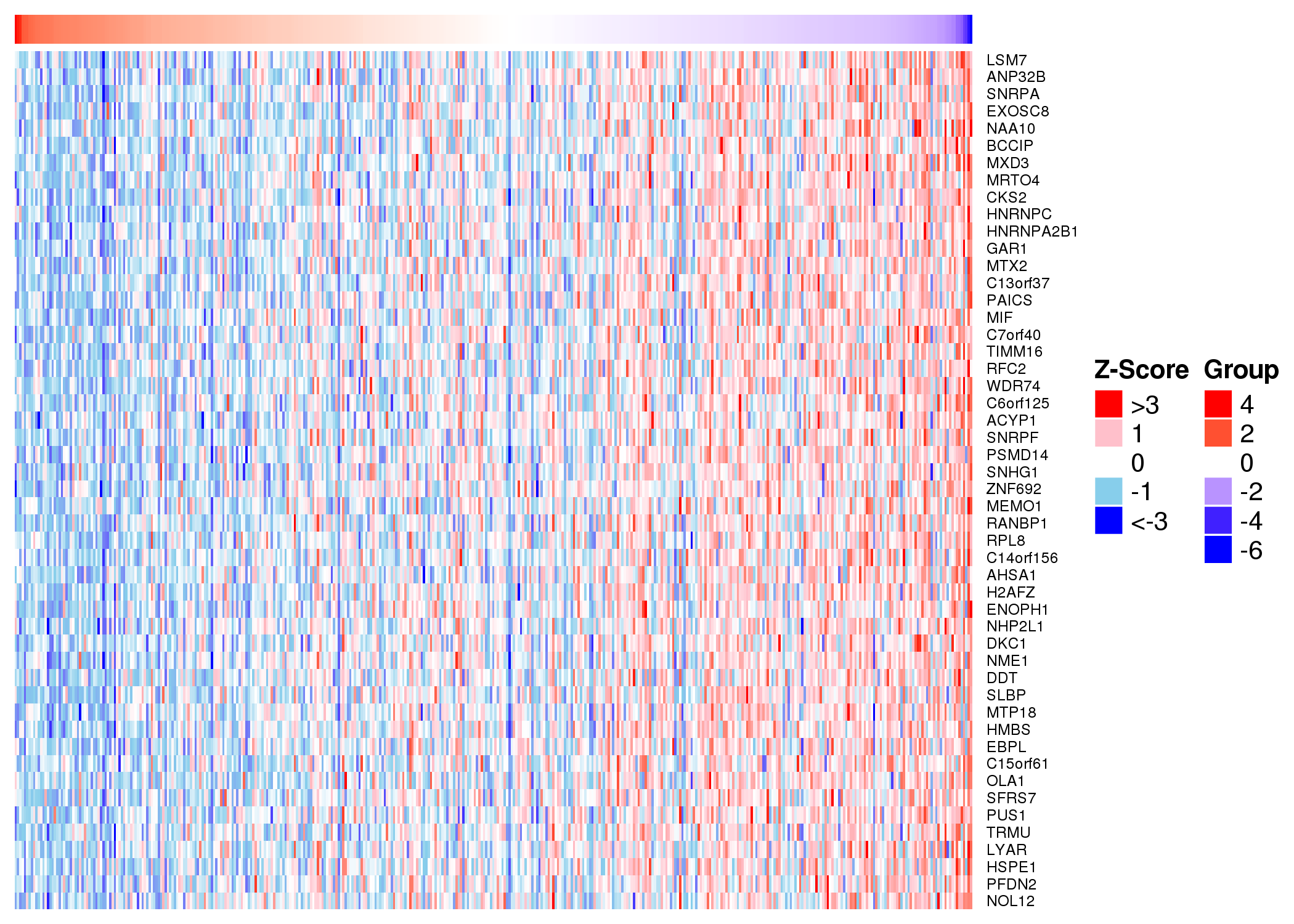

Supplement: Data S2 [file peerj-11-15559-s004.zip › Raw data 2/Raw figure 4-10/Figure 6/Fig 6C negative.png]

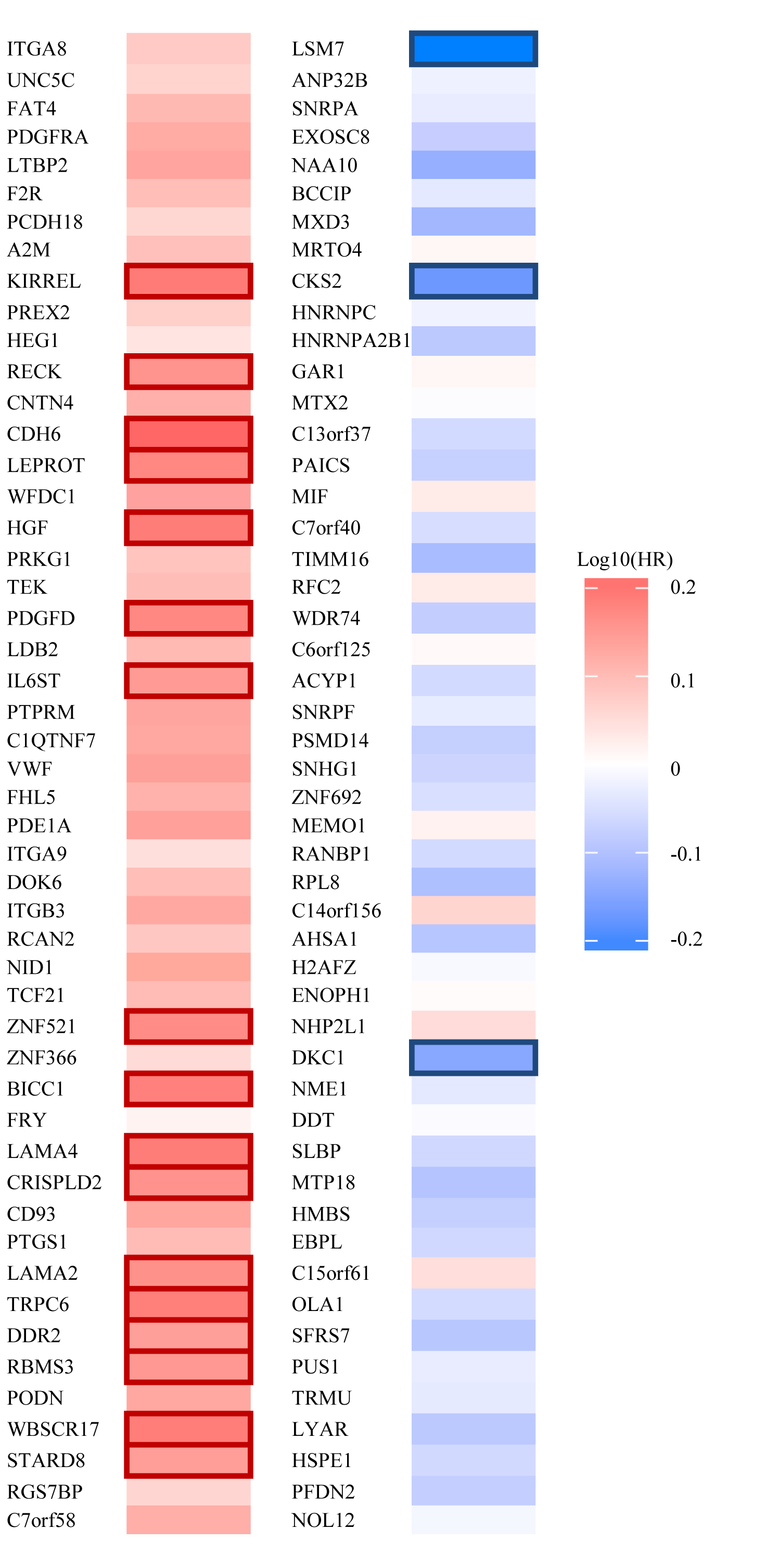

Supplement: Data S2 [file peerj-11-15559-s004.zip › Raw data 2/Raw figure 4-10/Figure 6/Fig 6D.tif]

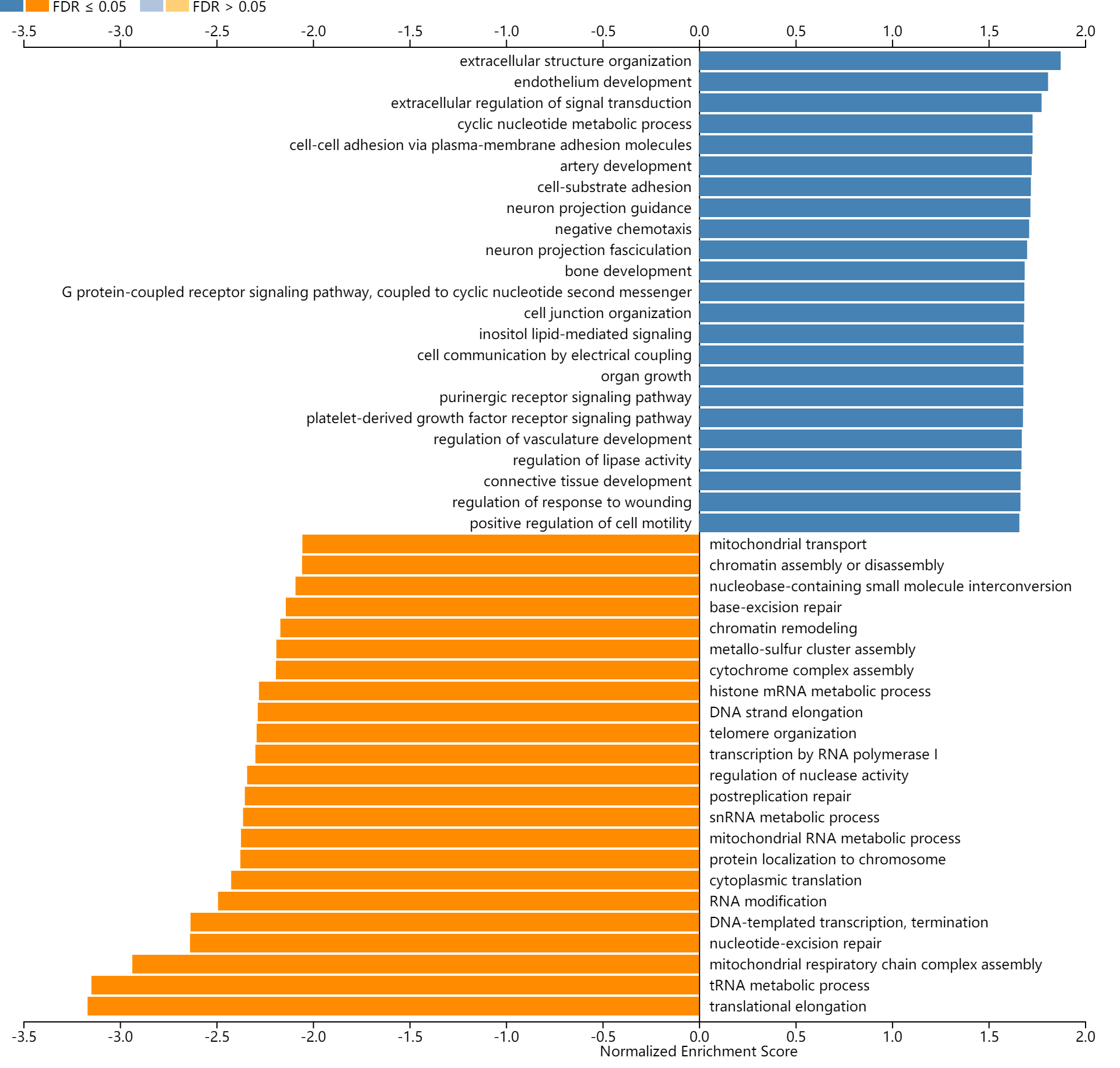

Supplement: Data S2 [file peerj-11-15559-s004.zip › Raw data 2/Raw figure 4-10/Figure 6/Fig 6E Go.png]

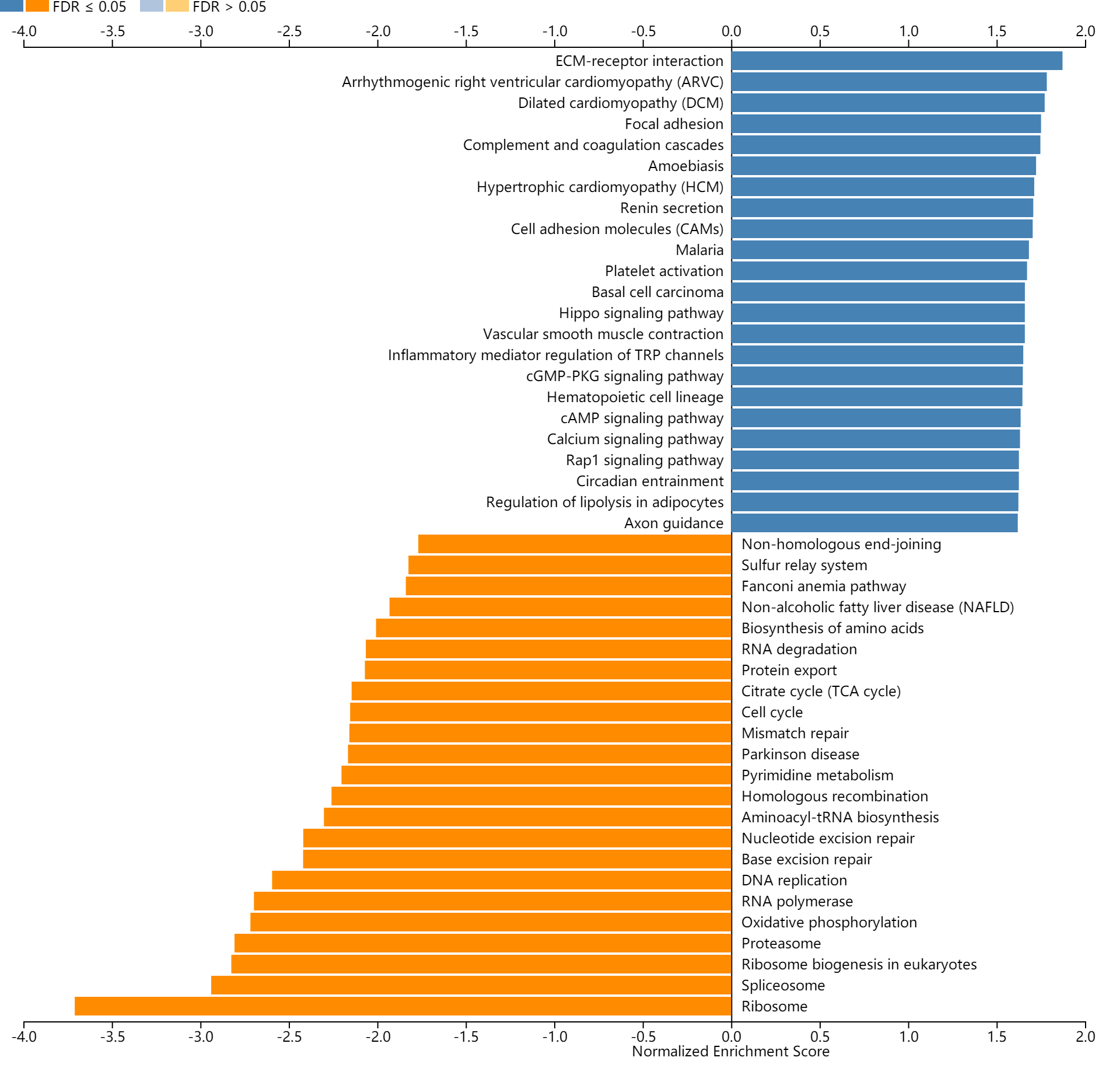

Supplement: Data S2 [file peerj-11-15559-s004.zip › Raw data 2/Raw figure 4-10/Figure 6/Fig 6F KEGG.png]

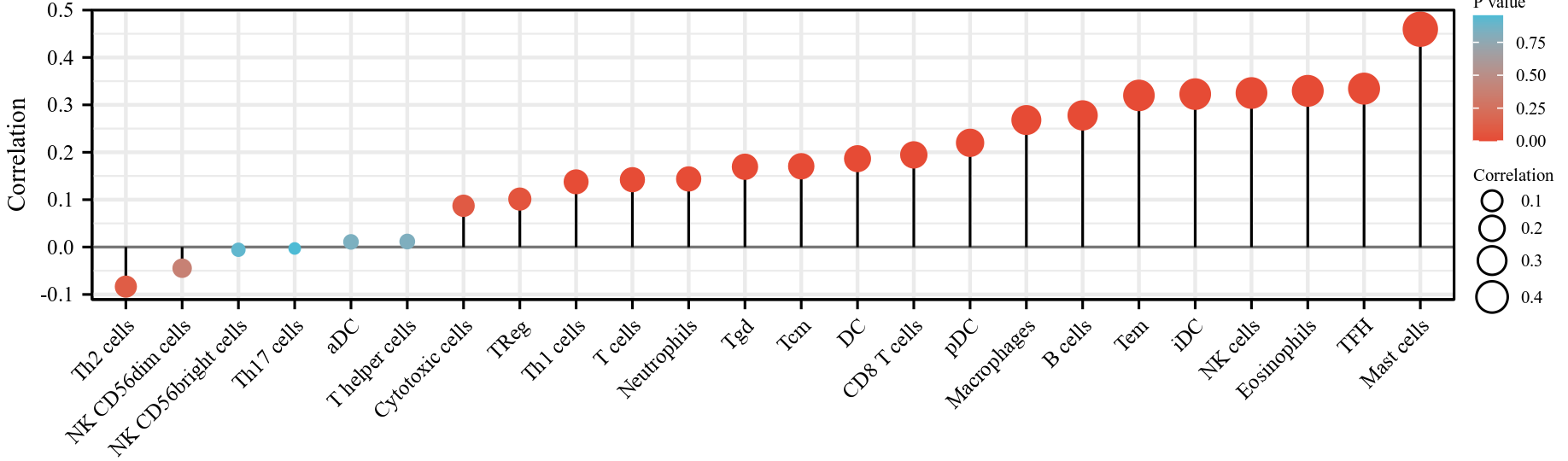

Supplement: Data S2 [file peerj-11-15559-s004.zip › Raw data 2/Raw figure 4-10/Figure 7/Fig 7A.png]

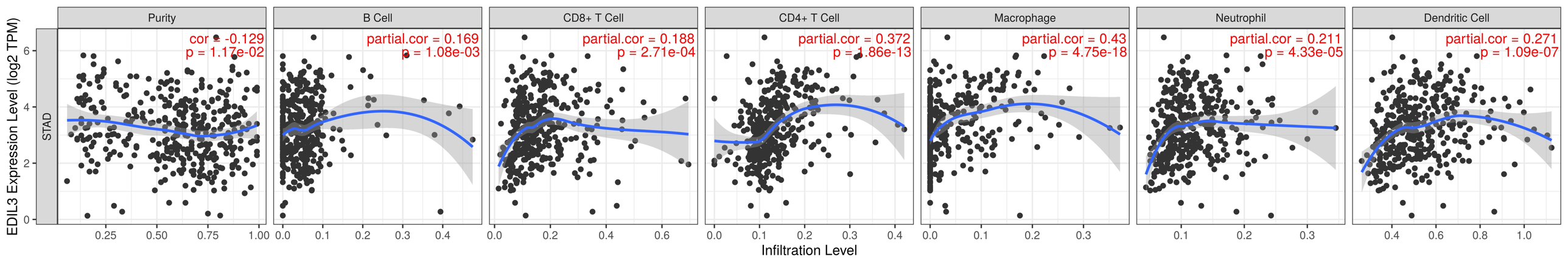

Supplement: Data S2 [file peerj-11-15559-s004.zip › Raw data 2/Raw figure 4-10/Figure 7/Fig 7B.jpg]

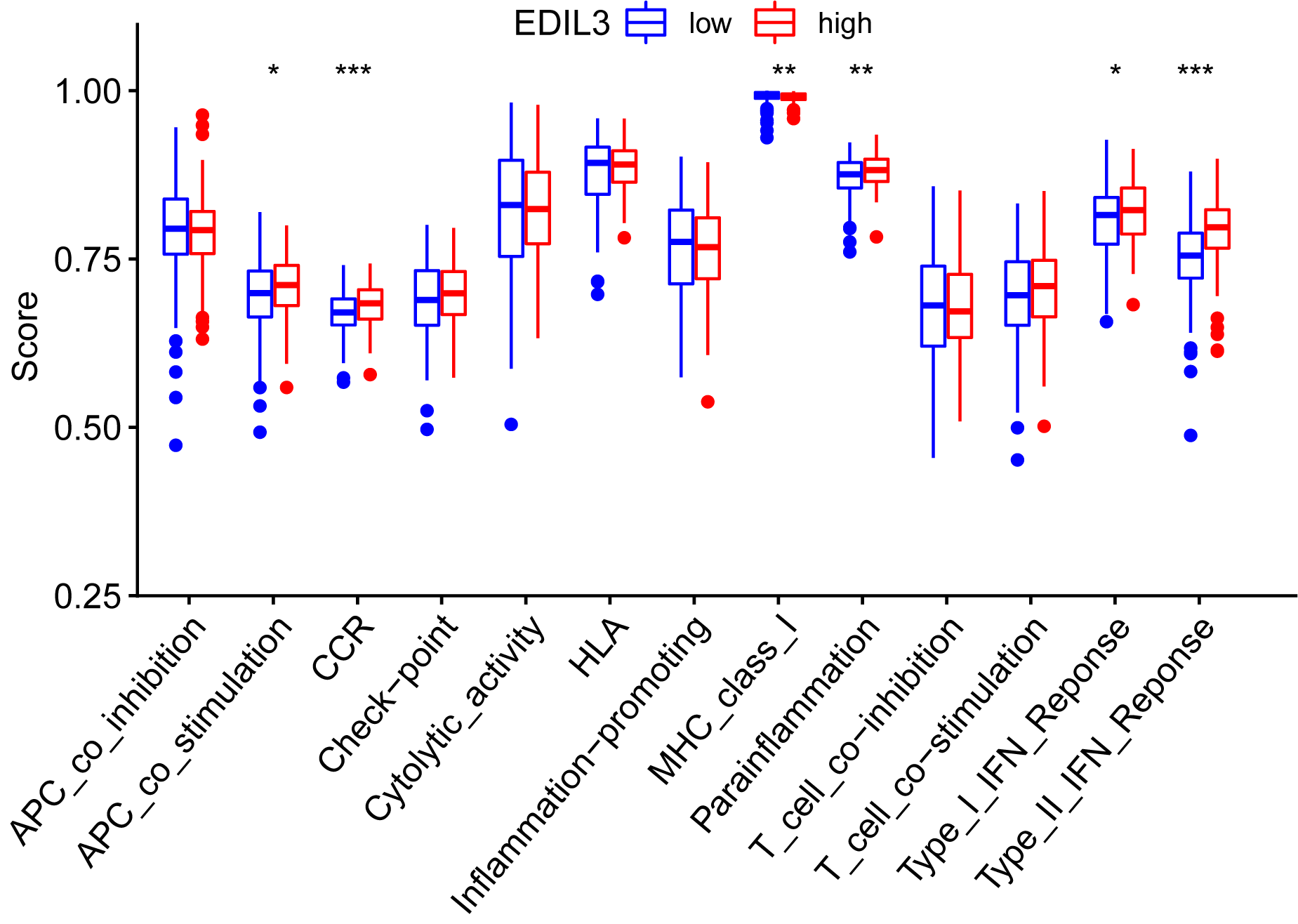

Supplement: Data S2 [file peerj-11-15559-s004.zip › Raw data 2/Raw figure 4-10/Figure 7/Fig 7C.tif]

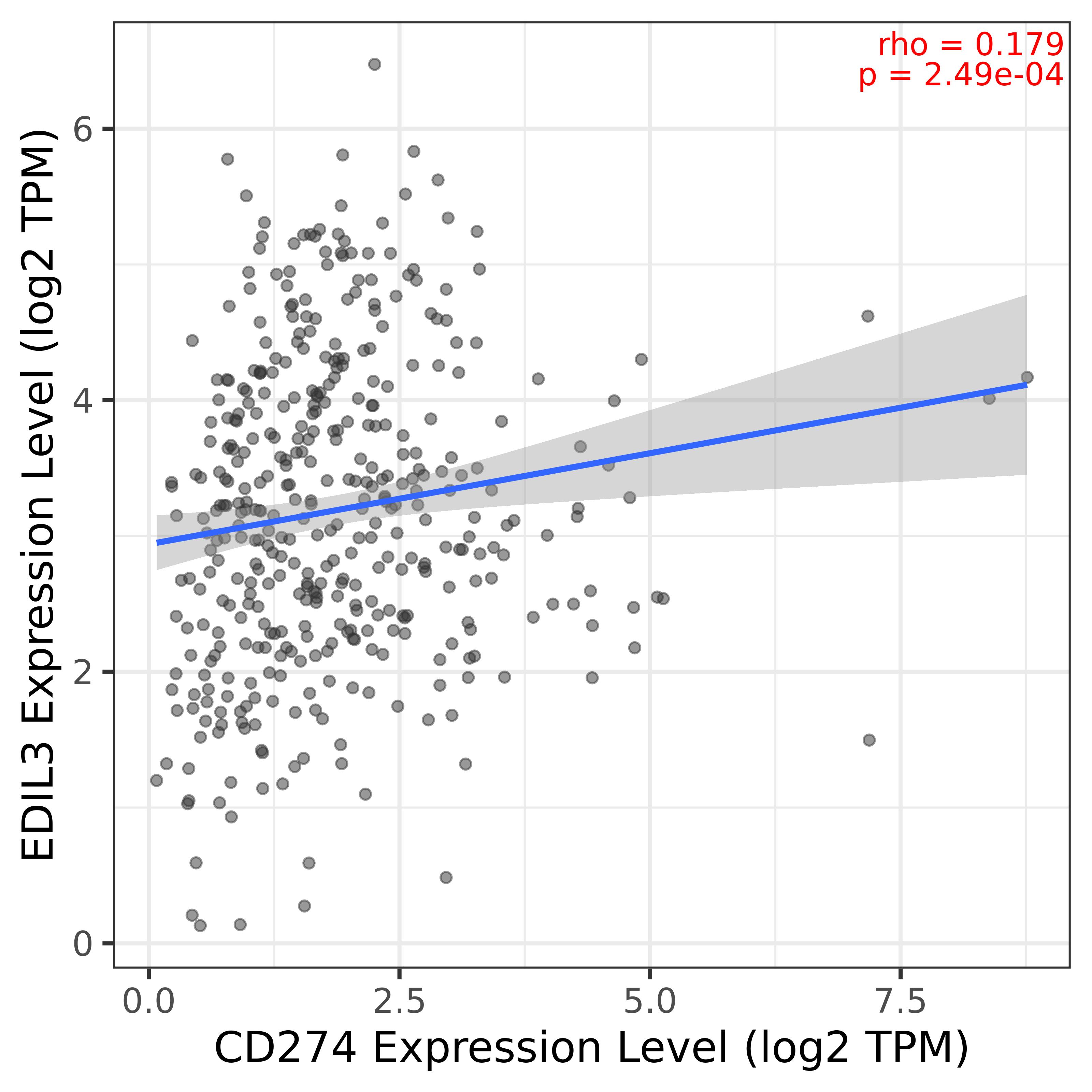

Supplement: Data S2 [file peerj-11-15559-s004.zip › Raw data 2/Raw figure 4-10/Figure 8/Fig 8A CD274.jpg]

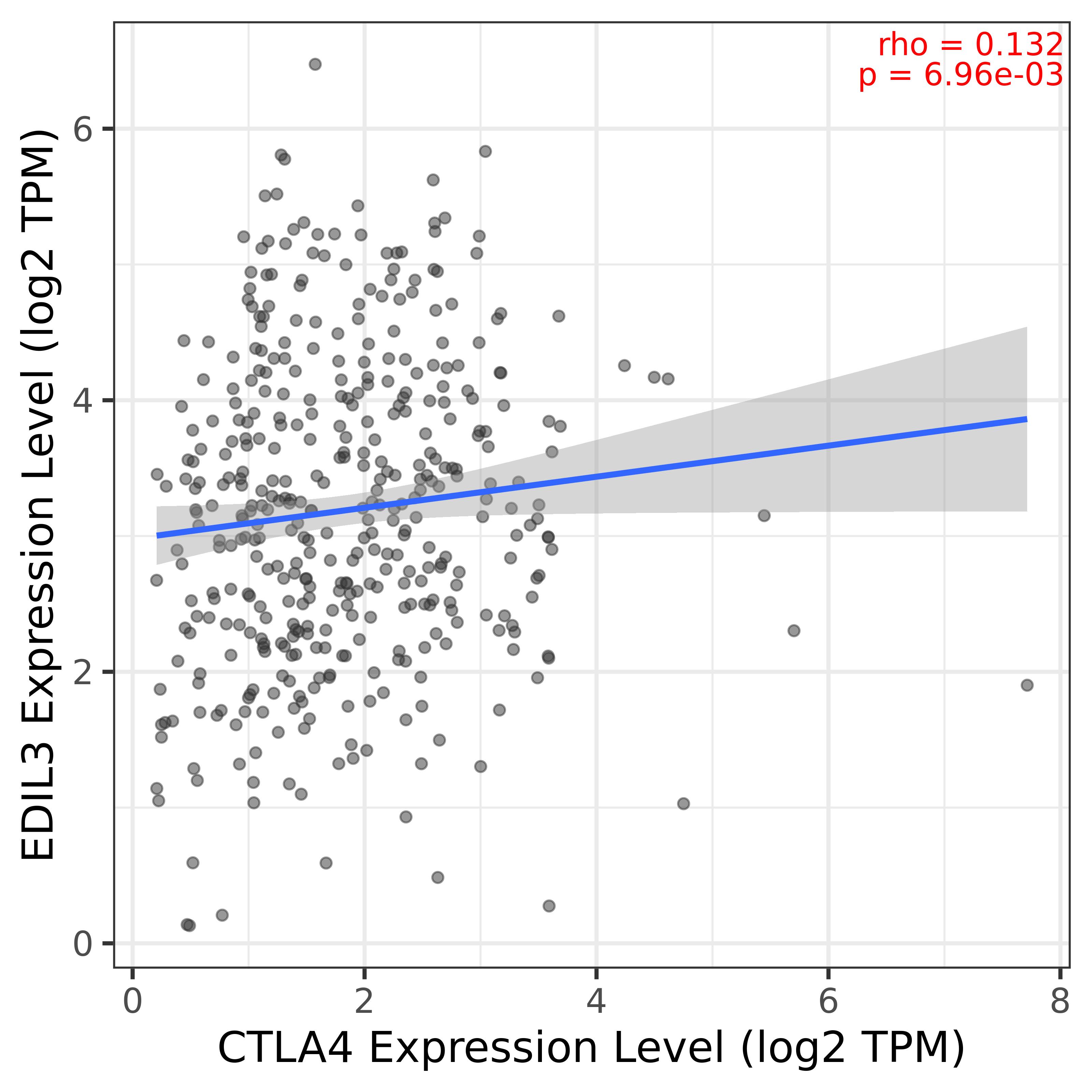

Supplement: Data S2 [file peerj-11-15559-s004.zip › Raw data 2/Raw figure 4-10/Figure 8/Fig 8A CTLA4.jpg]

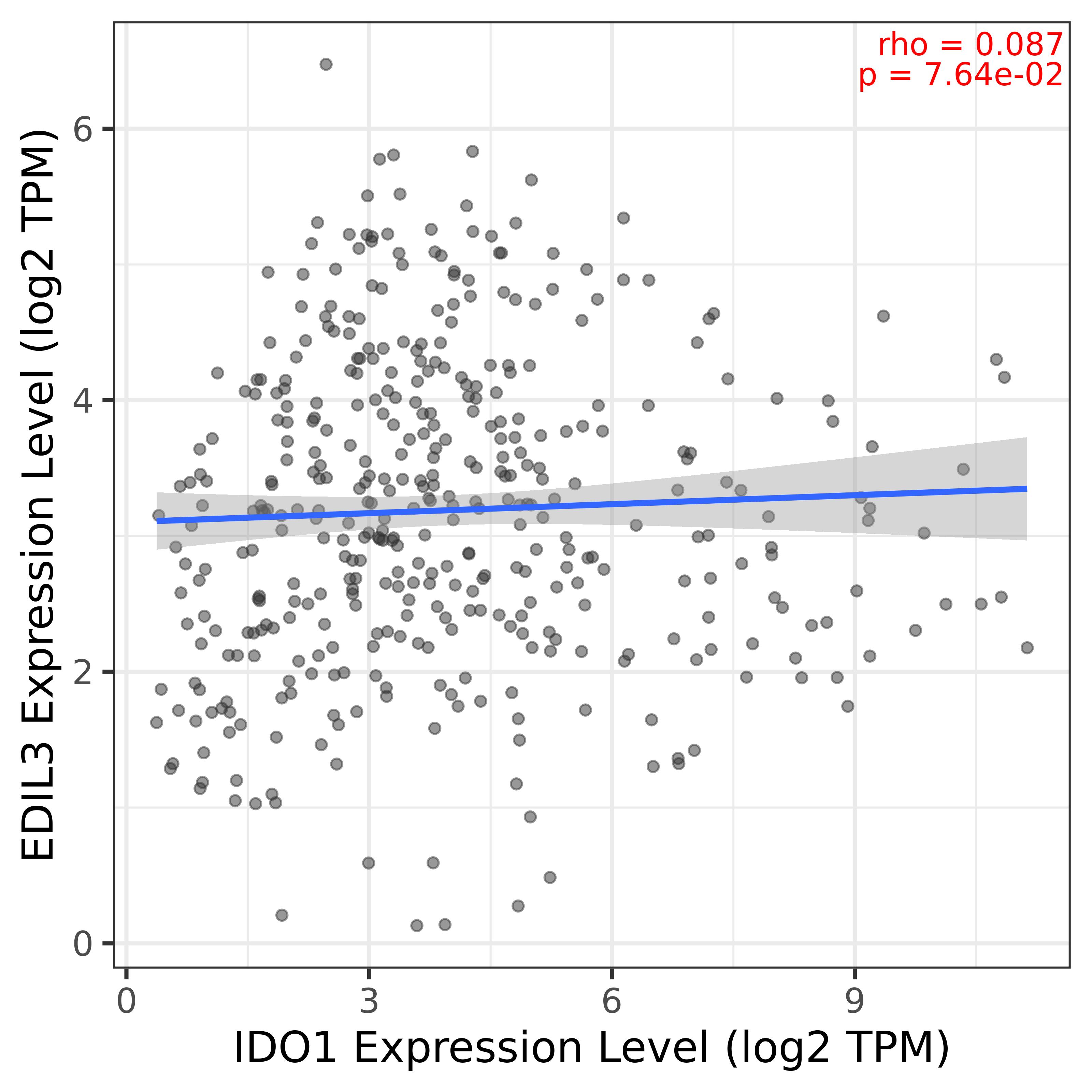

Supplement: Data S2 [file peerj-11-15559-s004.zip › Raw data 2/Raw figure 4-10/Figure 8/Fig 8A IDO1.jpg]

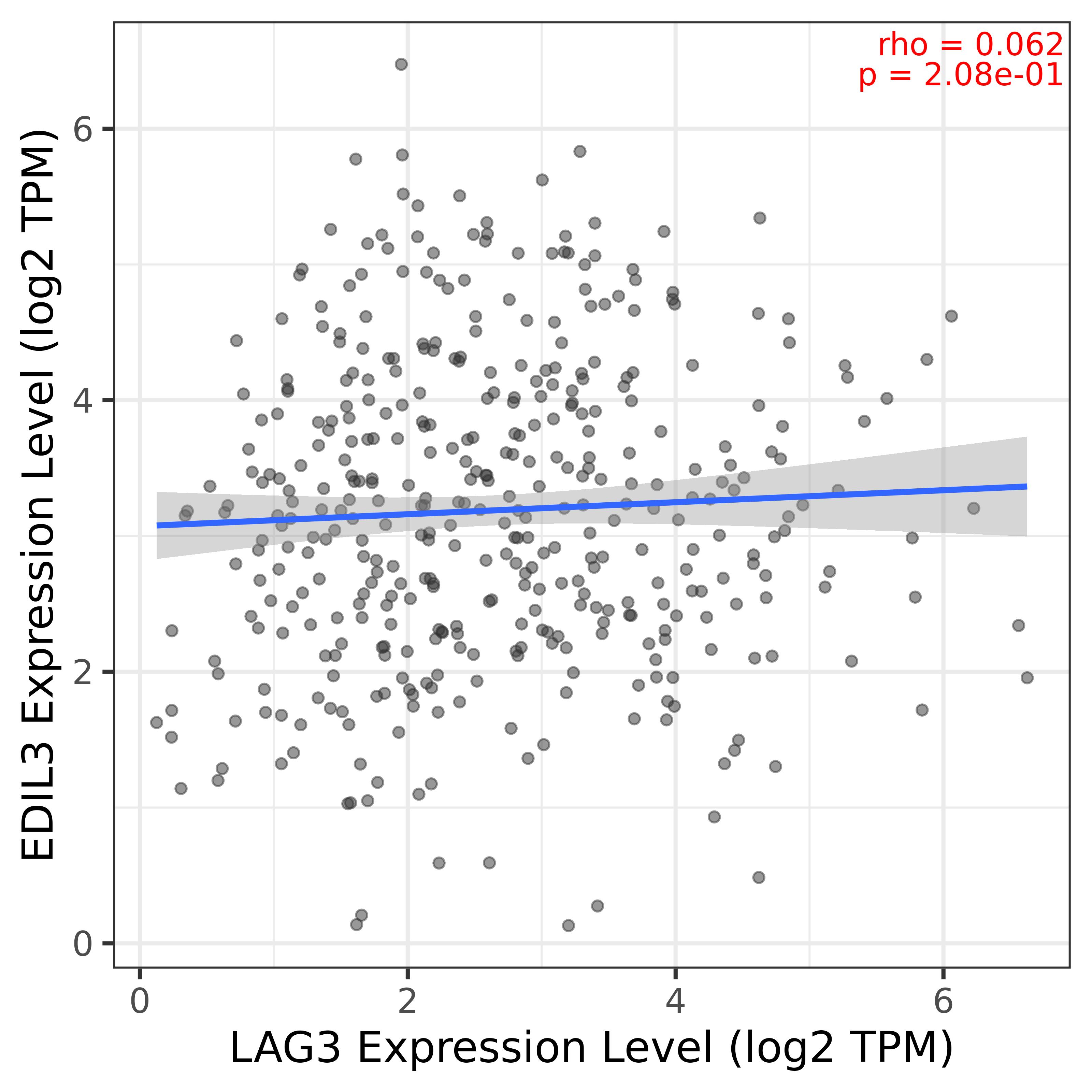

Supplement: Data S2 [file peerj-11-15559-s004.zip › Raw data 2/Raw figure 4-10/Figure 8/Fig 8A LAG3.jpg]

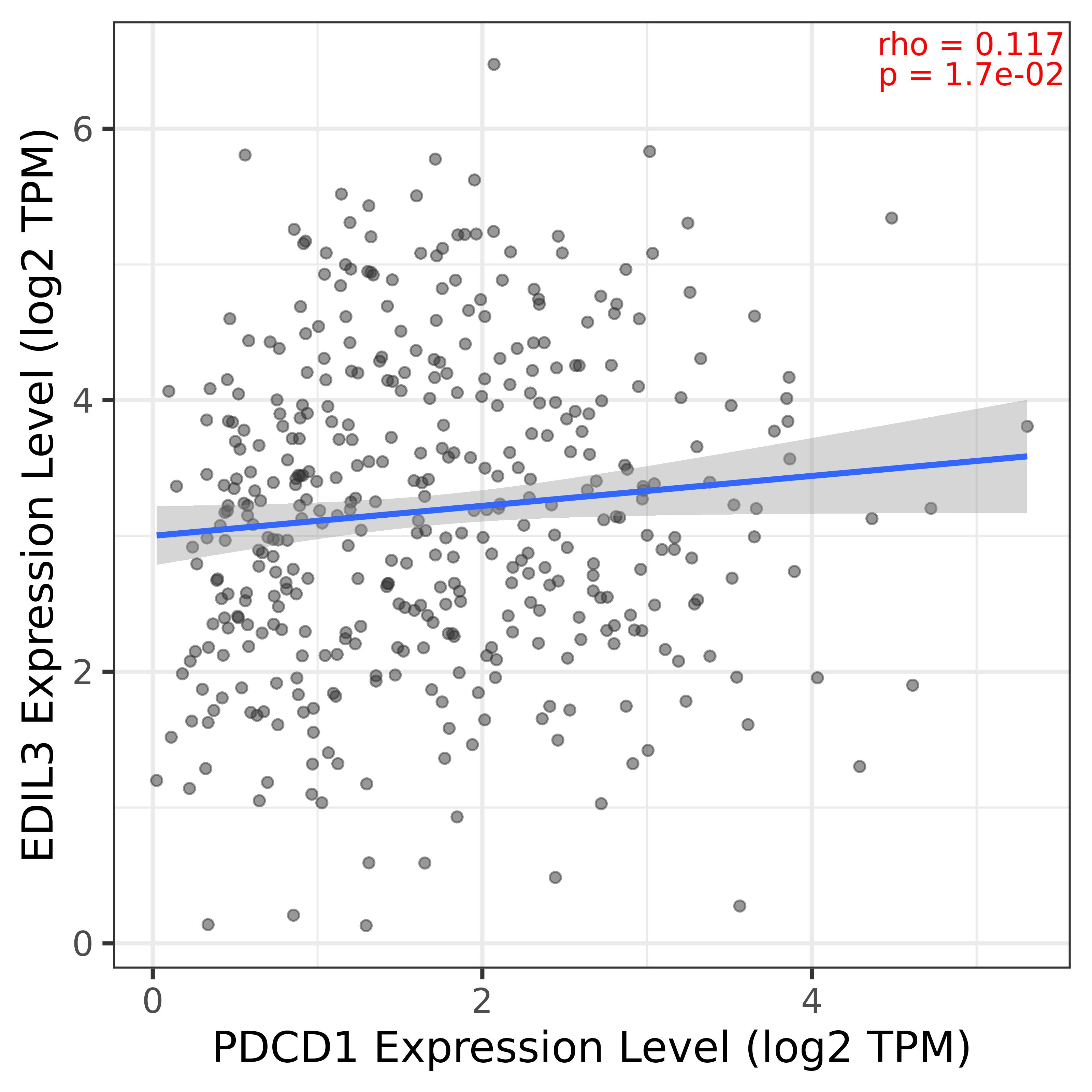

Supplement: Data S2 [file peerj-11-15559-s004.zip › Raw data 2/Raw figure 4-10/Figure 8/Fig 8A PDL1.jpg]

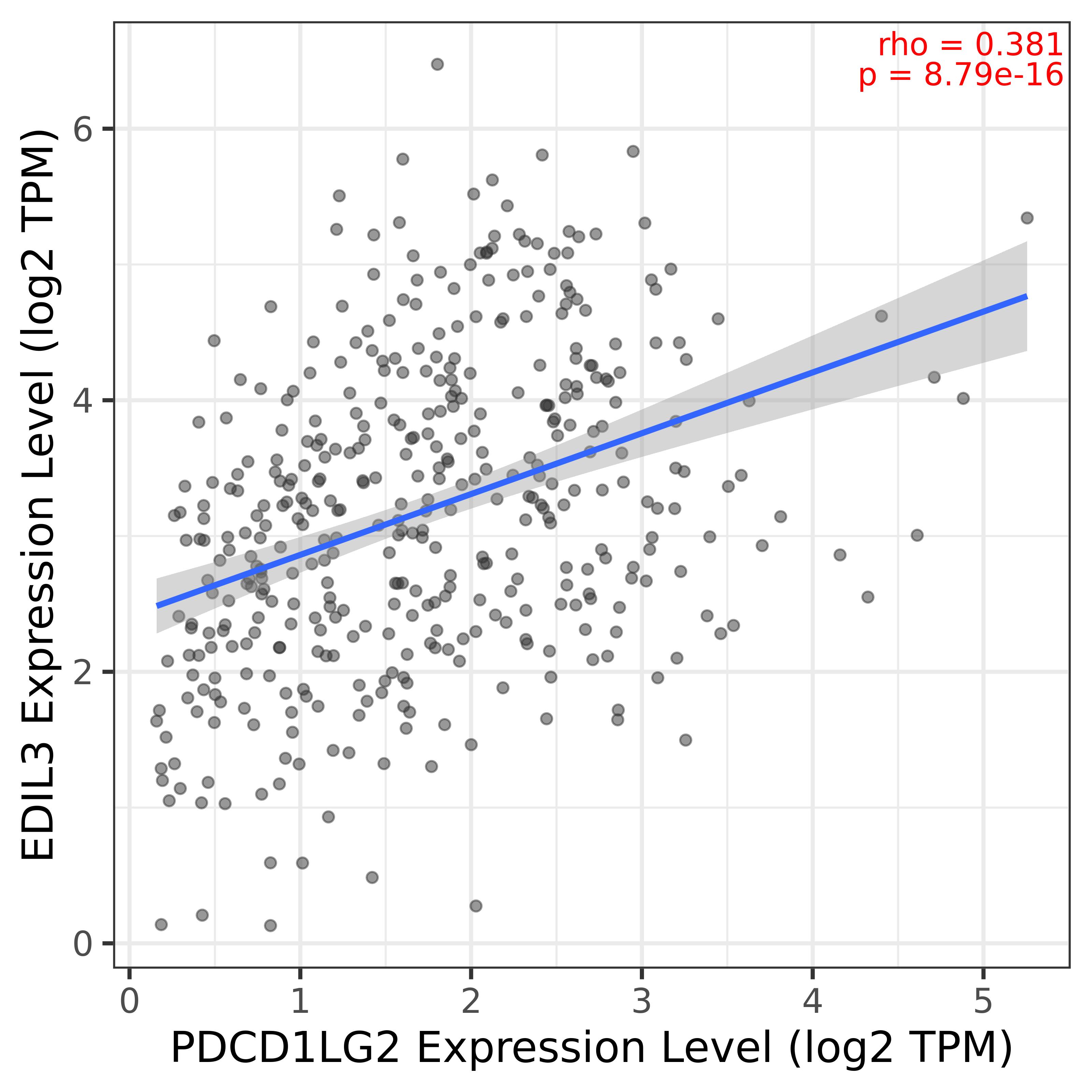

Supplement: Data S2 [file peerj-11-15559-s004.zip › Raw data 2/Raw figure 4-10/Figure 8/Fig 8A PDL2.jpg]

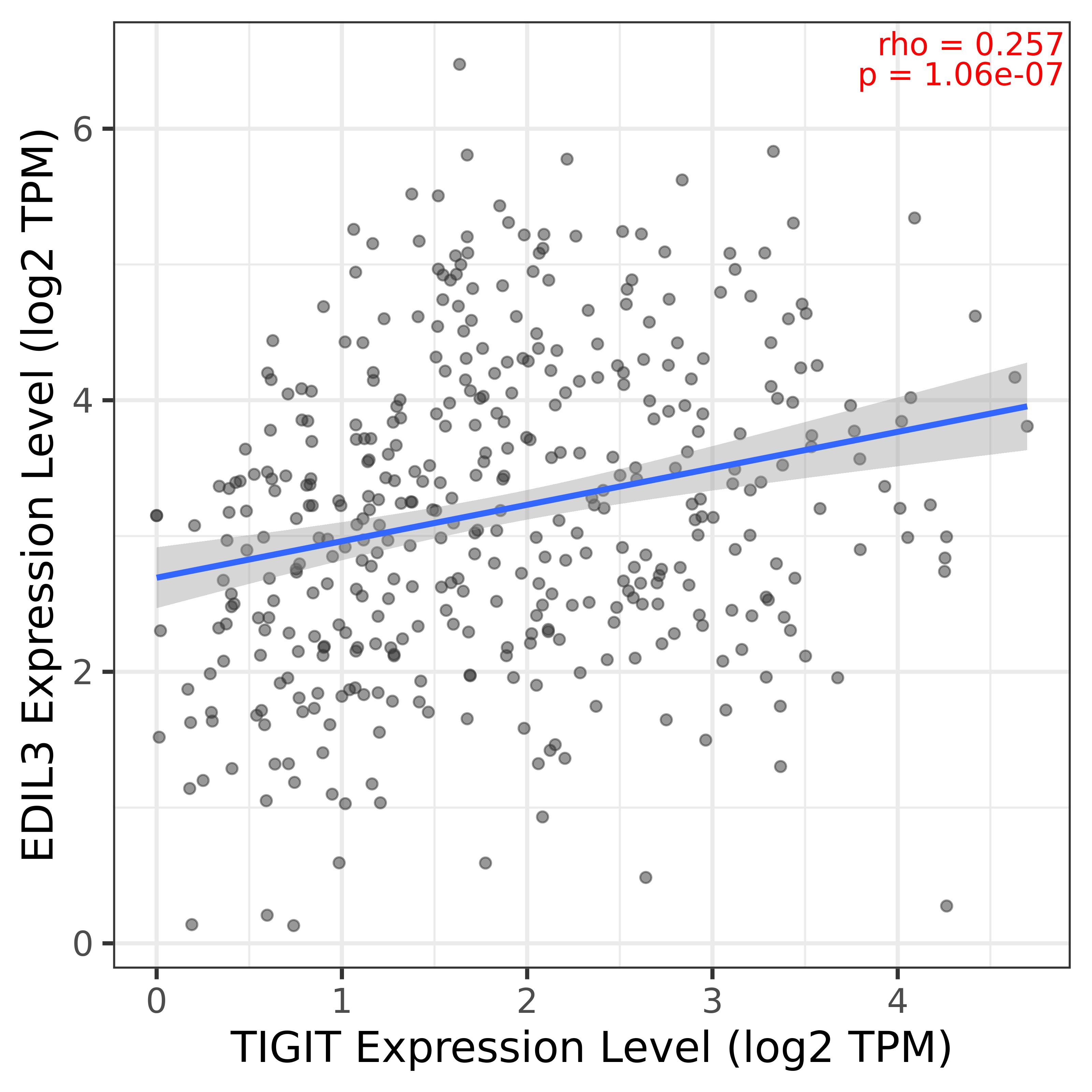

Supplement: Data S2 [file peerj-11-15559-s004.zip › Raw data 2/Raw figure 4-10/Figure 8/Fig 8A TIGIT.jpg]

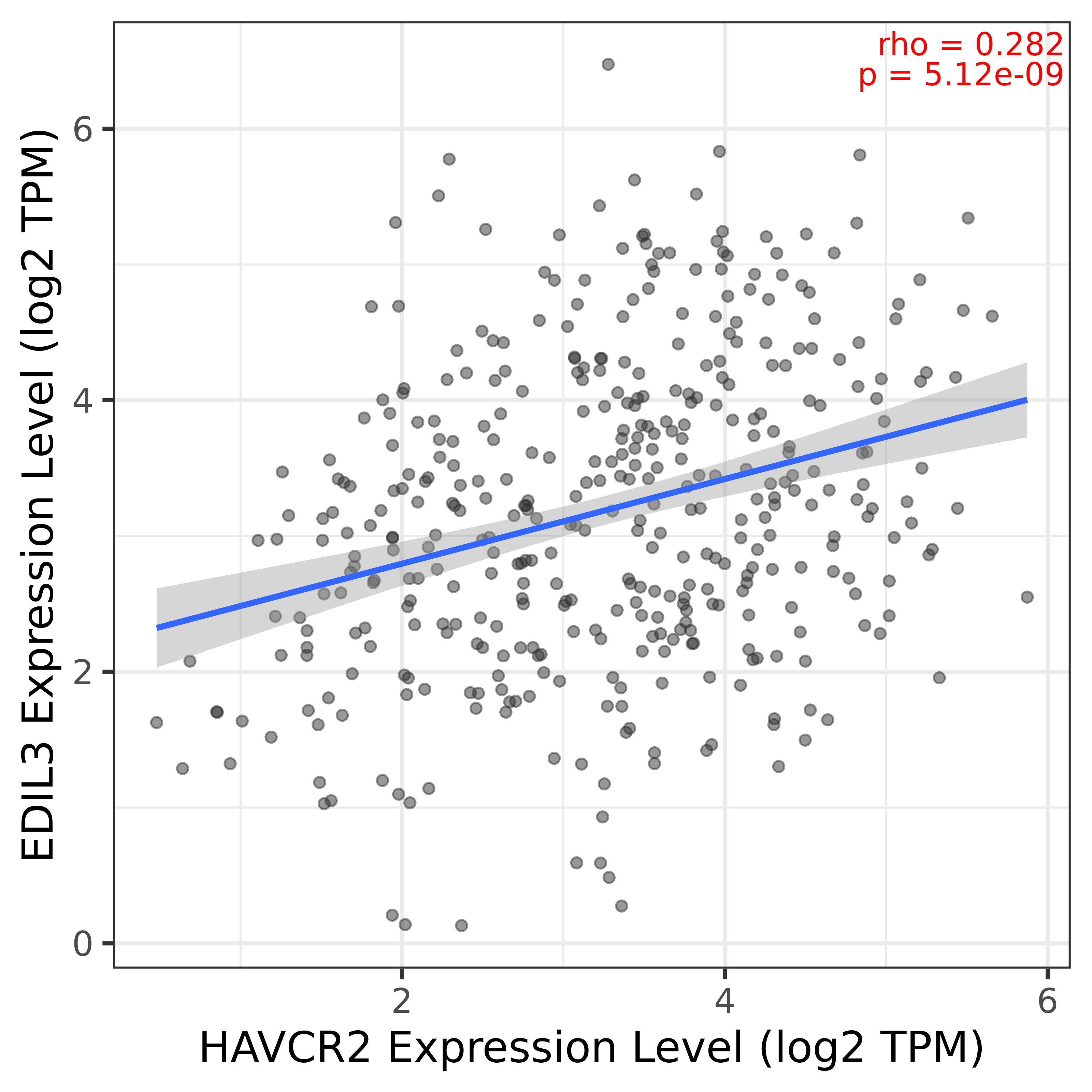

Supplement: Data S2 [file peerj-11-15559-s004.zip › Raw data 2/Raw figure 4-10/Figure 8/Fig 8A TIM3.jpg]

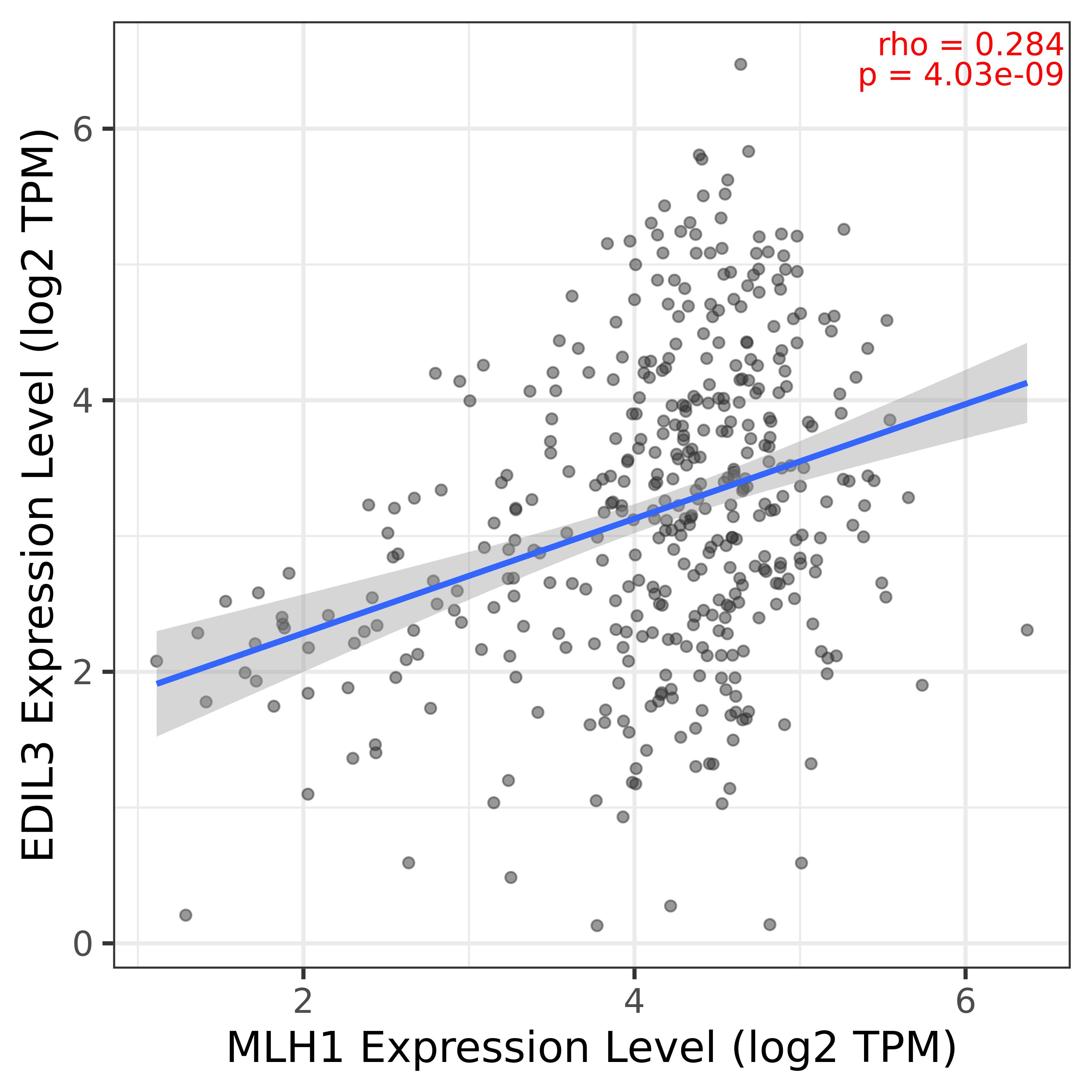

Supplement: Data S2 [file peerj-11-15559-s004.zip › Raw data 2/Raw figure 4-10/Figure 8/Fig 8B MLH1.jpg]

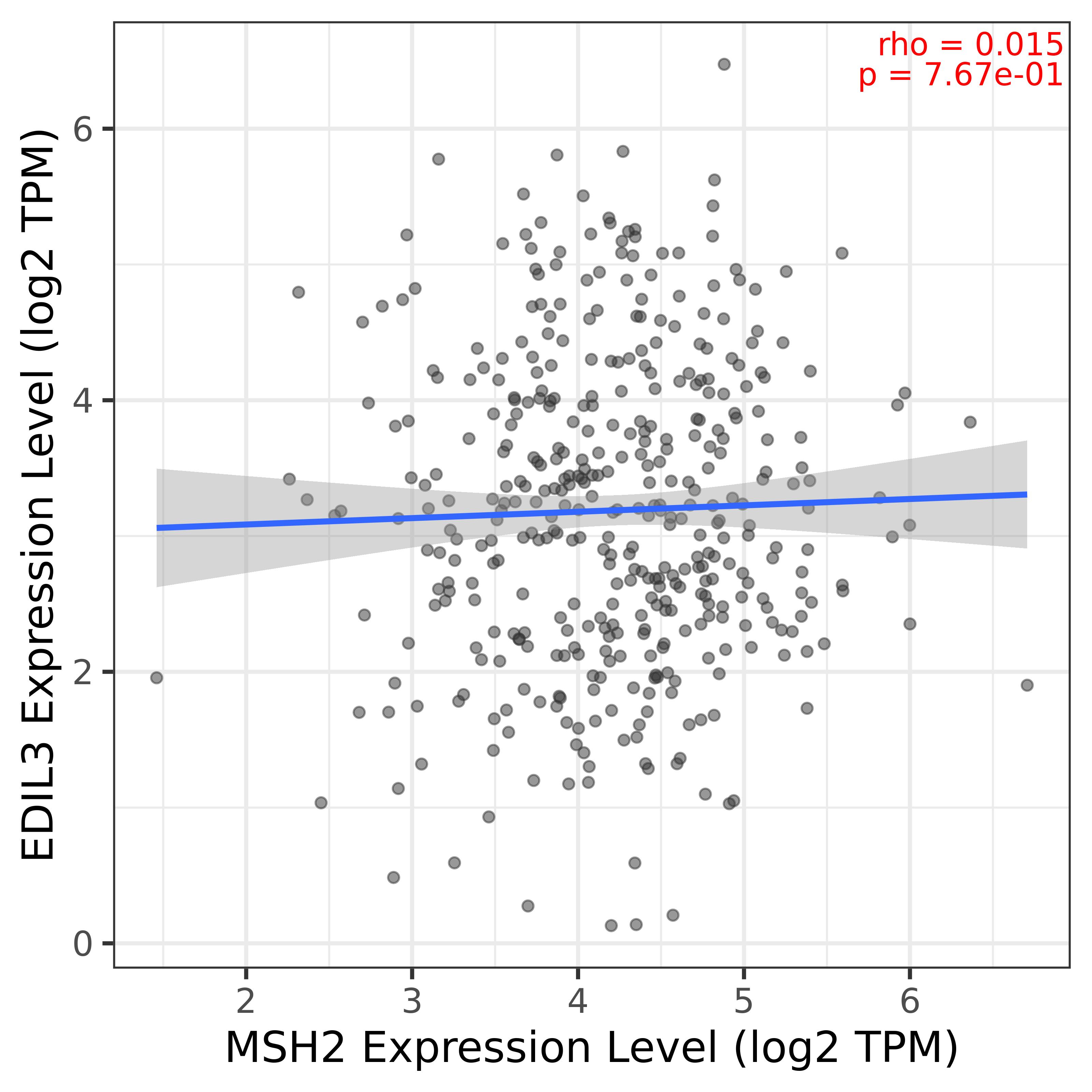

Supplement: Data S2 [file peerj-11-15559-s004.zip › Raw data 2/Raw figure 4-10/Figure 8/Fig 8B MSH2.jpg]

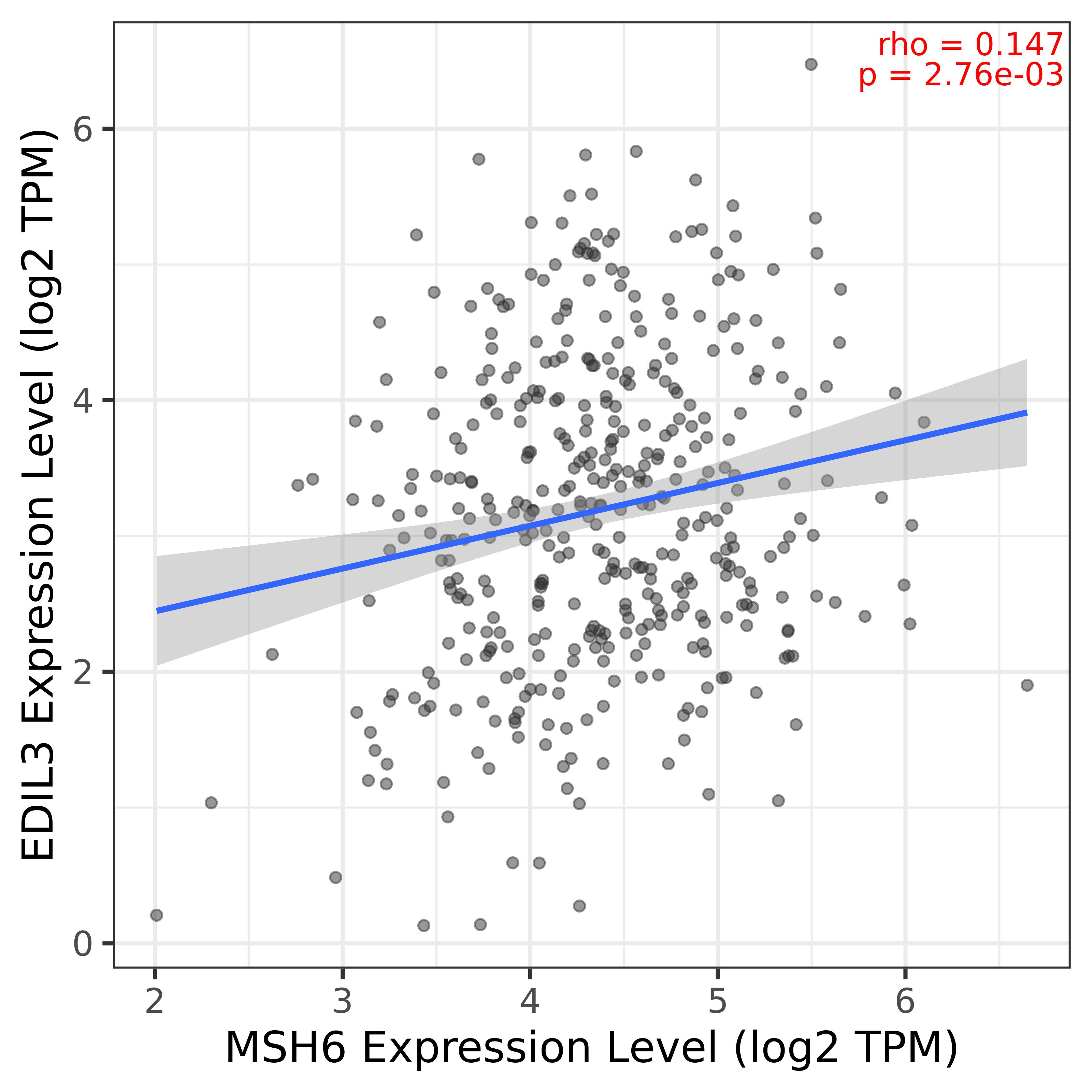

Supplement: Data S2 [file peerj-11-15559-s004.zip › Raw data 2/Raw figure 4-10/Figure 8/Fig 8B MSH6.jpg]

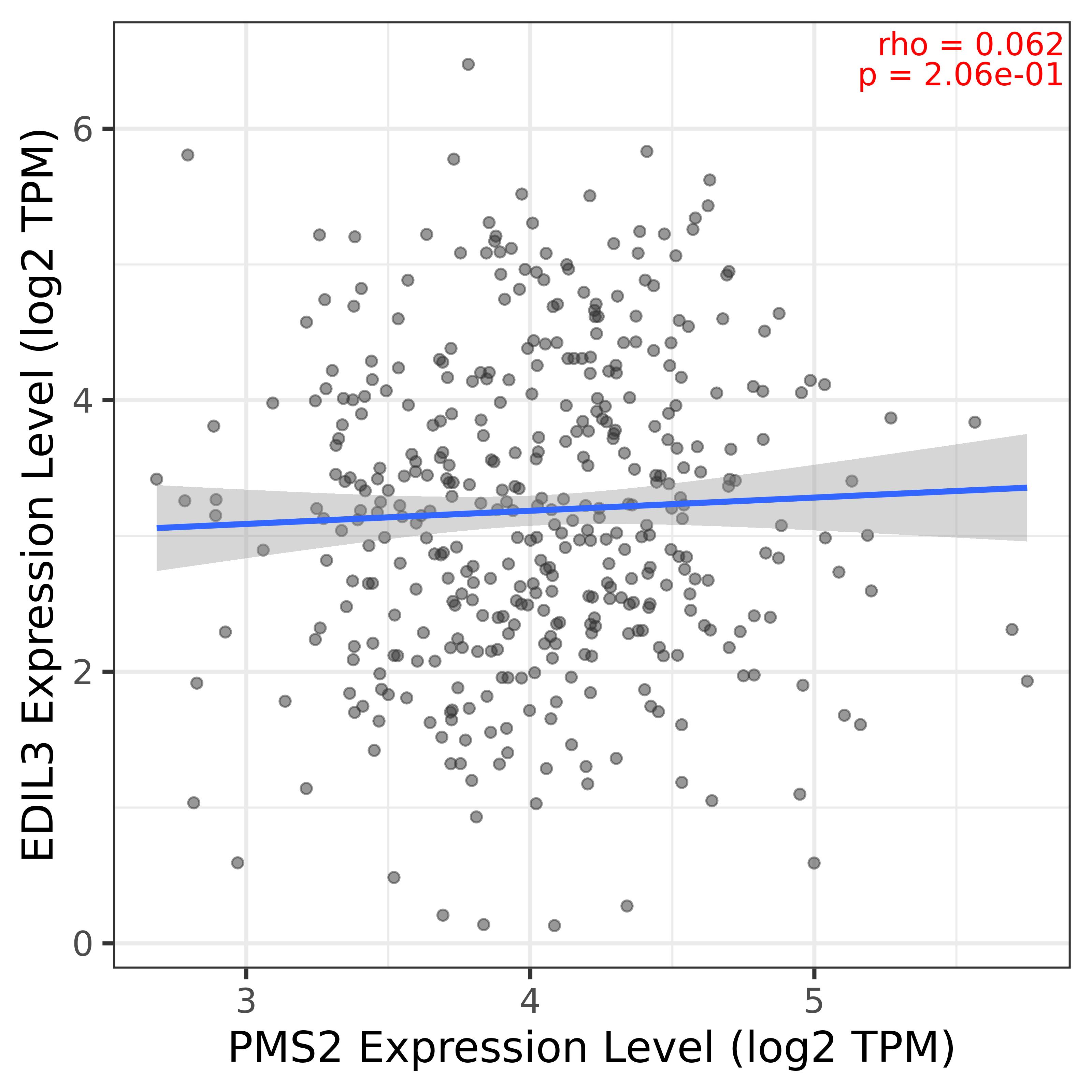

Supplement: Data S2 [file peerj-11-15559-s004.zip › Raw data 2/Raw figure 4-10/Figure 8/Fig 8B PMS2.jpg]

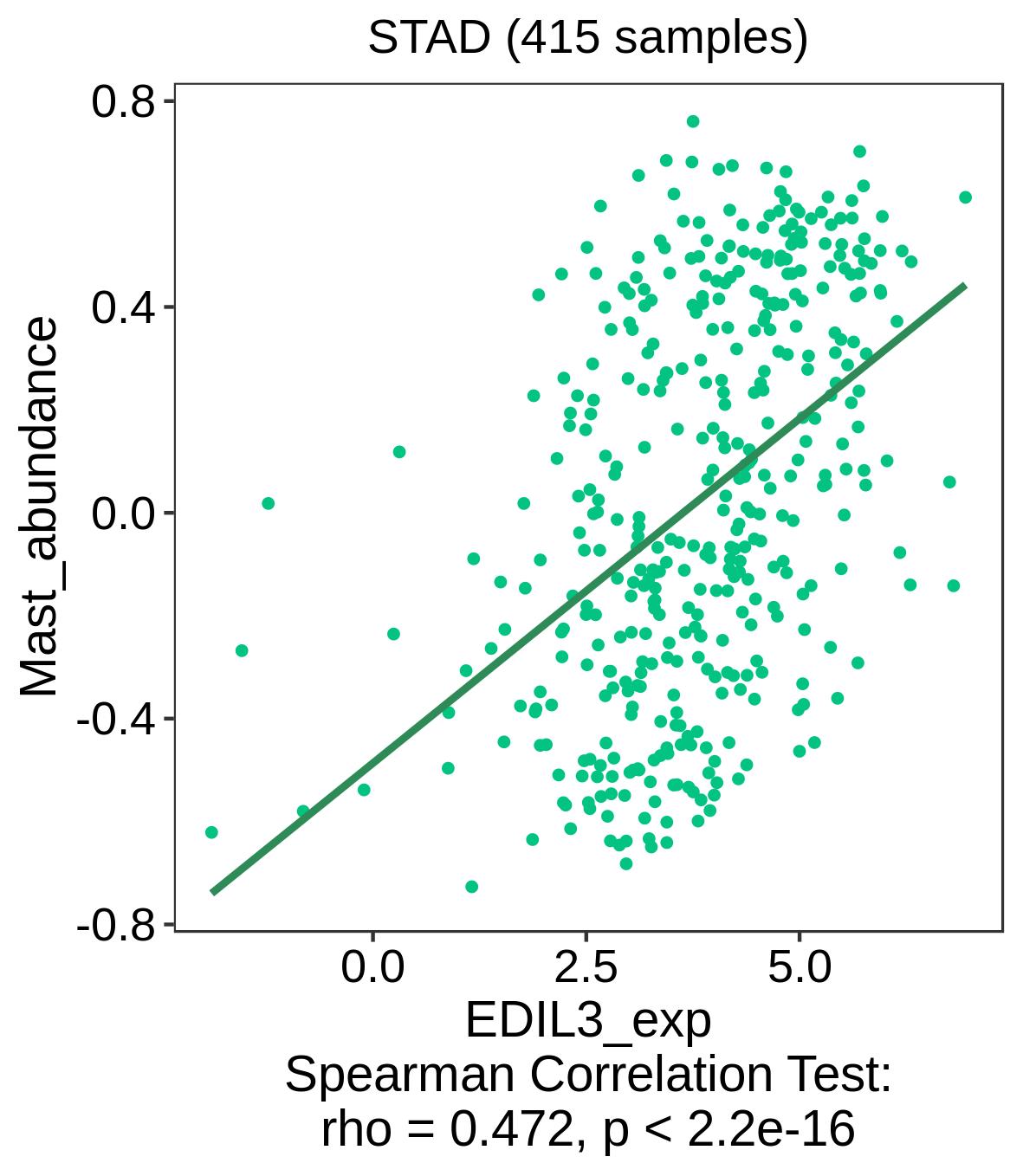

Supplement: Data S2 [file peerj-11-15559-s004.zip › Raw data 2/Raw figure 4-10/Figure 9/Fig 9A lymphocyte/1 Mast lymphocyte.jpg]

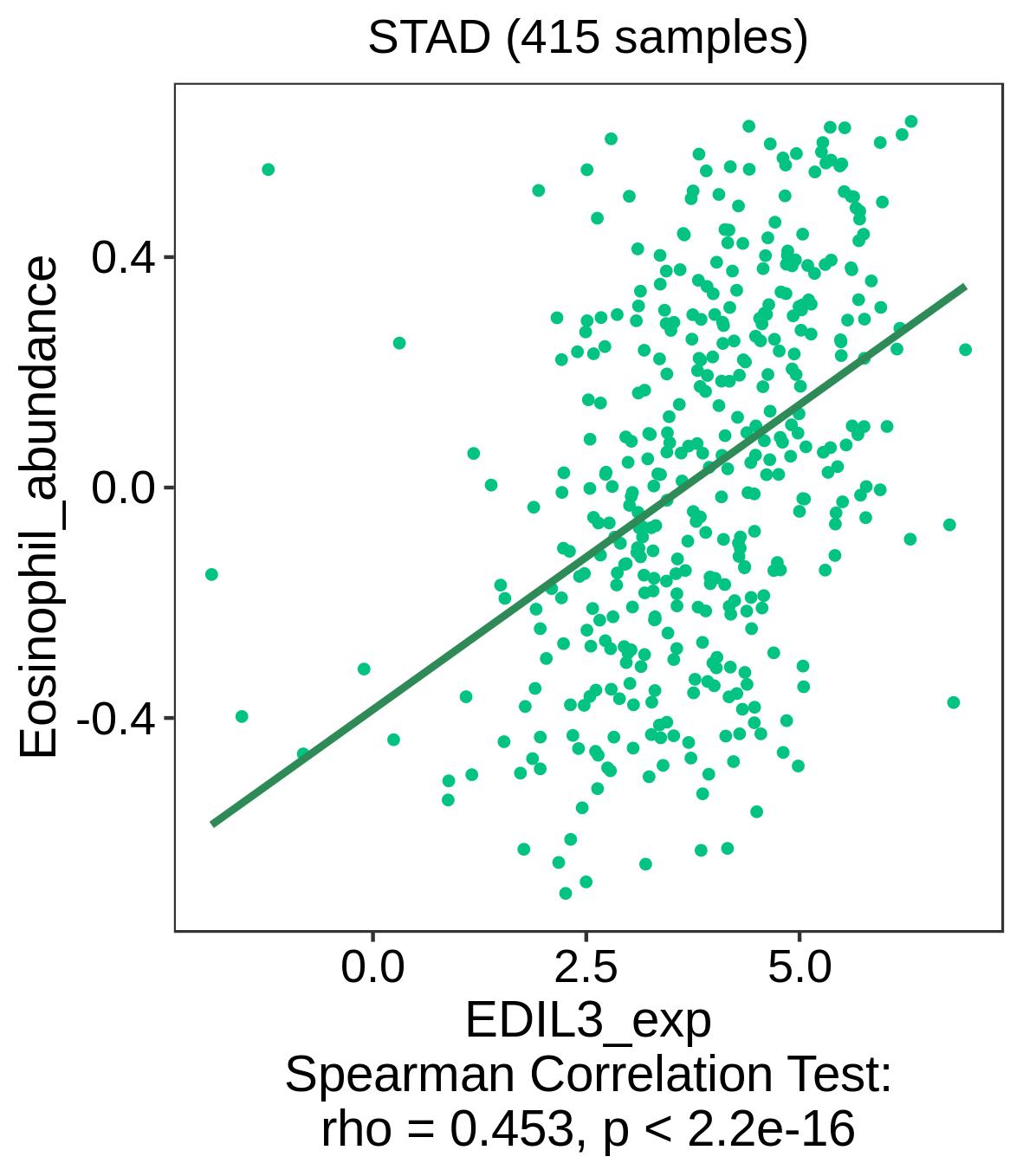

Supplement: Data S2 [file peerj-11-15559-s004.zip › Raw data 2/Raw figure 4-10/Figure 9/Fig 9A lymphocyte/2 Eosinophil lymphocyte.jpg]

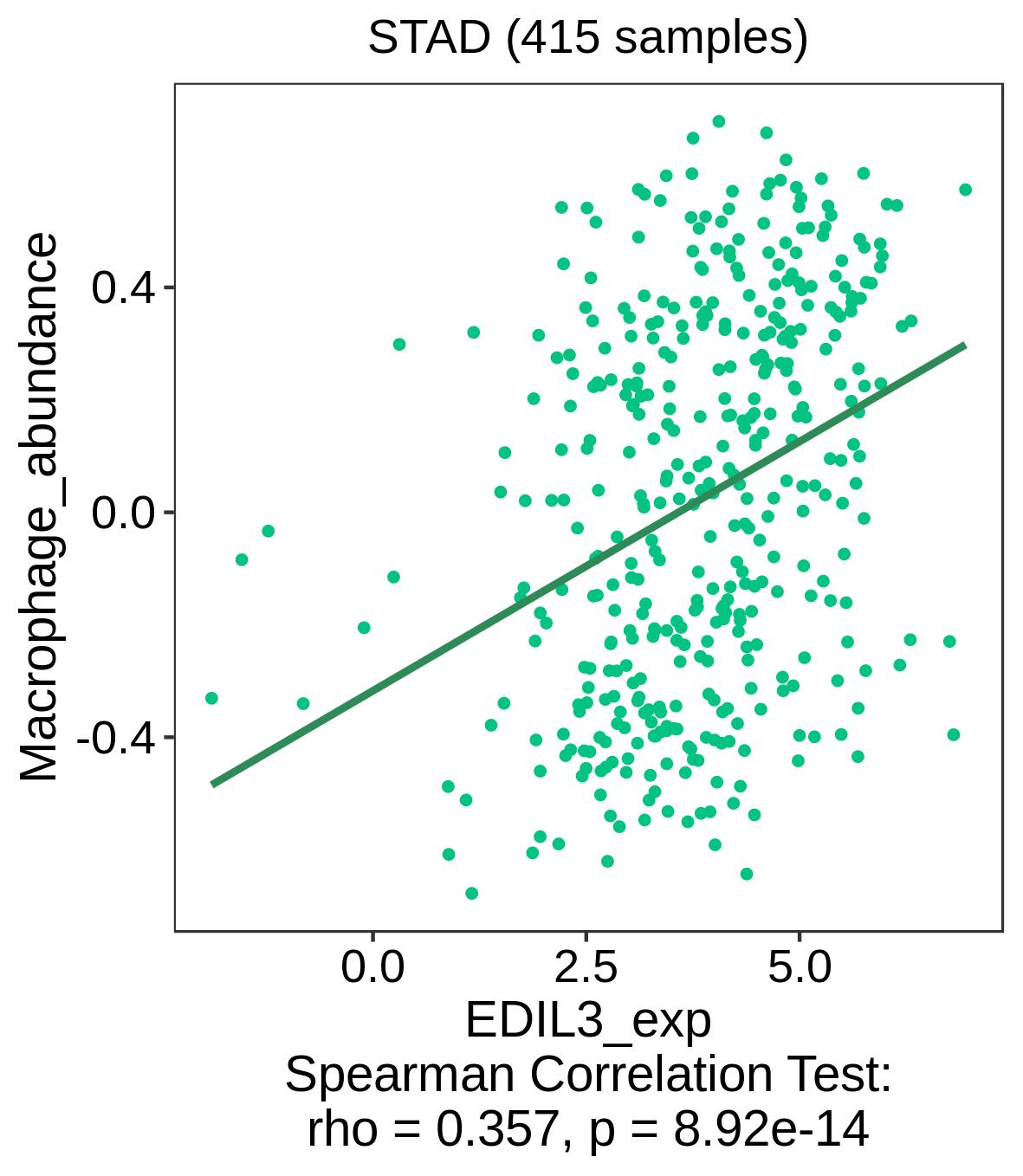

Supplement: Data S2 [file peerj-11-15559-s004.zip › Raw data 2/Raw figure 4-10/Figure 9/Fig 9A lymphocyte/3 Macrophage lymphocyte.jpg]

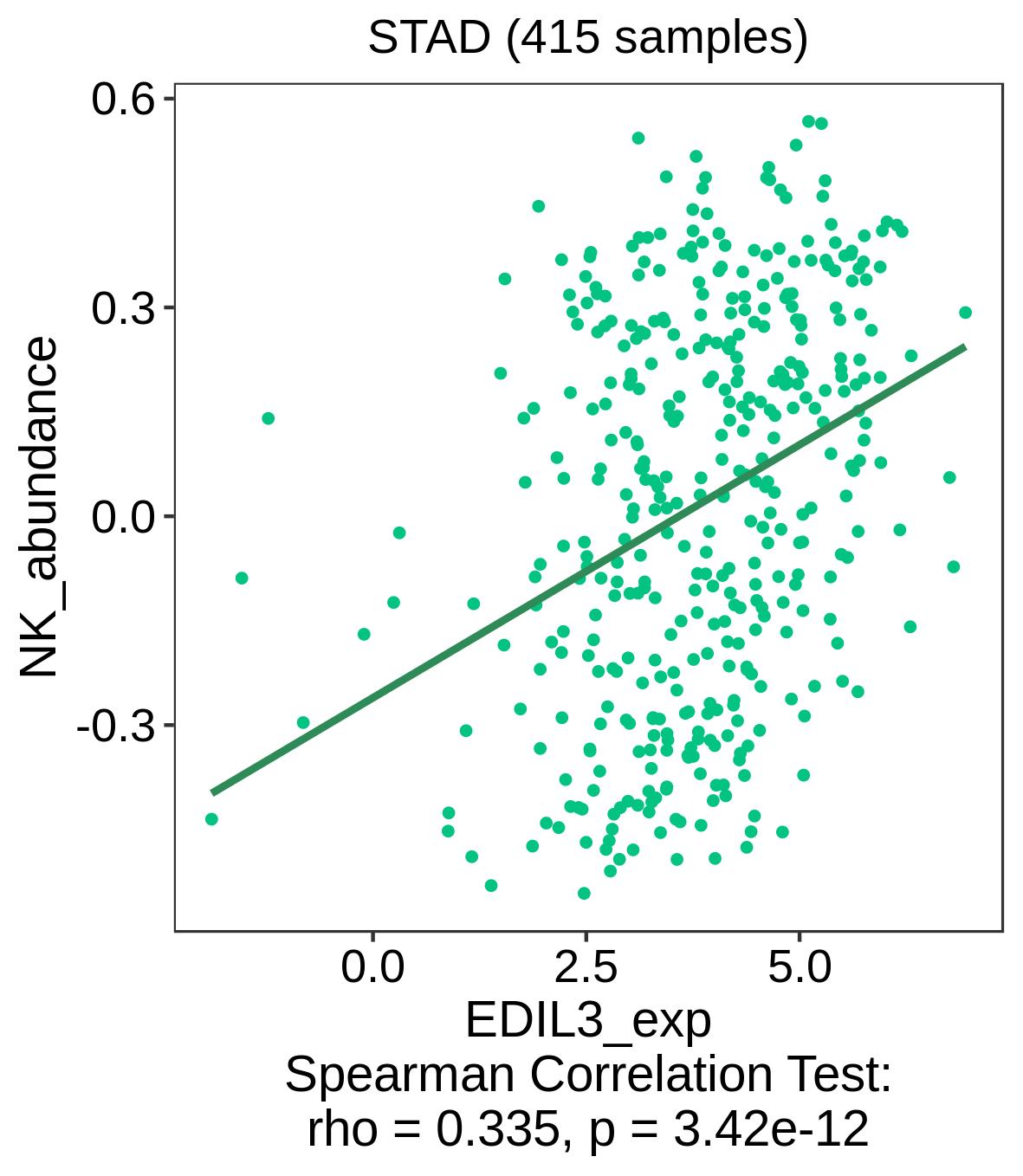

Supplement: Data S2 [file peerj-11-15559-s004.zip › Raw data 2/Raw figure 4-10/Figure 9/Fig 9A lymphocyte/4 NK lymphocyte.jpg]

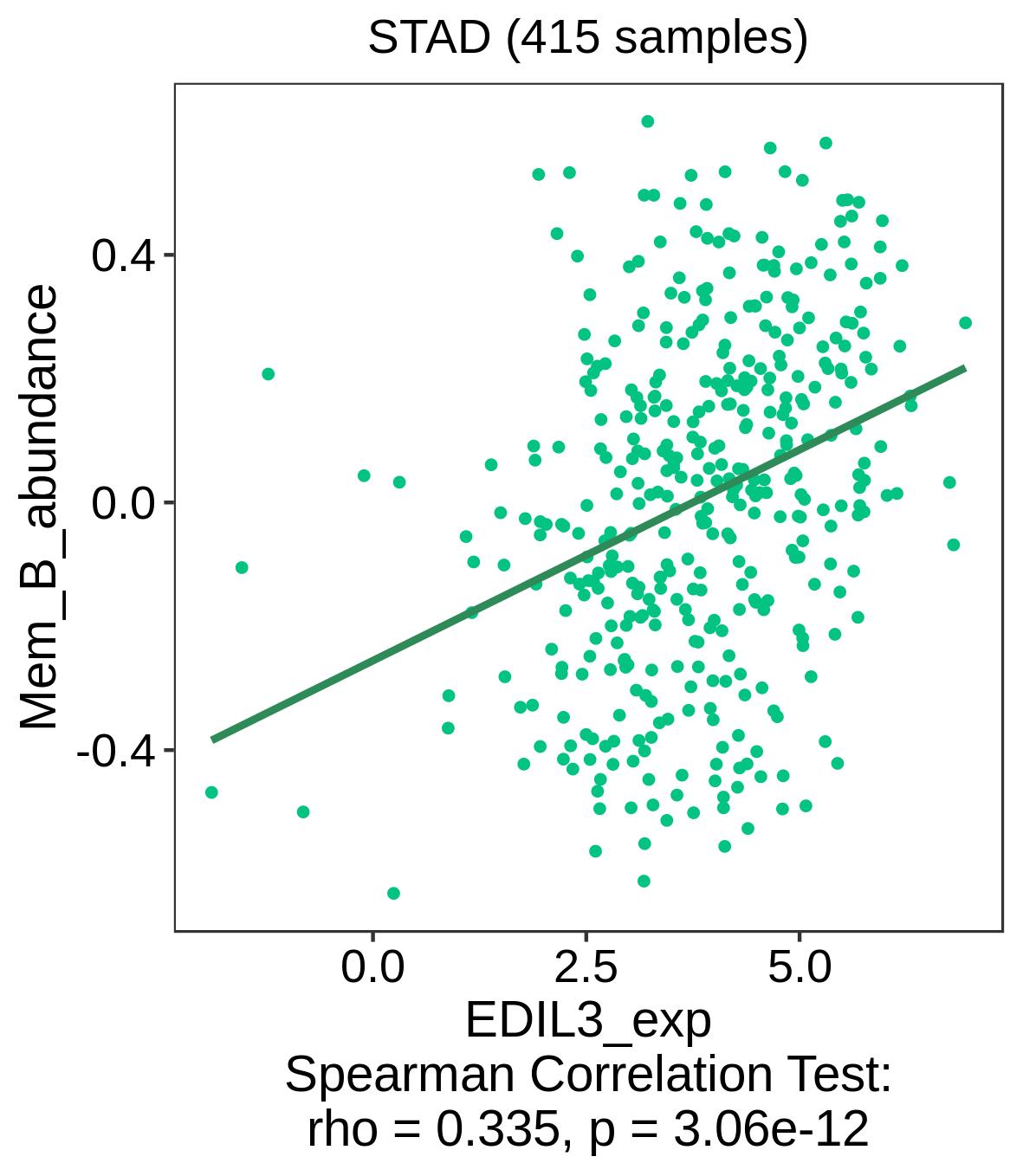

Supplement: Data S2 [file peerj-11-15559-s004.zip › Raw data 2/Raw figure 4-10/Figure 9/Fig 9A lymphocyte/5 Mem_B lymphocyte.jpg]

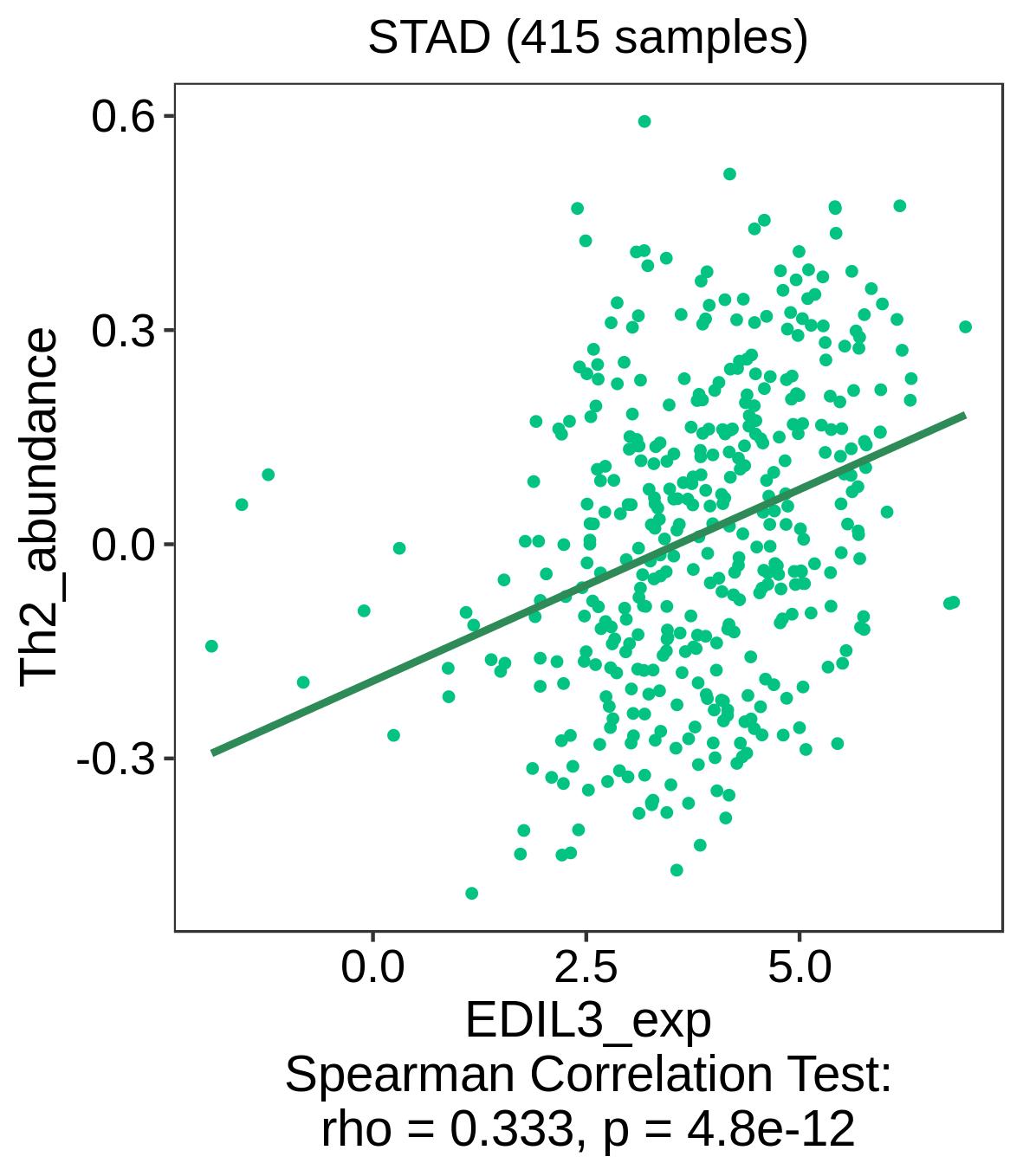

Supplement: Data S2 [file peerj-11-15559-s004.zip › Raw data 2/Raw figure 4-10/Figure 9/Fig 9A lymphocyte/6 Th2 lymphocyte.jpg]

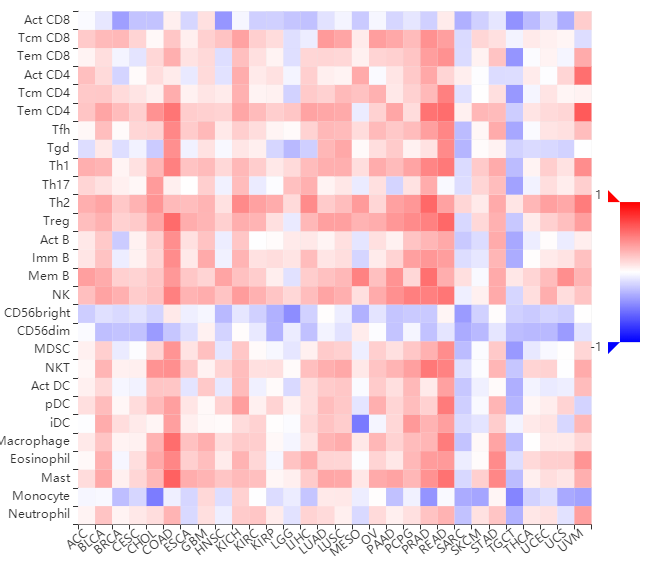

Supplement: Data S2 [file peerj-11-15559-s004.zip › Raw data 2/Raw figure 4-10/Figure 9/Fig 9A lymphocyte/TISIDB lymphocytes.png]

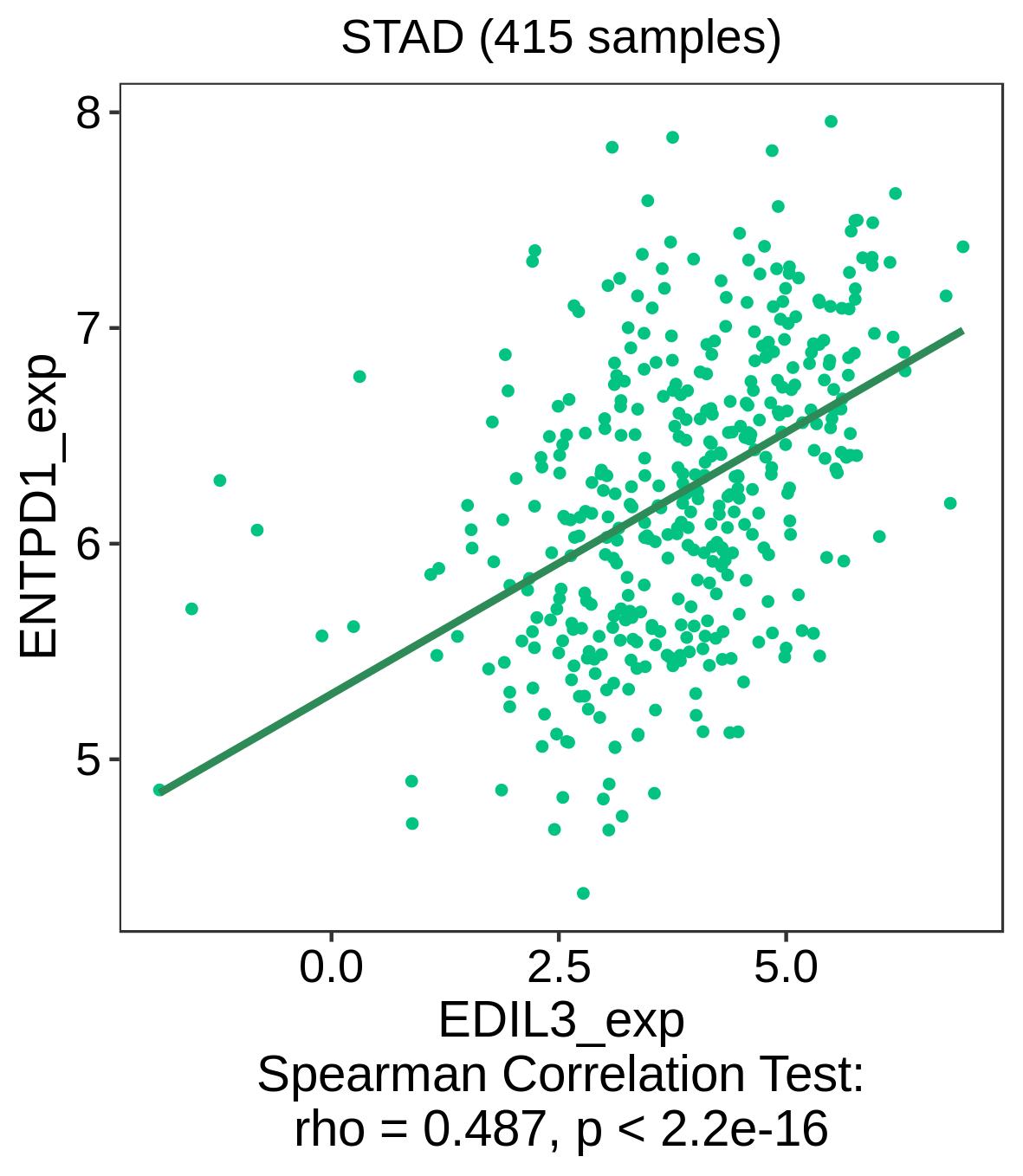

Supplement: Data S2 [file peerj-11-15559-s004.zip › Raw data 2/Raw figure 4-10/Figure 9/Fig 9B Immunostimulator/1 Immunostimulator_ENTPD1.jpg]

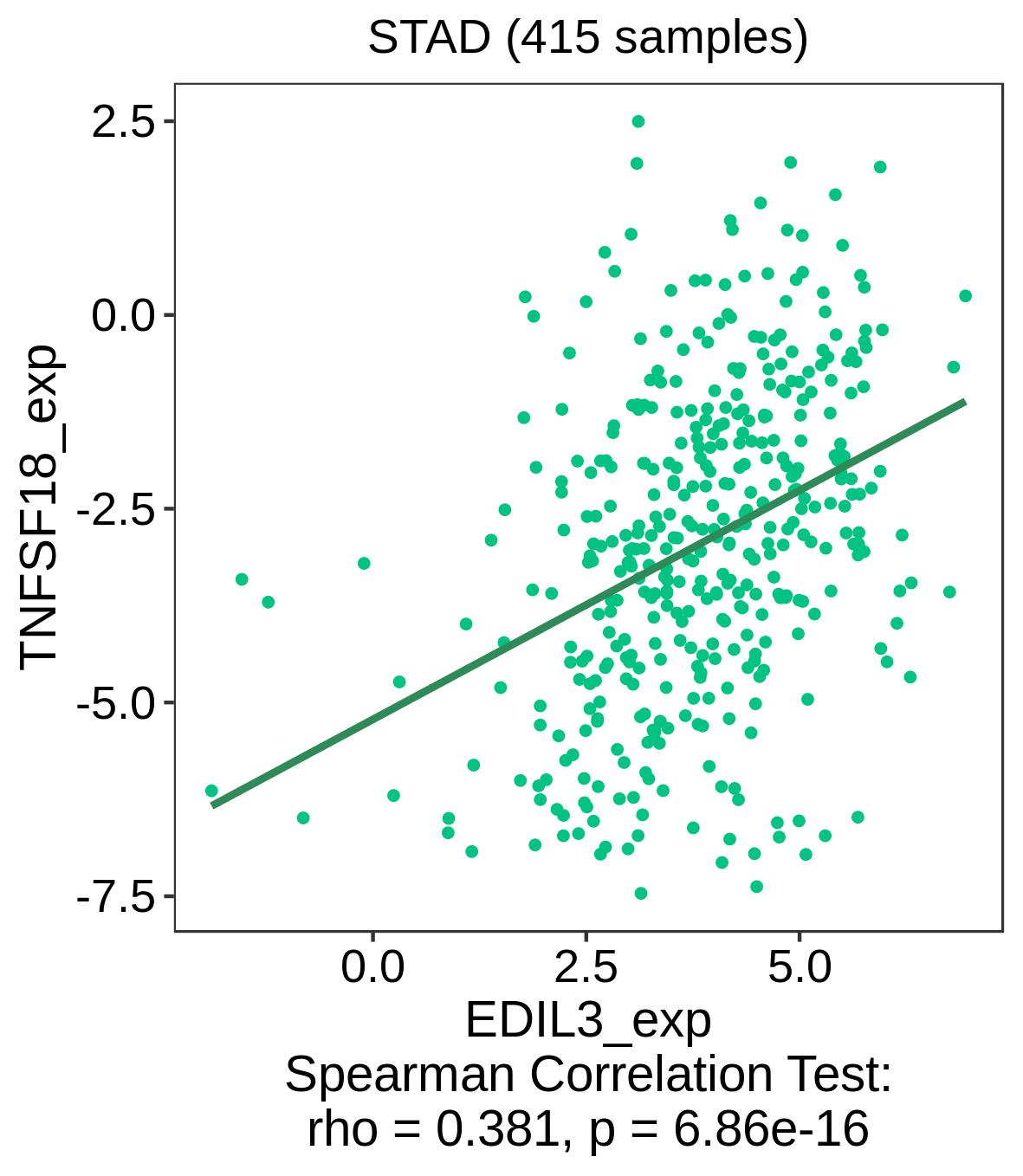

Supplement: Data S2 [file peerj-11-15559-s004.zip › Raw data 2/Raw figure 4-10/Figure 9/Fig 9B Immunostimulator/2 Immunostimulator_TNFSF18.jpg]

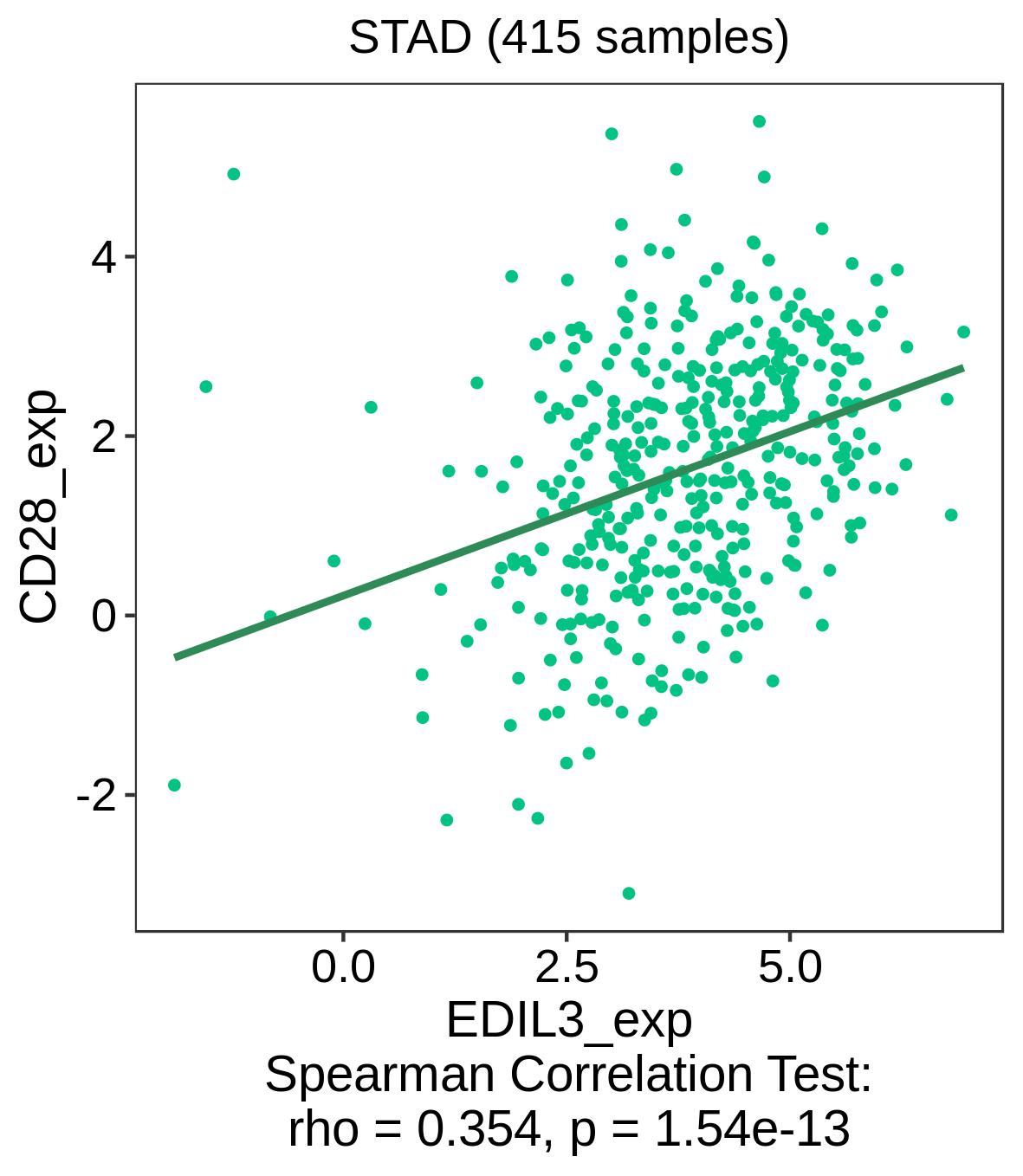

Supplement: Data S2 [file peerj-11-15559-s004.zip › Raw data 2/Raw figure 4-10/Figure 9/Fig 9B Immunostimulator/3 Immunostimulator_CD28.jpg]

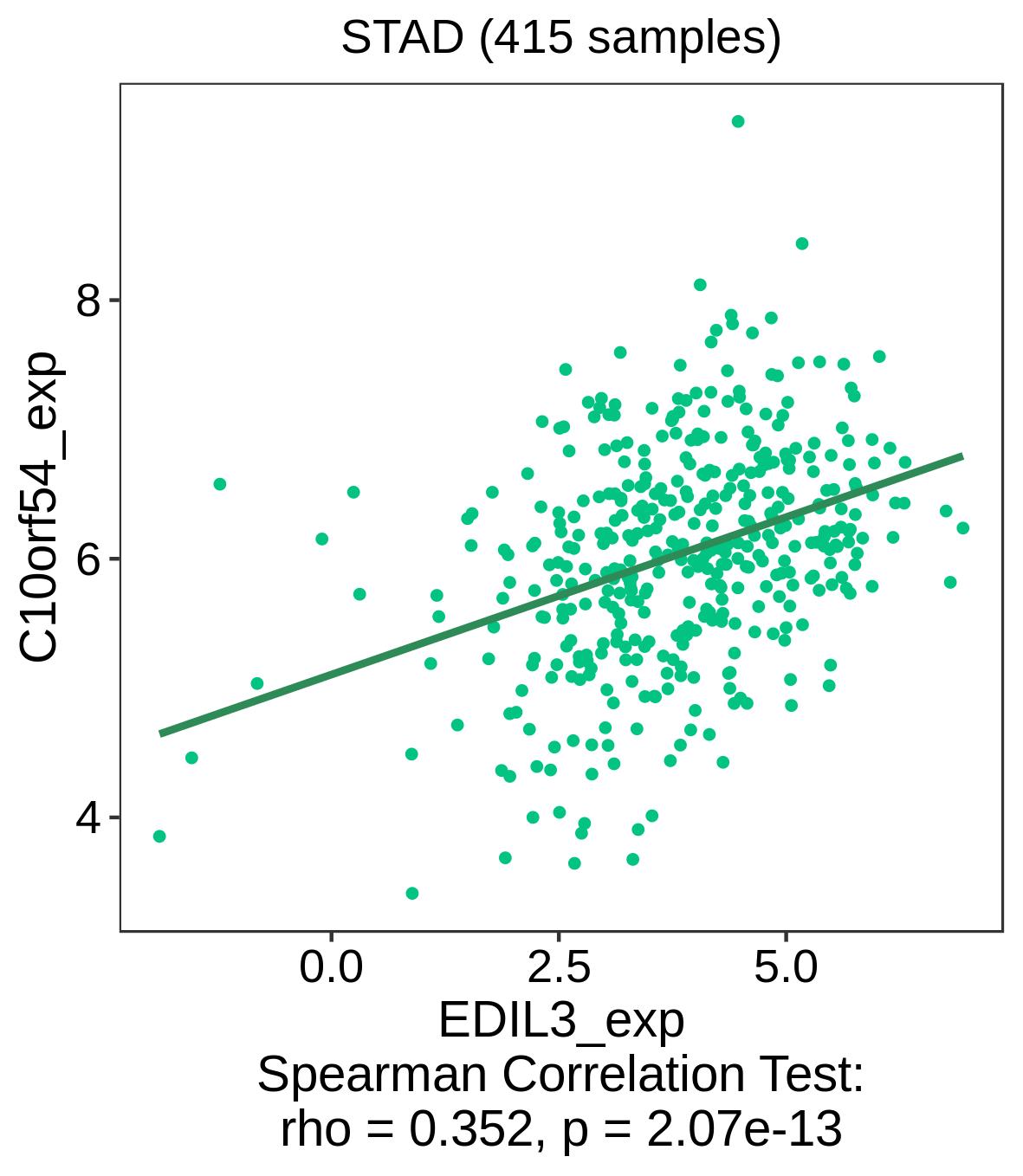

Supplement: Data S2 [file peerj-11-15559-s004.zip › Raw data 2/Raw figure 4-10/Figure 9/Fig 9B Immunostimulator/4 Immunostimulator_C10orf54.jpg]

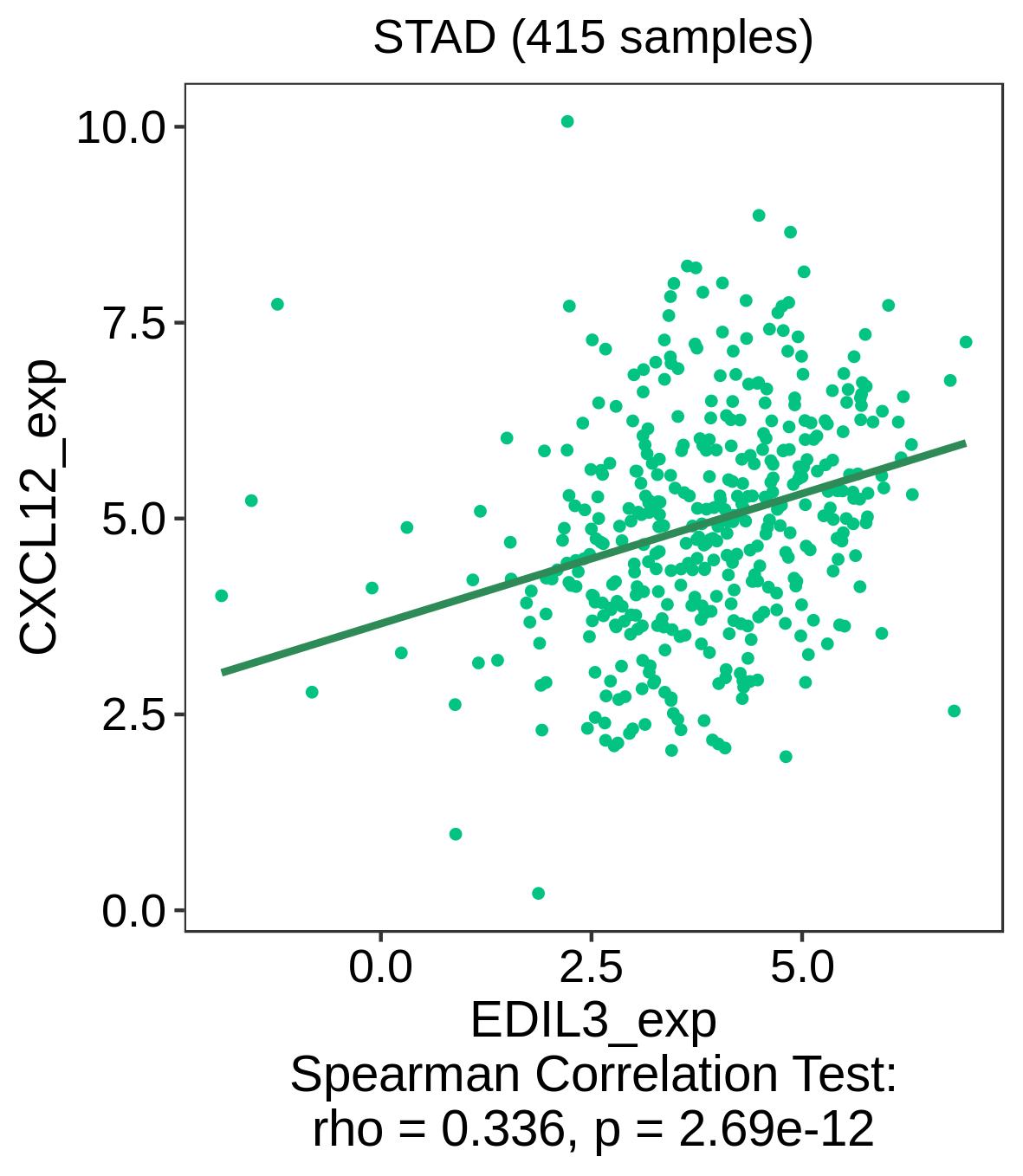

Supplement: Data S2 [file peerj-11-15559-s004.zip › Raw data 2/Raw figure 4-10/Figure 9/Fig 9B Immunostimulator/5 Immunostimulator_CXCL12.jpg]

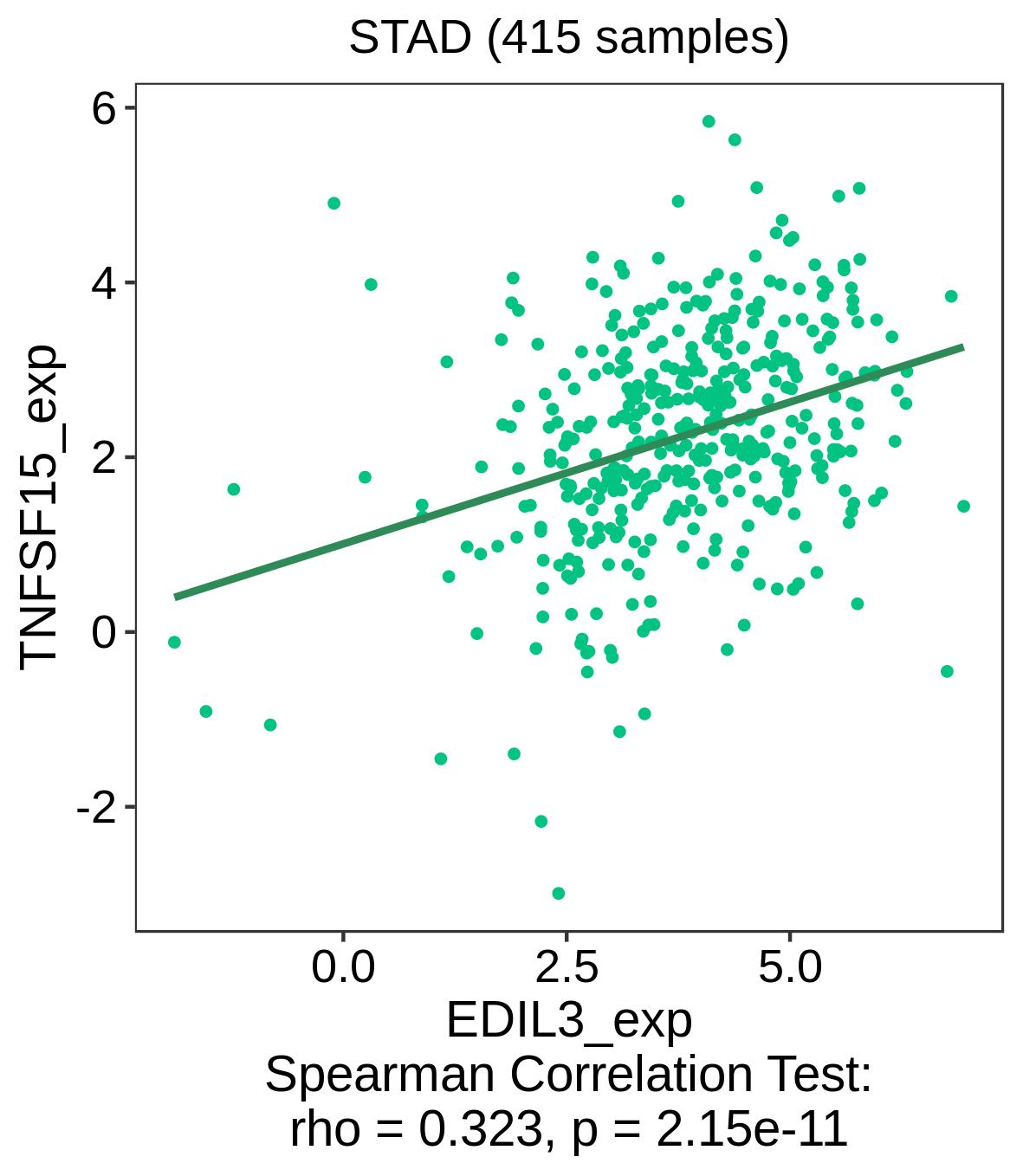

Supplement: Data S2 [file peerj-11-15559-s004.zip › Raw data 2/Raw figure 4-10/Figure 9/Fig 9B Immunostimulator/6 Immunostimulator_TNFSF15.jpg]

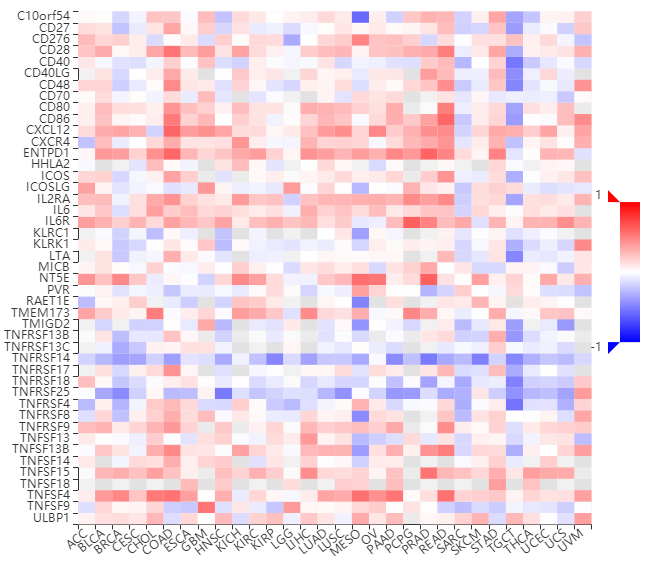

Supplement: Data S2 [file peerj-11-15559-s004.zip › Raw data 2/Raw figure 4-10/Figure 9/Fig 9B Immunostimulator/Immunostimulator.png]

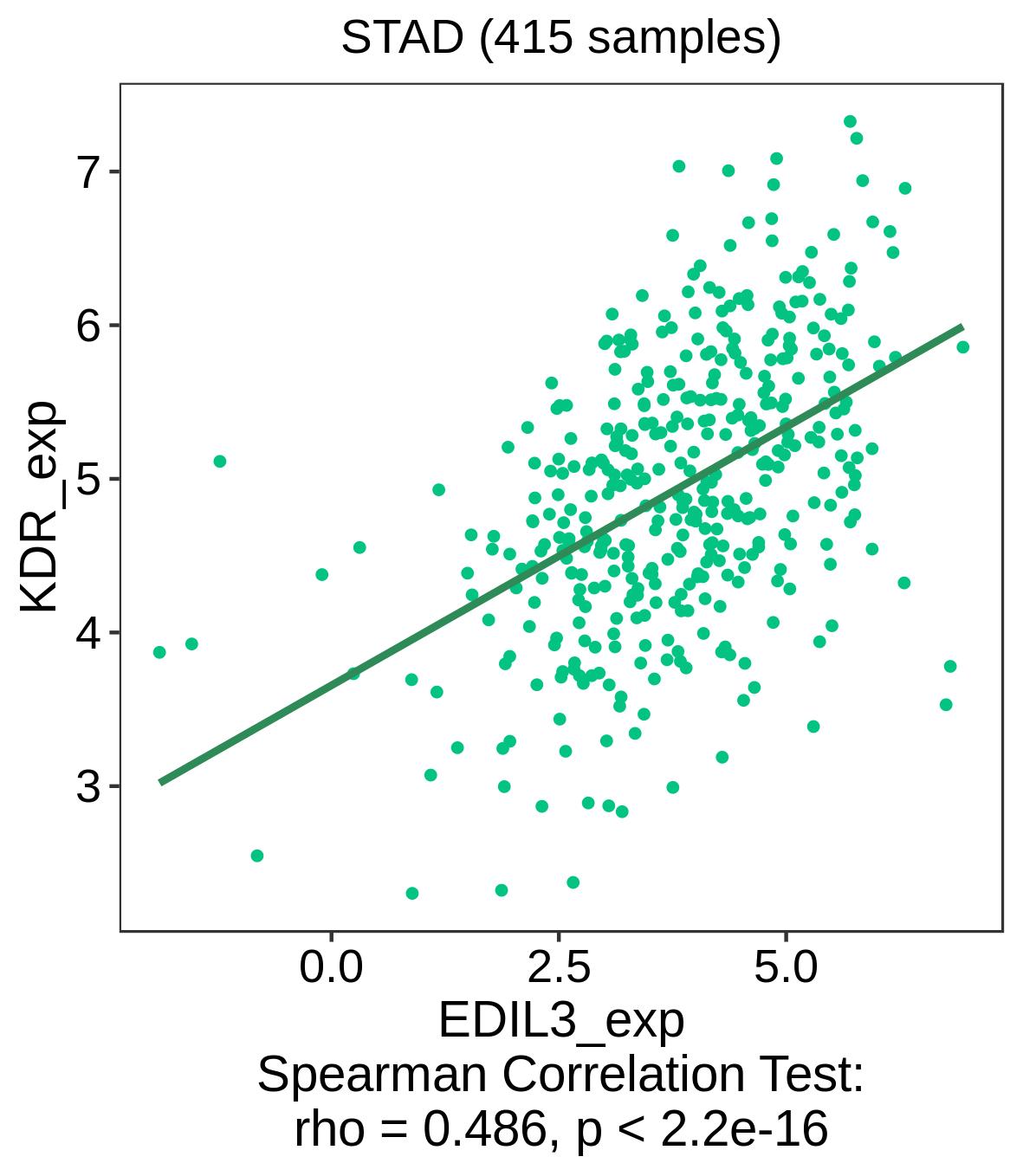

Supplement: Data S2 [file peerj-11-15559-s004.zip › Raw data 2/Raw figure 4-10/Figure 9/Fig 9C Immunoinhibitor/1 Immunoinhibitor_KDR.jpg]

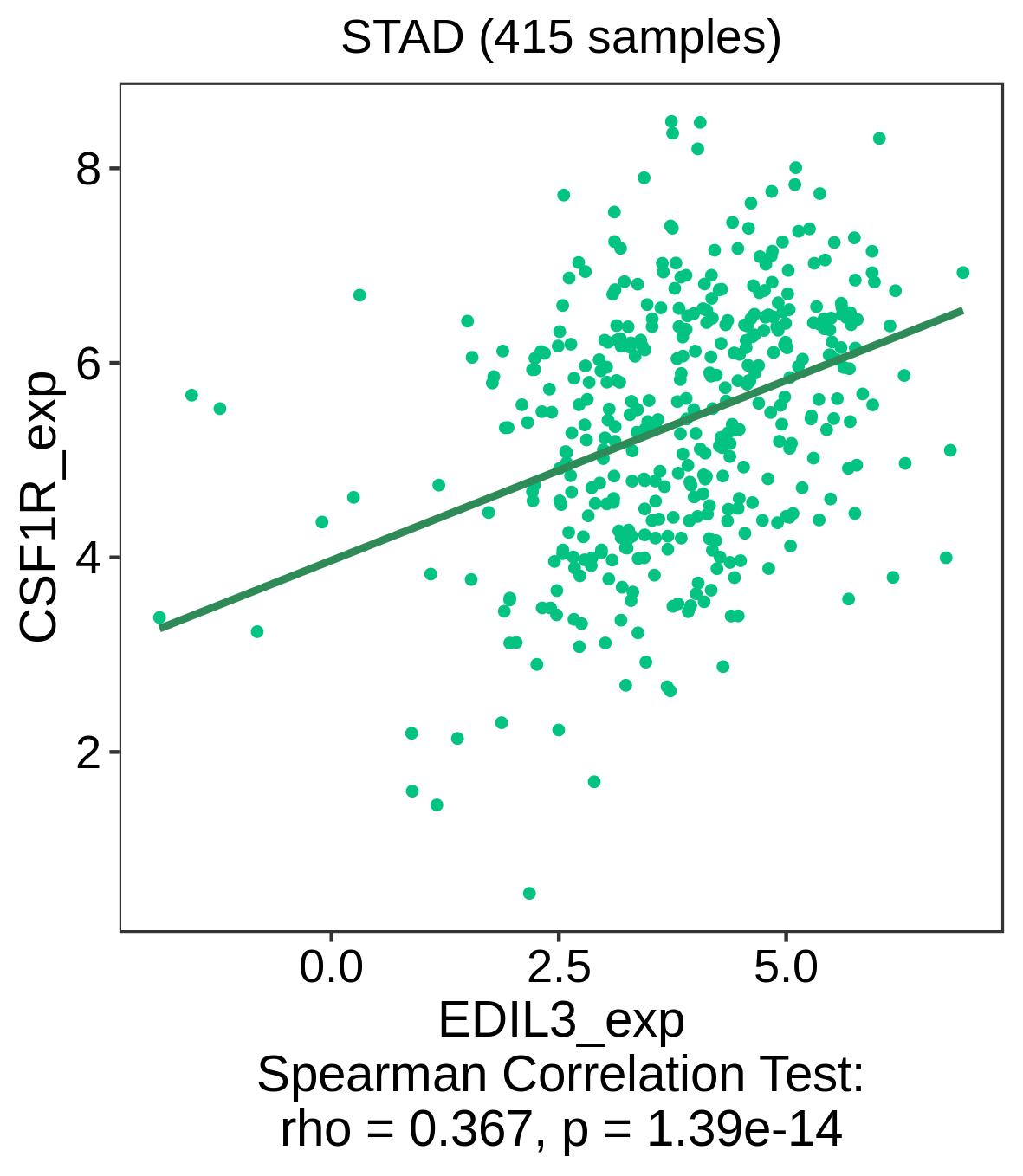

Supplement: Data S2 [file peerj-11-15559-s004.zip › Raw data 2/Raw figure 4-10/Figure 9/Fig 9C Immunoinhibitor/2 Immunoinhibitor_CSF1R.jpg]

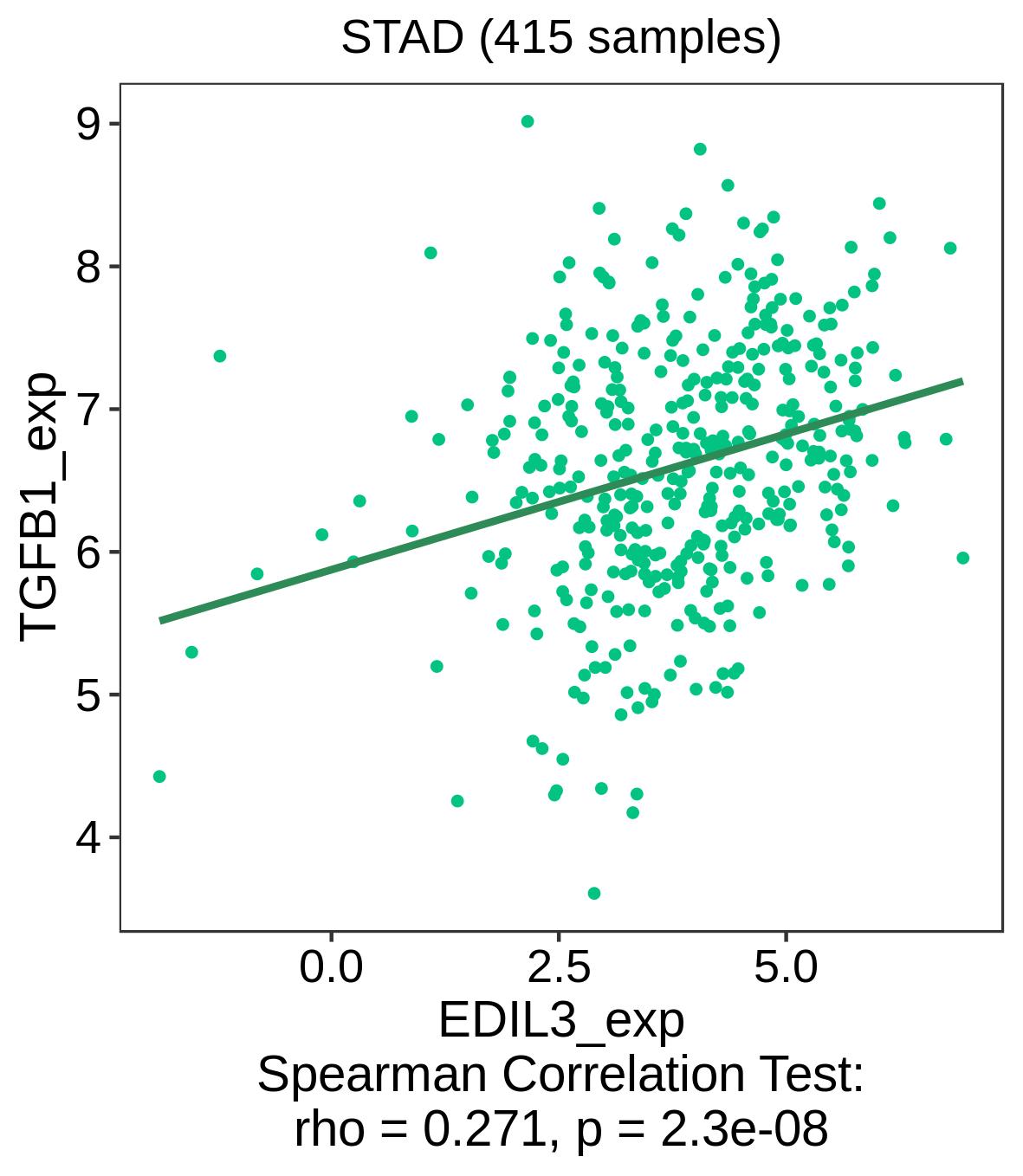

Supplement: Data S2 [file peerj-11-15559-s004.zip › Raw data 2/Raw figure 4-10/Figure 9/Fig 9C Immunoinhibitor/3 Immunoinhibitor_TGFB1.jpg]

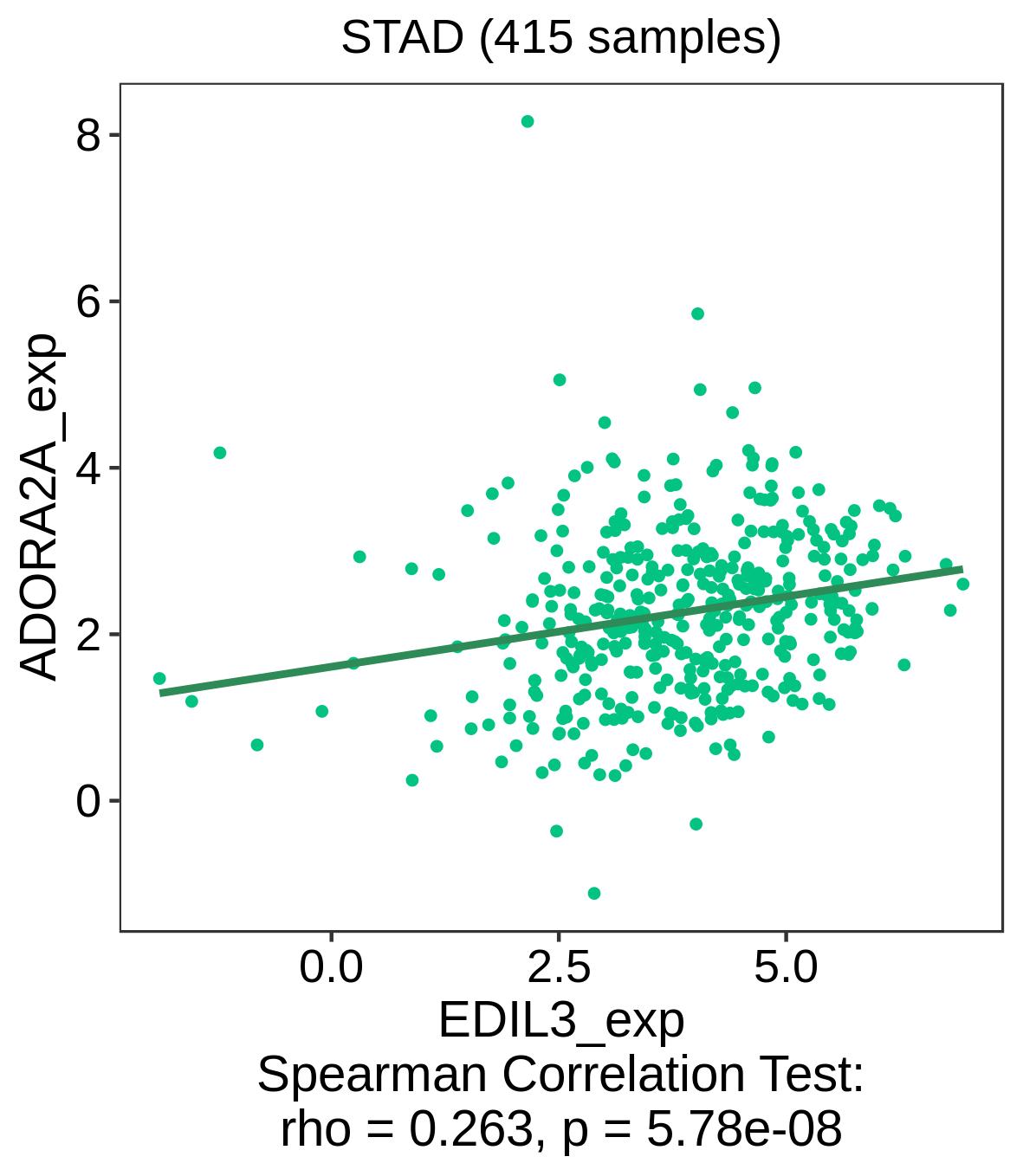

Supplement: Data S2 [file peerj-11-15559-s004.zip › Raw data 2/Raw figure 4-10/Figure 9/Fig 9C Immunoinhibitor/4_Immunoinhibitor_ADORA2A.jpg]

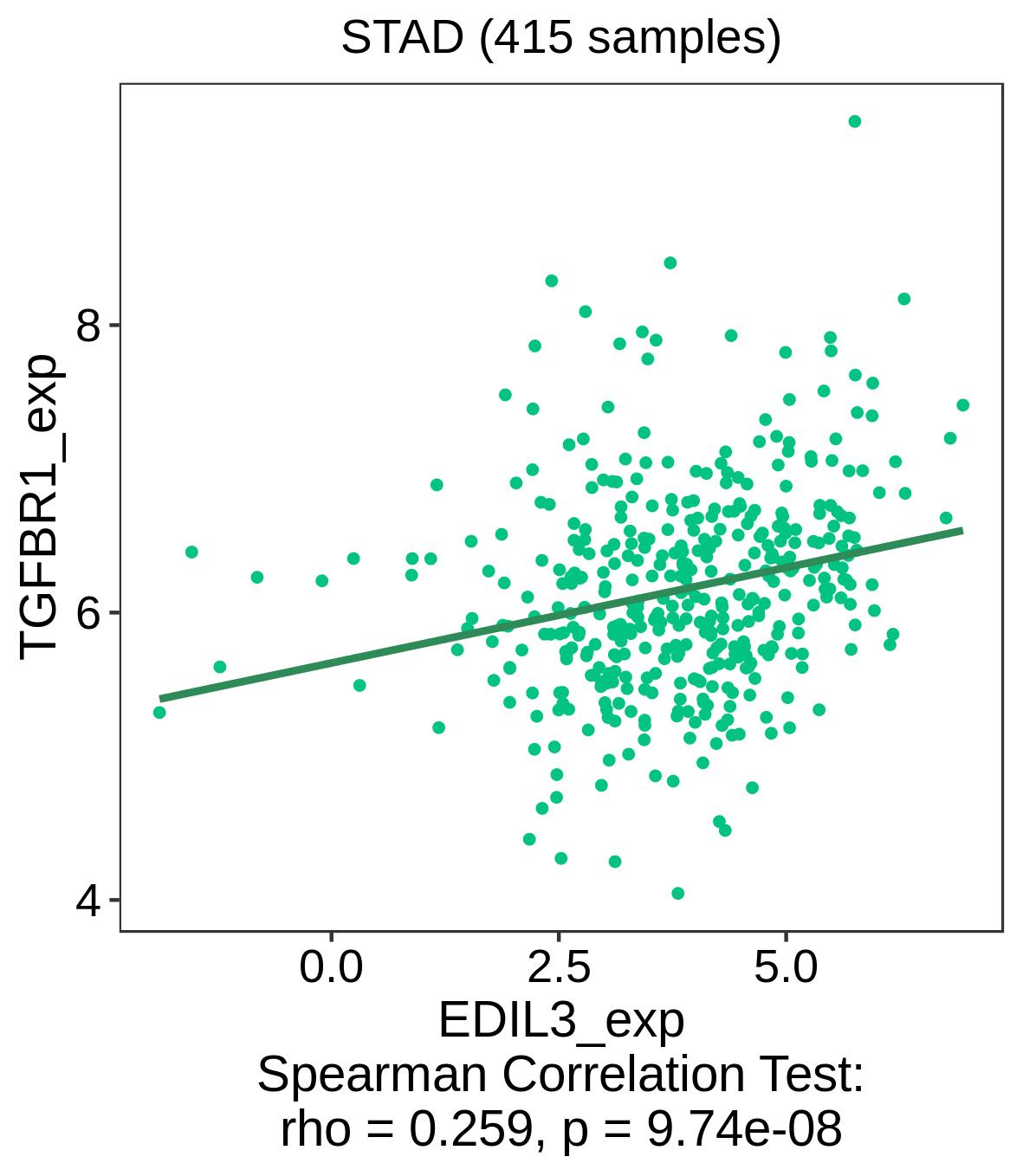

Supplement: Data S2 [file peerj-11-15559-s004.zip › Raw data 2/Raw figure 4-10/Figure 9/Fig 9C Immunoinhibitor/5 Immunoinhibitor_TGFBR1.jpg]

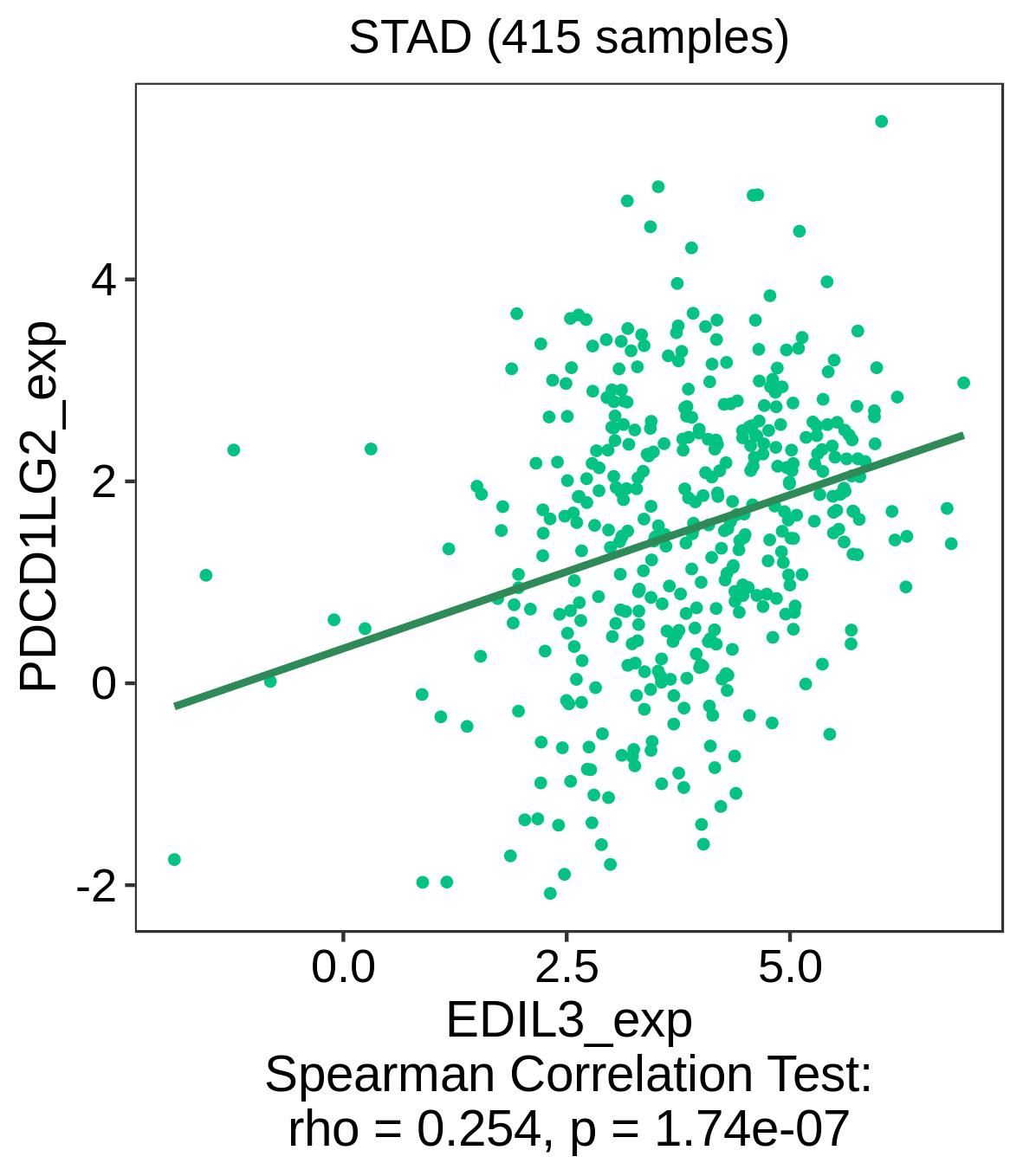

Supplement: Data S2 [file peerj-11-15559-s004.zip › Raw data 2/Raw figure 4-10/Figure 9/Fig 9C Immunoinhibitor/6 Immunoinhibitor_PDCD1LG2.jpg]

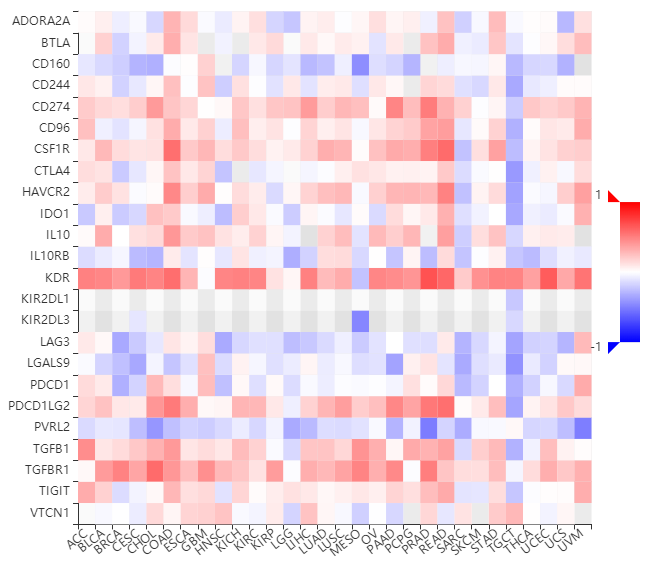

Supplement: Data S2 [file peerj-11-15559-s004.zip › Raw data 2/Raw figure 4-10/Figure 9/Fig 9C Immunoinhibitor/Immunoinhibitor.png]

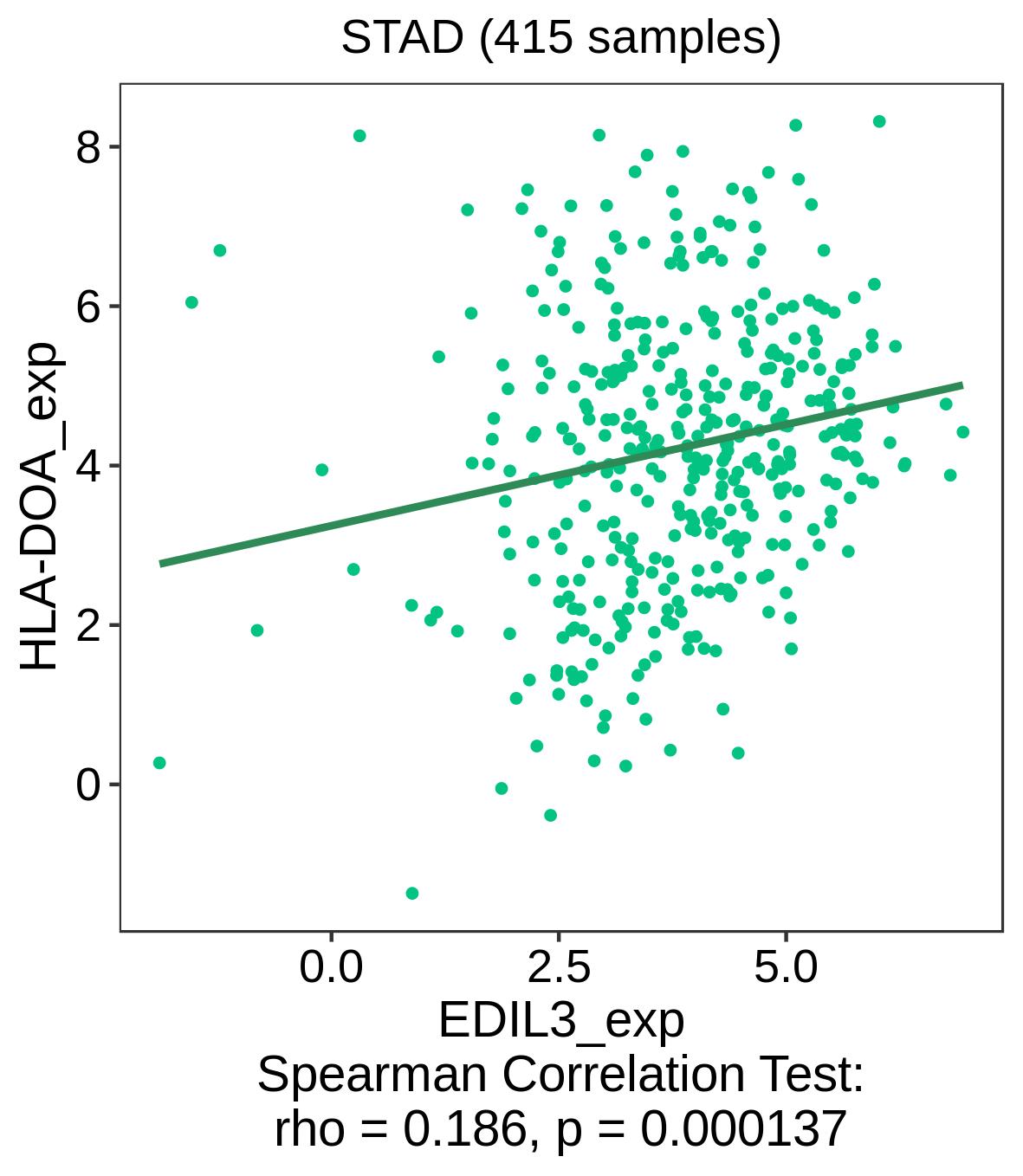

Supplement: Data S2 [file peerj-11-15559-s004.zip › Raw data 2/Raw figure 4-10/Figure 9/Fig 9D MHC molecule/1 MHC_HLA-DOA.jpg]

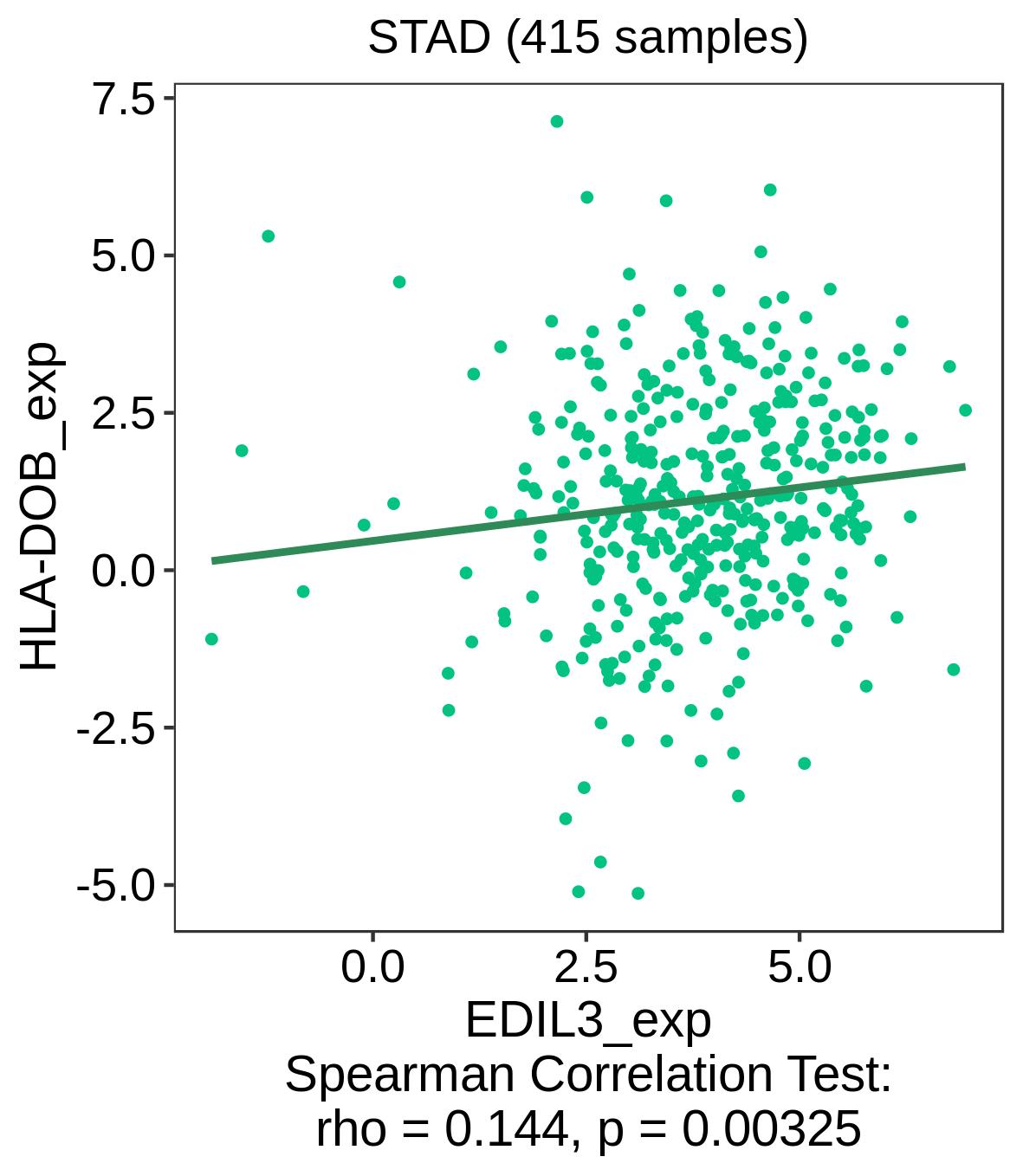

Supplement: Data S2 [file peerj-11-15559-s004.zip › Raw data 2/Raw figure 4-10/Figure 9/Fig 9D MHC molecule/2 MHC_HLA-DOB.jpg]

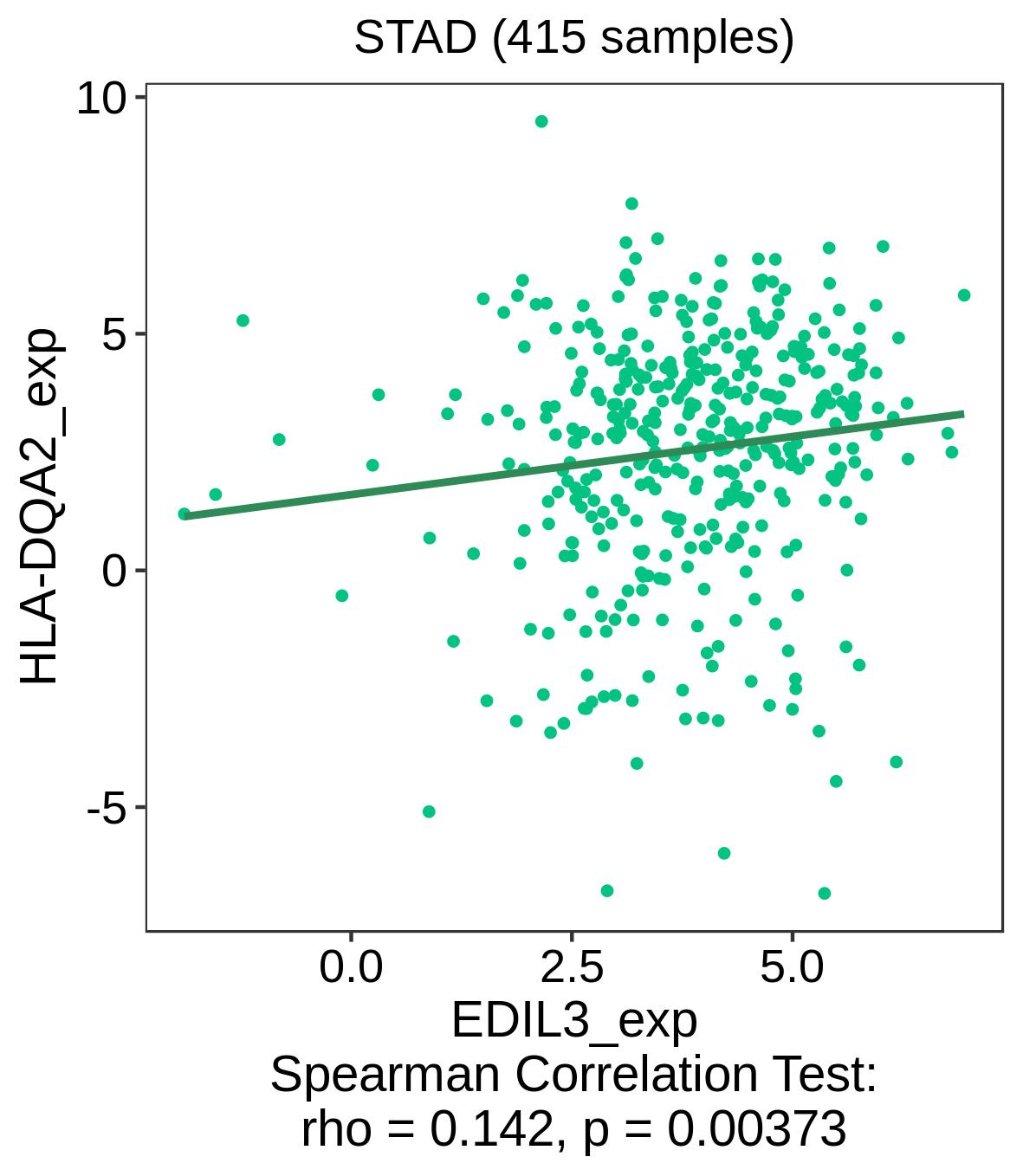

Supplement: Data S2 [file peerj-11-15559-s004.zip › Raw data 2/Raw figure 4-10/Figure 9/Fig 9D MHC molecule/3_MHC_HLA-DQA2.jpg]

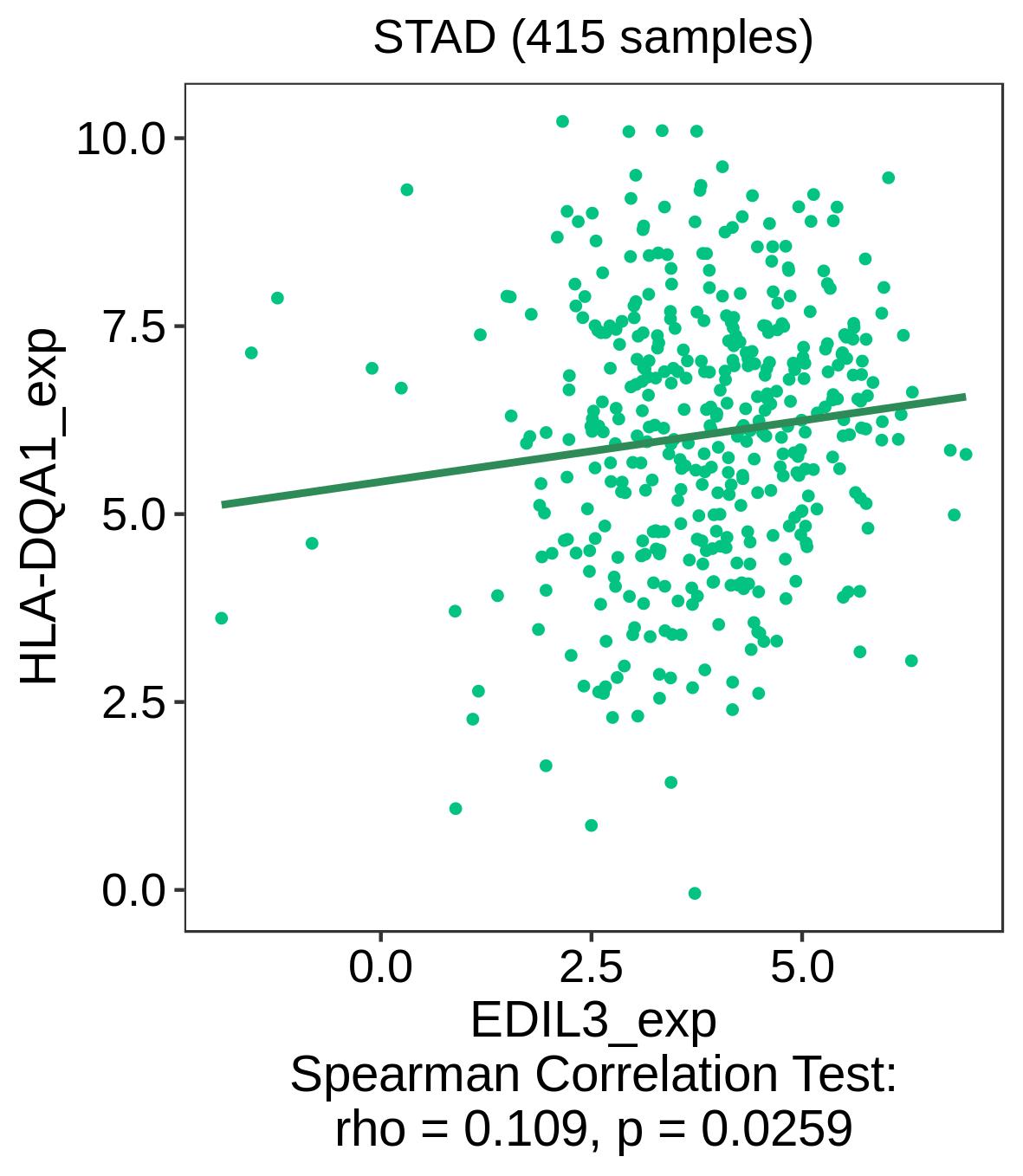

Supplement: Data S2 [file peerj-11-15559-s004.zip › Raw data 2/Raw figure 4-10/Figure 9/Fig 9D MHC molecule/4_MHC_HLA-DQA1.jpg]

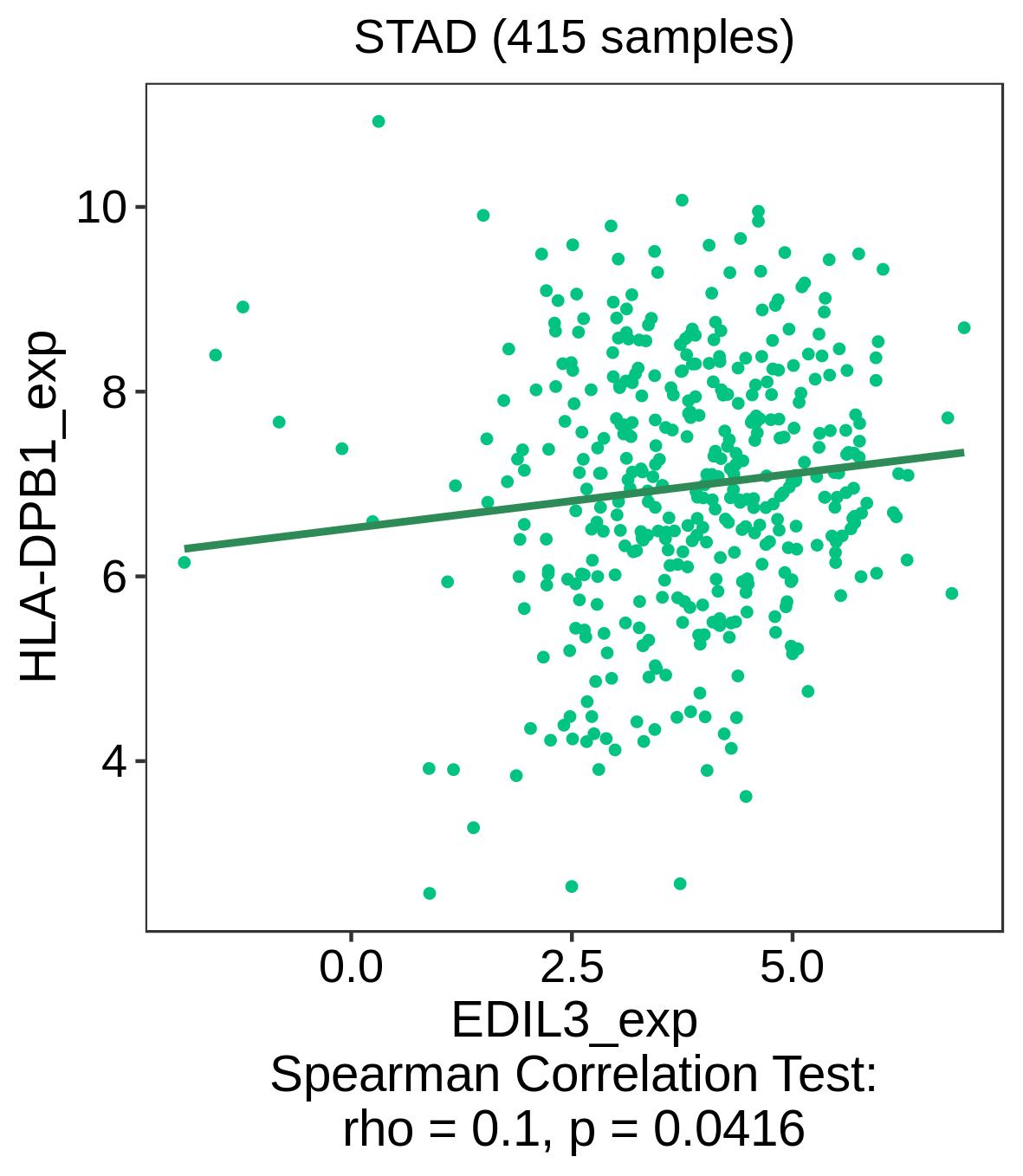

Supplement: Data S2 [file peerj-11-15559-s004.zip › Raw data 2/Raw figure 4-10/Figure 9/Fig 9D MHC molecule/5 MHC_HLA-DPB1.jpg]

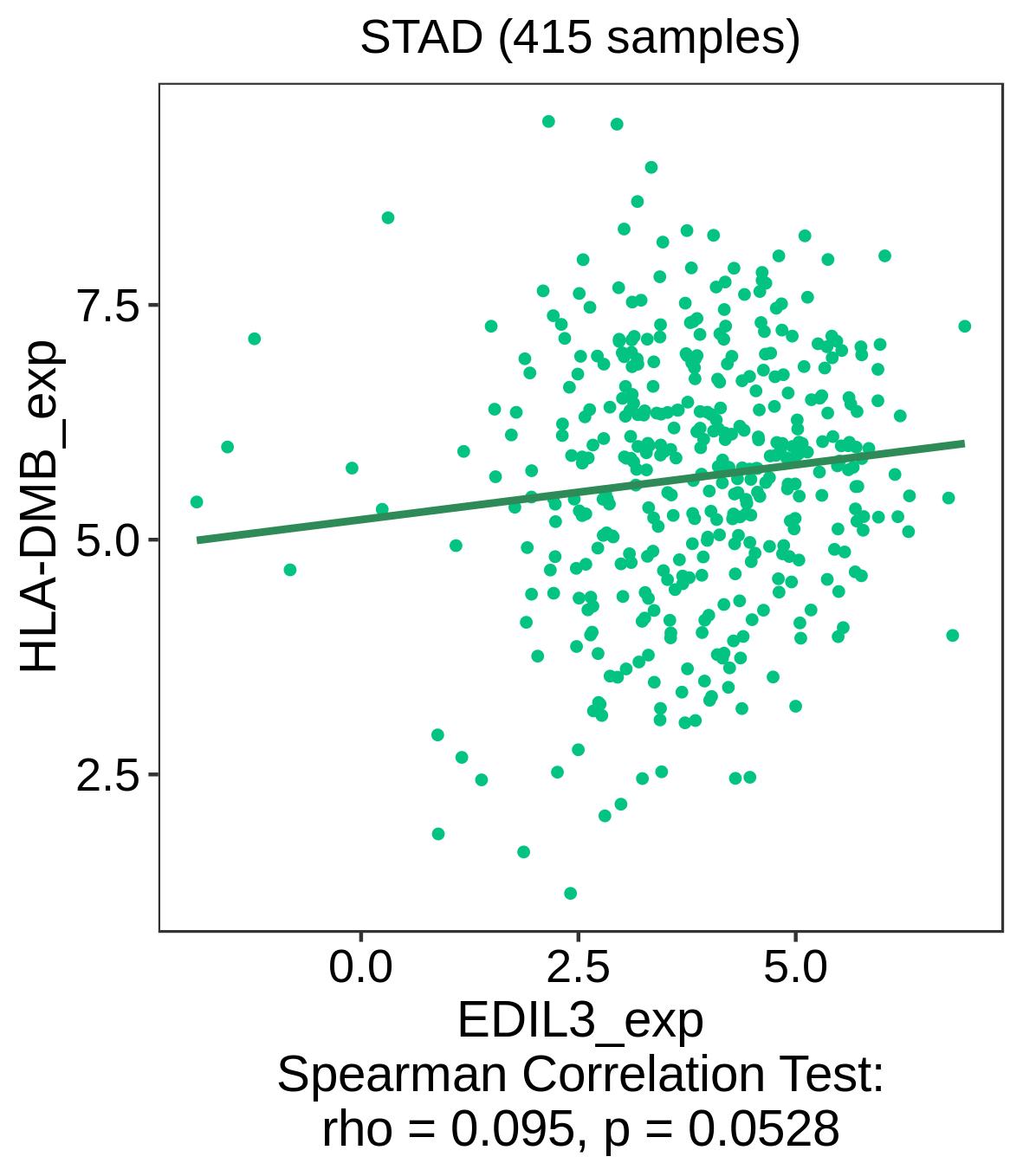

Supplement: Data S2 [file peerj-11-15559-s004.zip › Raw data 2/Raw figure 4-10/Figure 9/Fig 9D MHC molecule/6 ╥⌡╨╘ MHC_HLA-DMB.jpg]

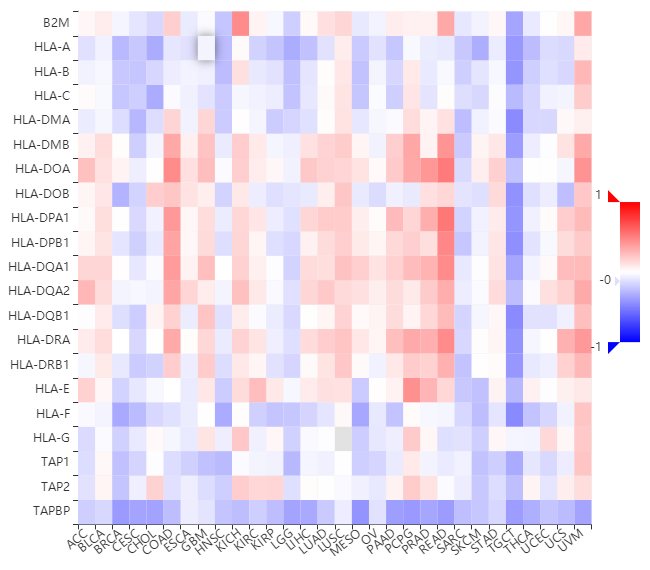

Supplement: Data S2 [file peerj-11-15559-s004.zip › Raw data 2/Raw figure 4-10/Figure 9/Fig 9D MHC molecule/MHC molecule.png]

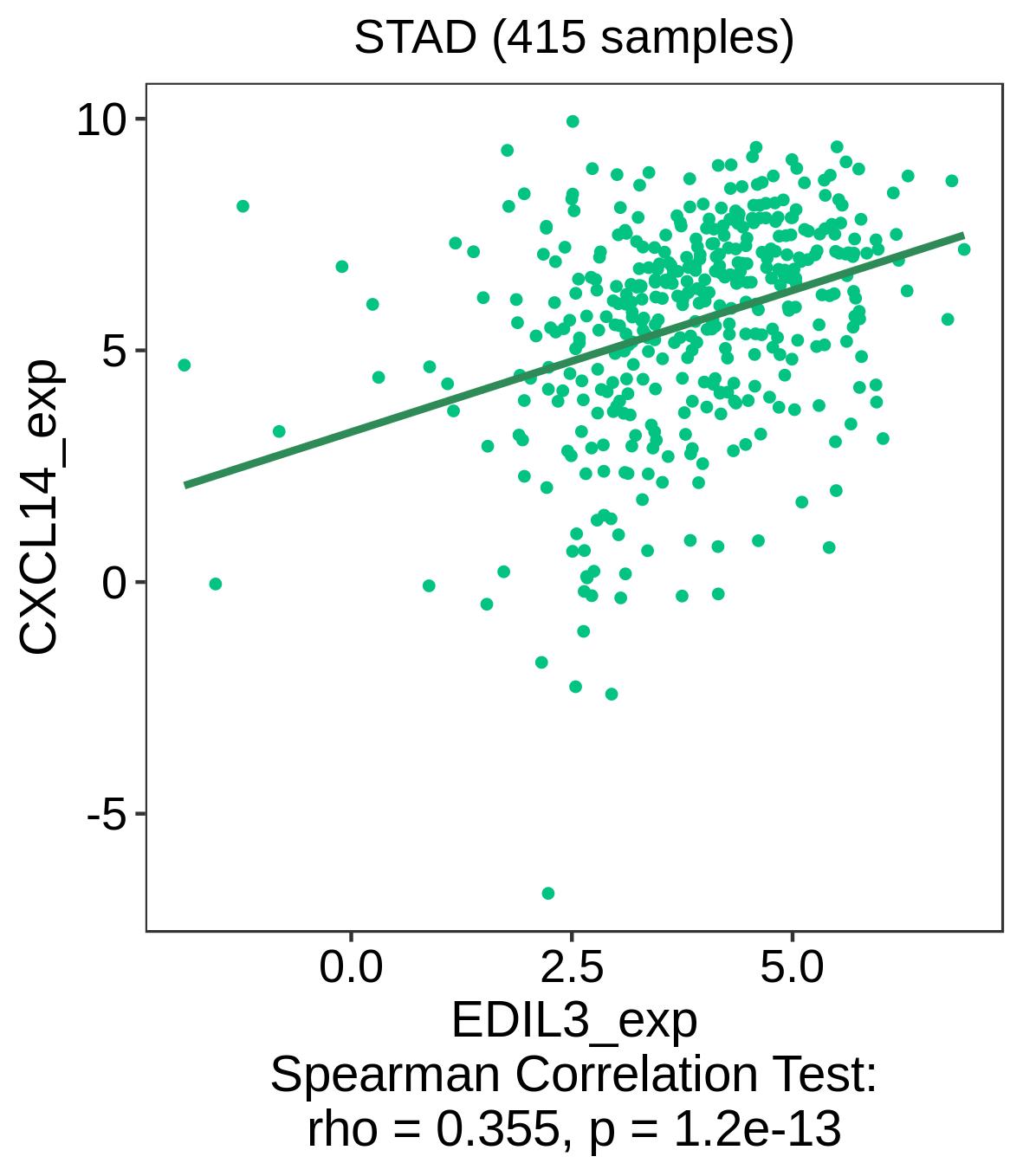

Supplement: Data S2 [file peerj-11-15559-s004.zip › Raw data 2/Raw figure 4-10/Figure 9/Fig 9E Chemokine/1 chemokine_CXCL14.jpg]

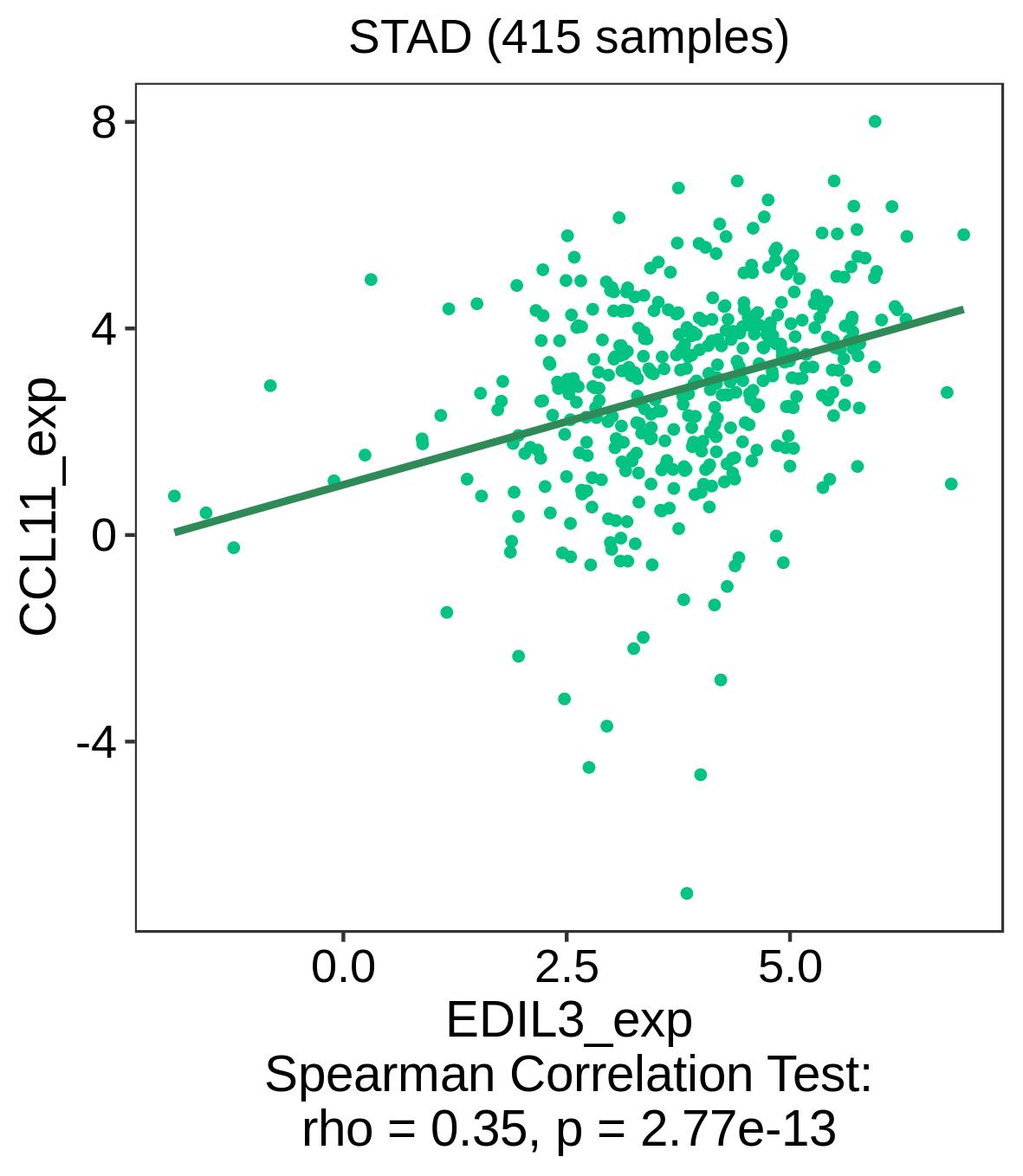

Supplement: Data S2 [file peerj-11-15559-s004.zip › Raw data 2/Raw figure 4-10/Figure 9/Fig 9E Chemokine/2 chemokine_CCL11.jpg]

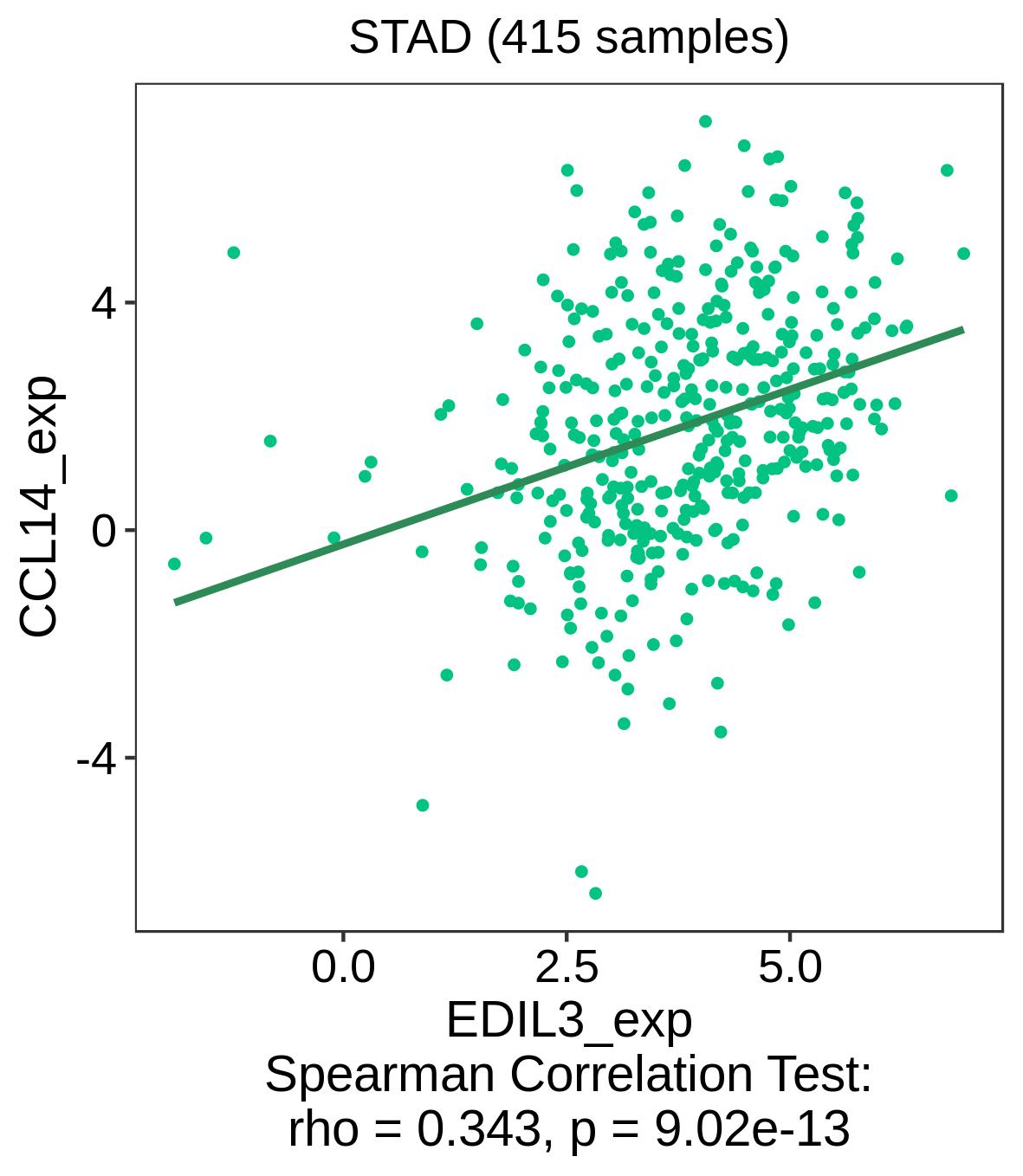

Supplement: Data S2 [file peerj-11-15559-s004.zip › Raw data 2/Raw figure 4-10/Figure 9/Fig 9E Chemokine/3_chemokine_CCL14.jpg]

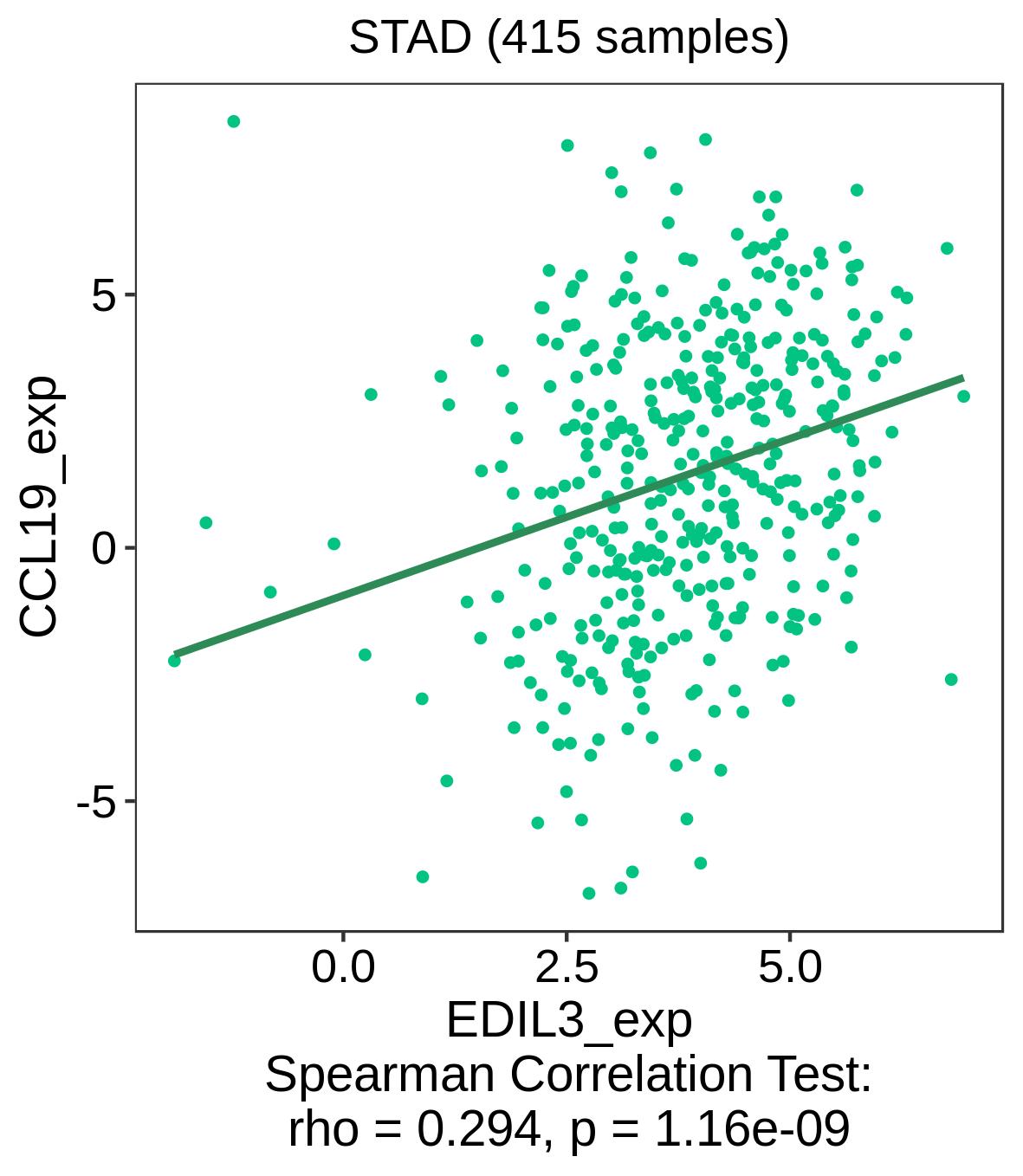

Supplement: Data S2 [file peerj-11-15559-s004.zip › Raw data 2/Raw figure 4-10/Figure 9/Fig 9E Chemokine/5_chemokine_CCL19.jpg]

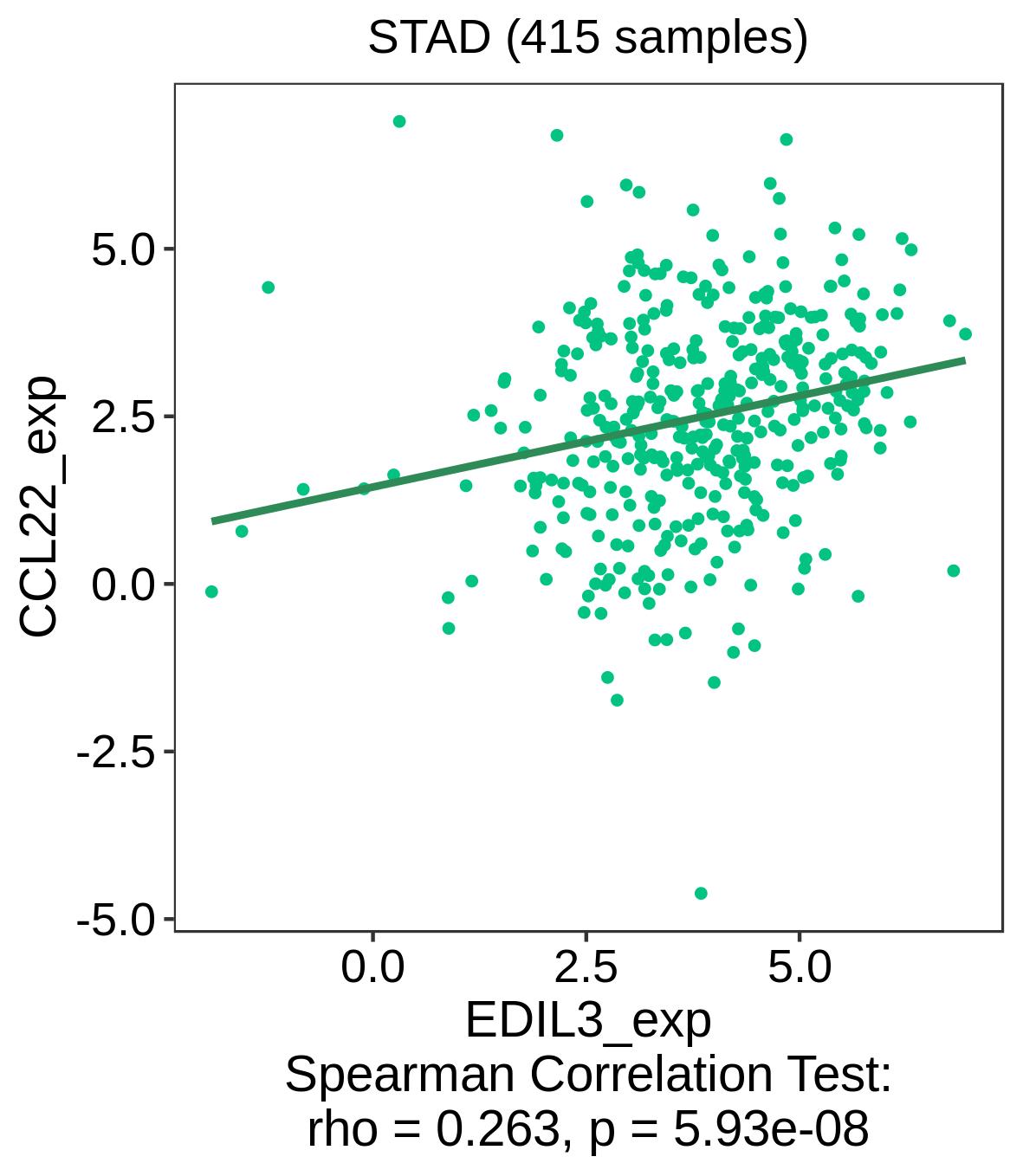

Supplement: Data S2 [file peerj-11-15559-s004.zip › Raw data 2/Raw figure 4-10/Figure 9/Fig 9E Chemokine/6_chemokine_CCL22.jpg]

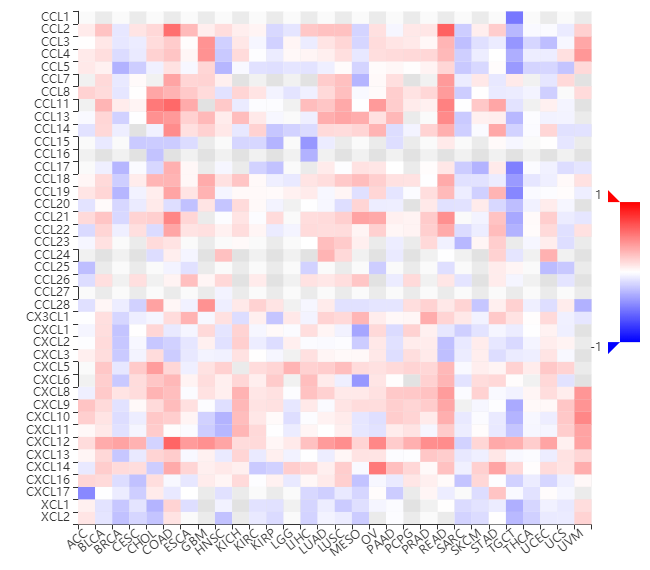

Supplement: Data S2 [file peerj-11-15559-s004.zip › Raw data 2/Raw figure 4-10/Figure 9/Fig 9E Chemokine/Chemokine.png]

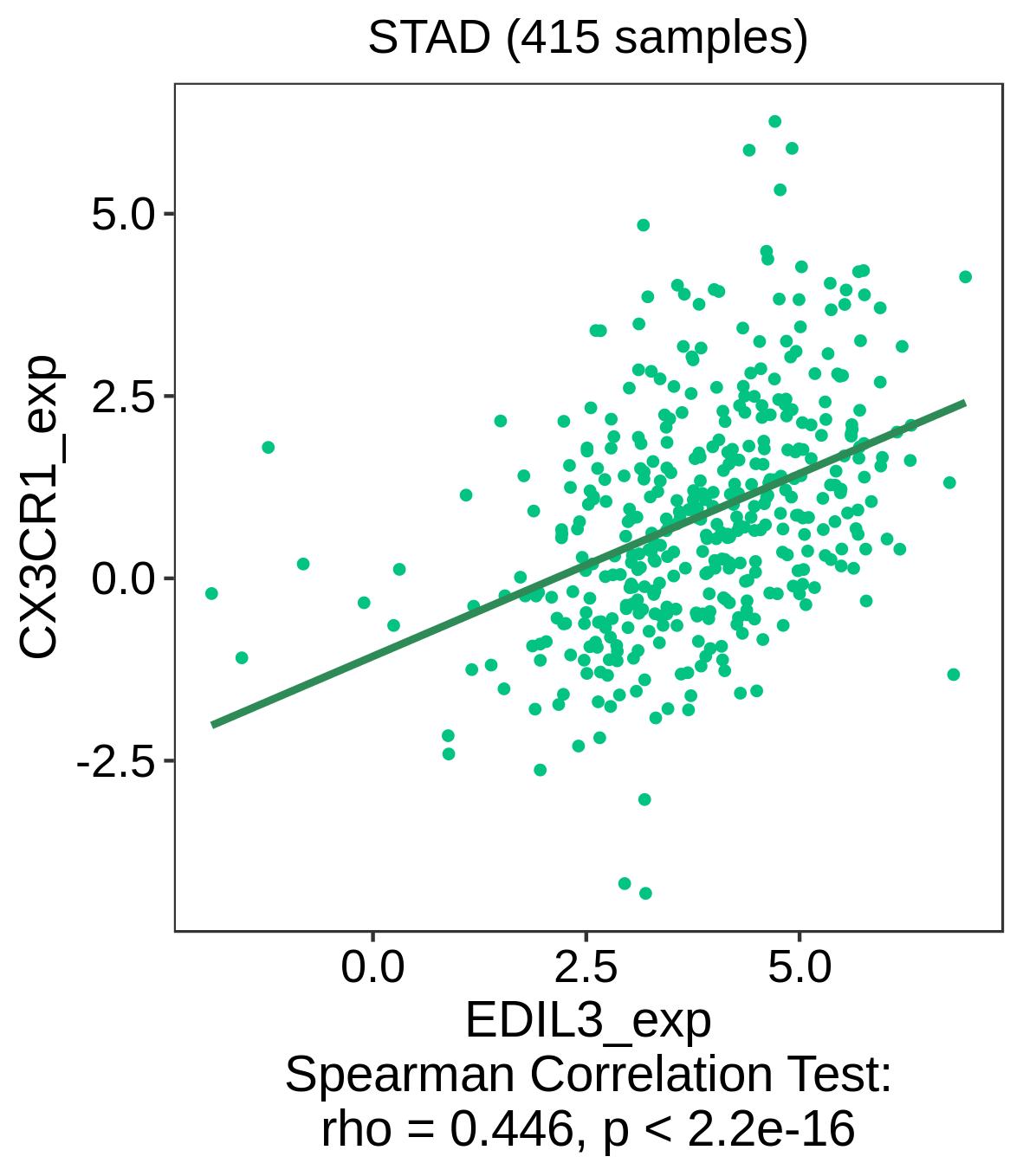

Supplement: Data S2 [file peerj-11-15559-s004.zip › Raw data 2/Raw figure 4-10/Figure 9/Fig 9F Receptor/1_receptor_CX3CR1.jpg]
