# Supplementary figures and images for: EDIL3 is a potential prognostic biomarker that correlates with immune infiltrates in gastric cancer (part 2 of 2)
Source: PeerJ. 2023 Aug 9;11:e15559. doi: 10.7717/peerj.15559 (PMC10422953; doi:10.7717/peerj.15559)

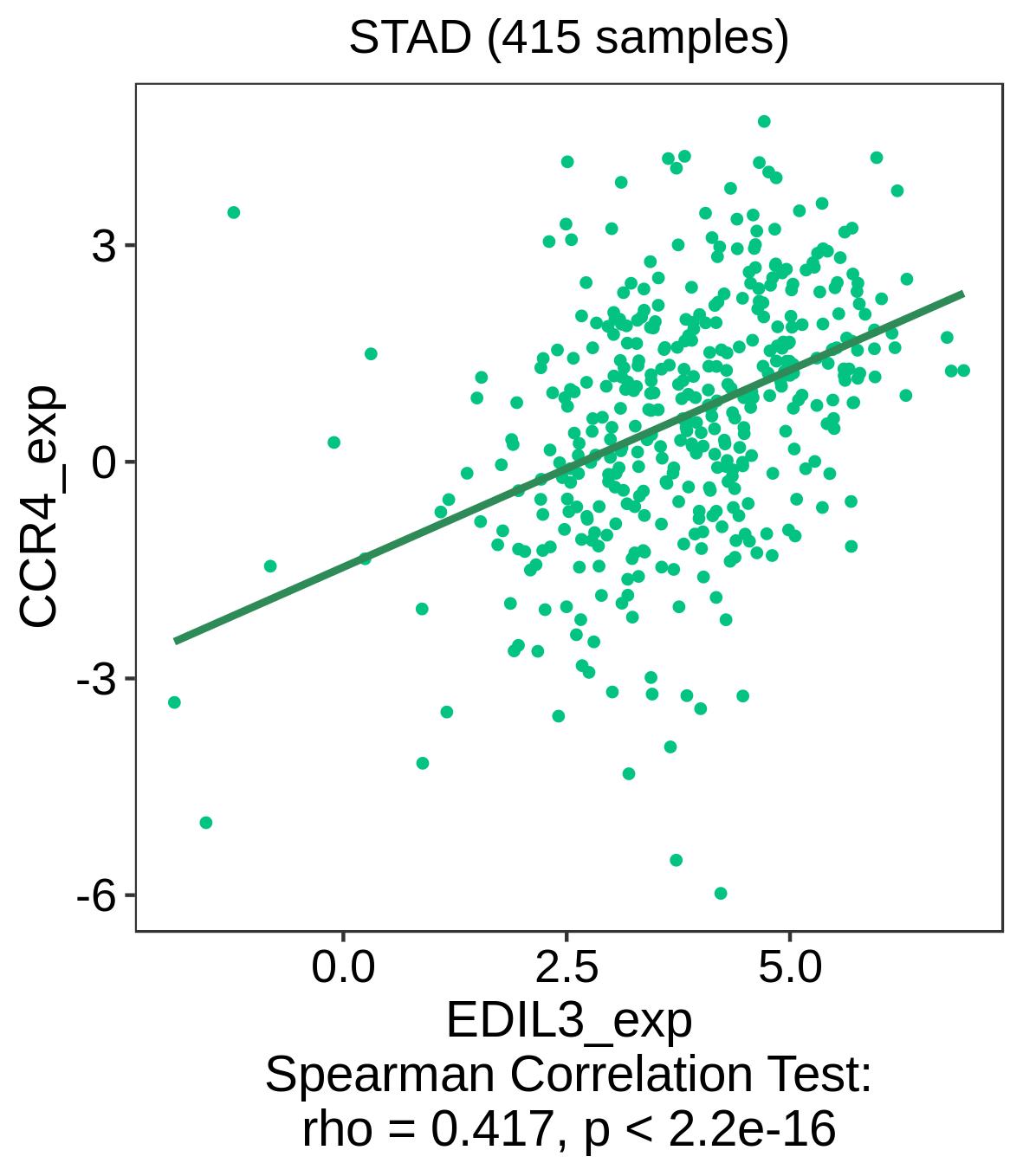

Supplement: Data S2 [file peerj-11-15559-s004.zip › Raw data 2/Raw figure 4-10/Figure 9/Fig 9F Receptor/2 receptor_CCR4.jpg]

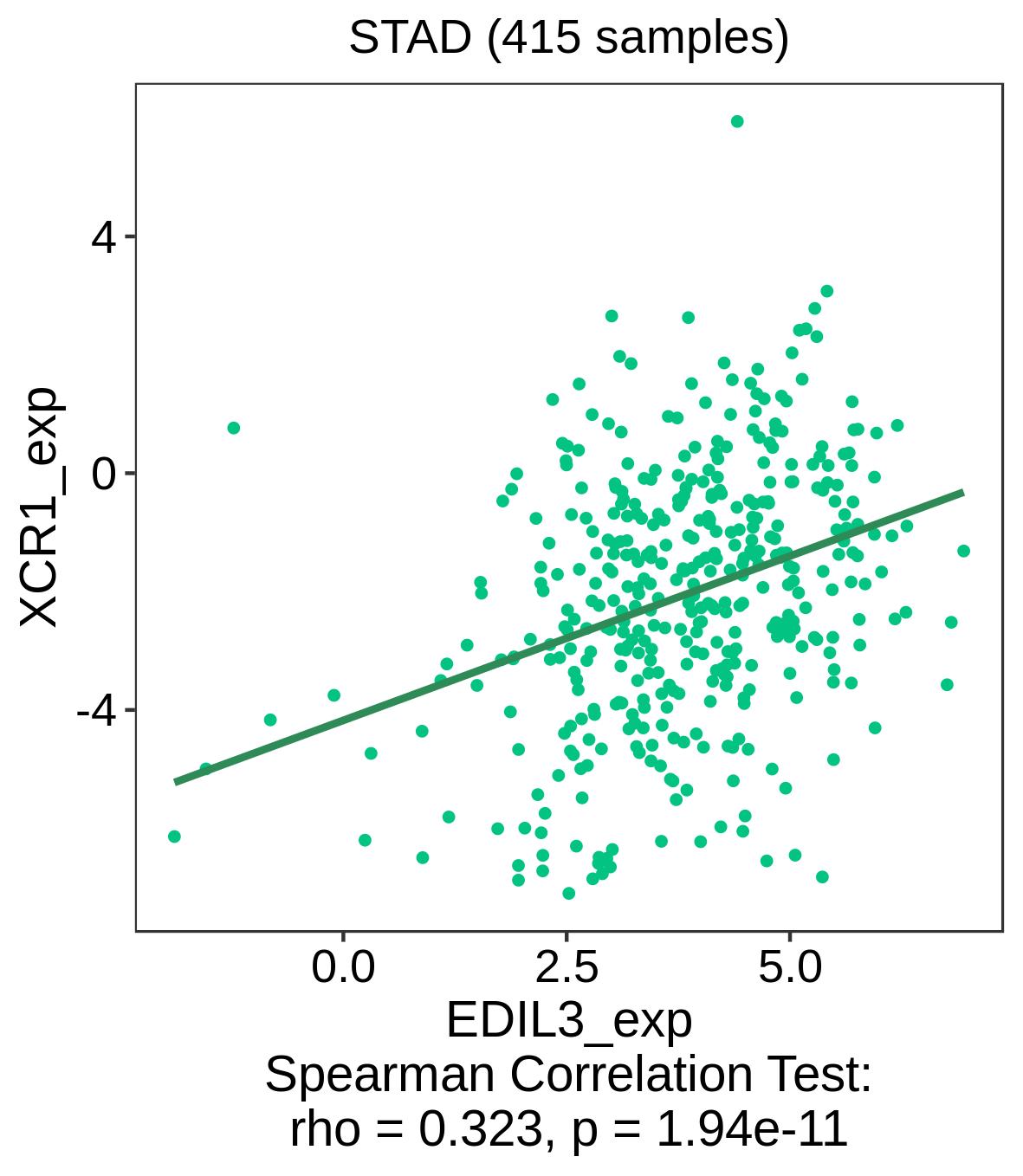

Supplement: Data S2 [file peerj-11-15559-s004.zip › Raw data 2/Raw figure 4-10/Figure 9/Fig 9F Receptor/3 receptor_XCR1.jpg]

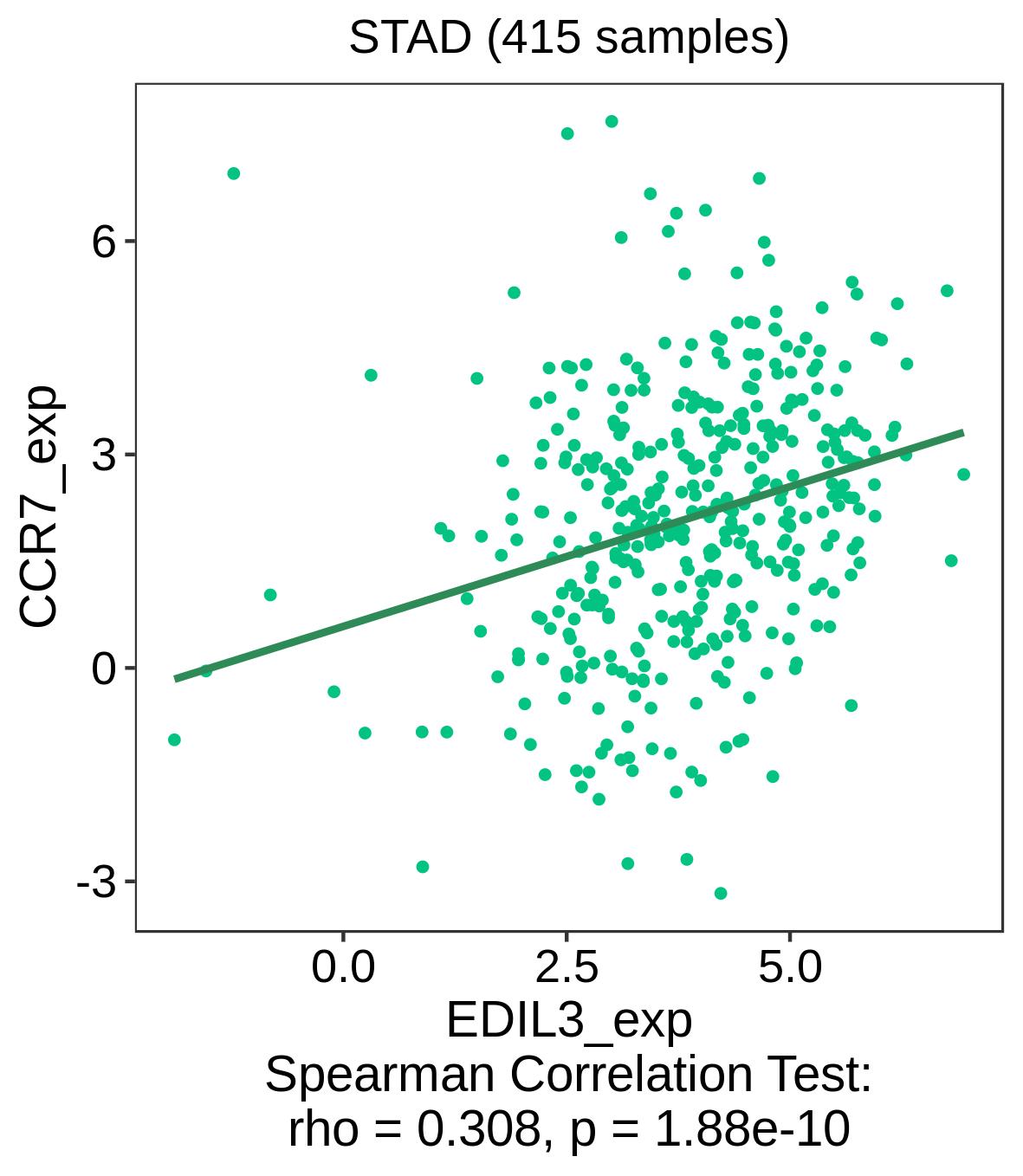

Supplement: Data S2 [file peerj-11-15559-s004.zip › Raw data 2/Raw figure 4-10/Figure 9/Fig 9F Receptor/4 receptor_CCR7.jpg]

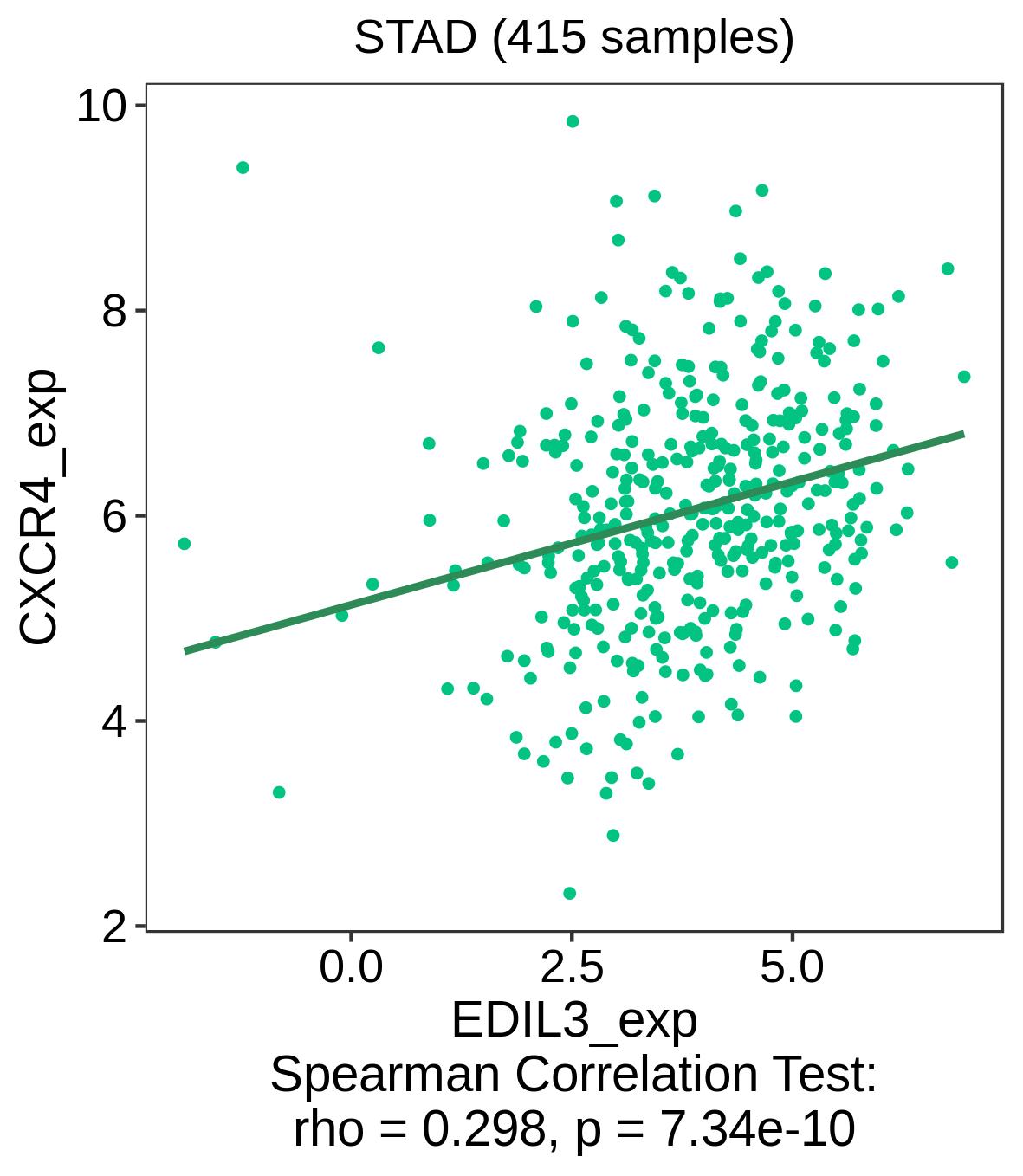

Supplement: Data S2 [file peerj-11-15559-s004.zip › Raw data 2/Raw figure 4-10/Figure 9/Fig 9F Receptor/5_receptor_CXCR4.jpg]

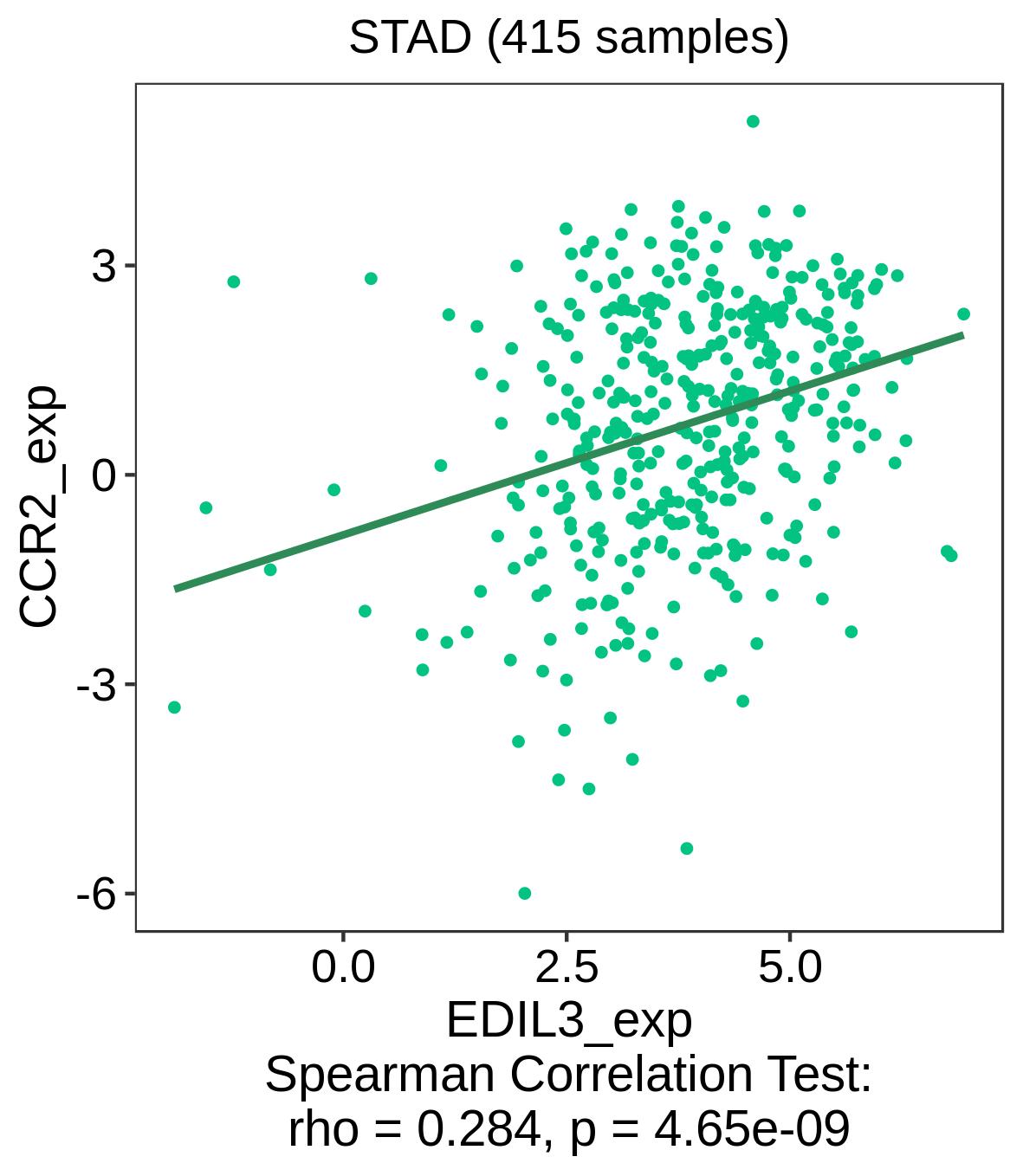

Supplement: Data S2 [file peerj-11-15559-s004.zip › Raw data 2/Raw figure 4-10/Figure 9/Fig 9F Receptor/6_receptor_CCR2.jpg]

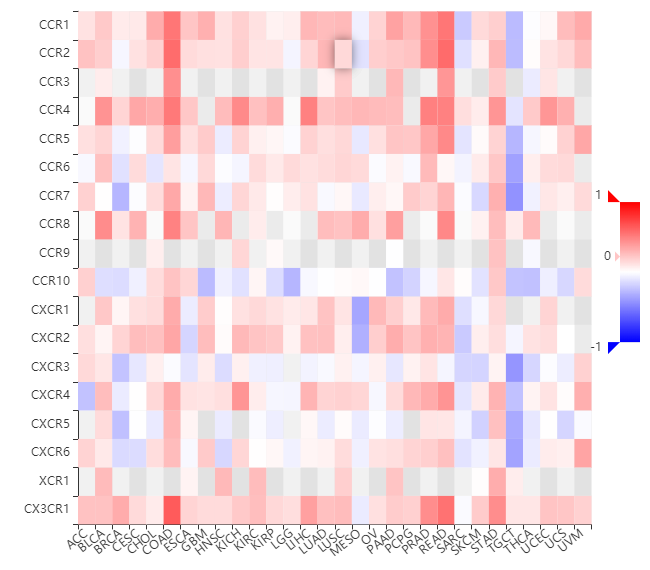

Supplement: Data S2 [file peerj-11-15559-s004.zip › Raw data 2/Raw figure 4-10/Figure 9/Fig 9F Receptor/Receptor.png]

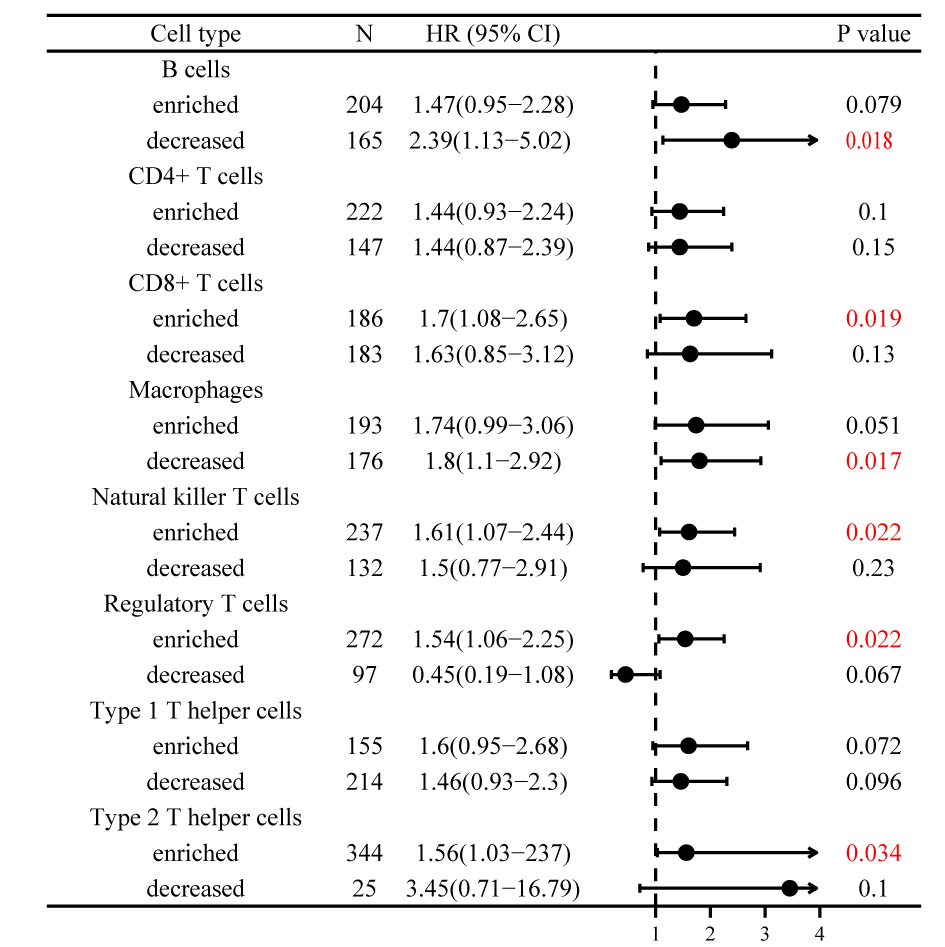

Supplement: Data S2 [file peerj-11-15559-s004.zip › Raw data 2/Raw figure 4-10/figure 10/Fig 10A.tif]

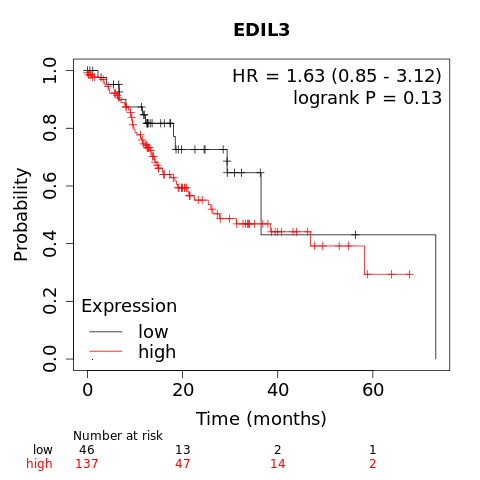

Supplement: Data S2 [file peerj-11-15559-s004.zip › Raw data 2/Raw figure 4-10/figure 10/Fig 10B CD8 decreased EDIL3.png]

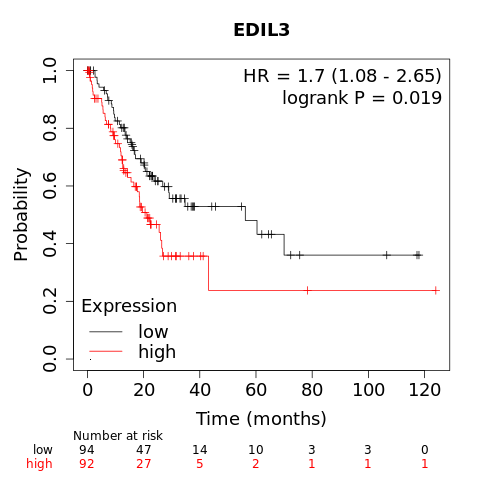

Supplement: Data S2 [file peerj-11-15559-s004.zip › Raw data 2/Raw figure 4-10/figure 10/Fig 10B CD8 enriched_EDIL3.png]

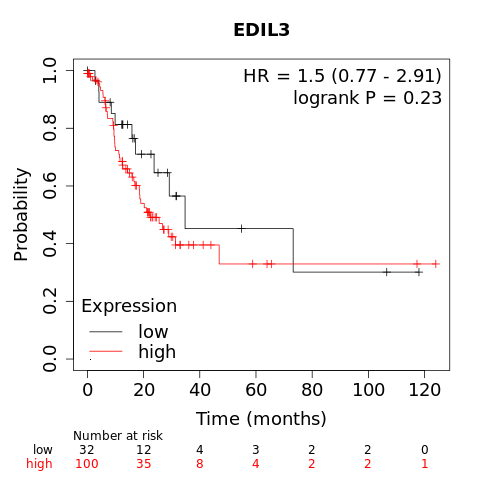

Supplement: Data S2 [file peerj-11-15559-s004.zip › Raw data 2/Raw figure 4-10/figure 10/Fig 10C NK decreased_EDIL3.png]

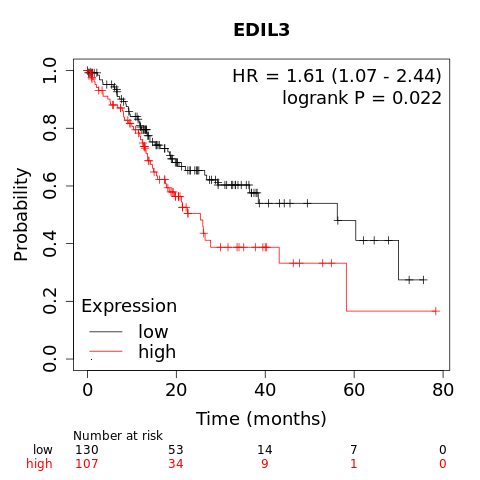

Supplement: Data S2 [file peerj-11-15559-s004.zip › Raw data 2/Raw figure 4-10/figure 10/Fig 10C NK enriched.png]

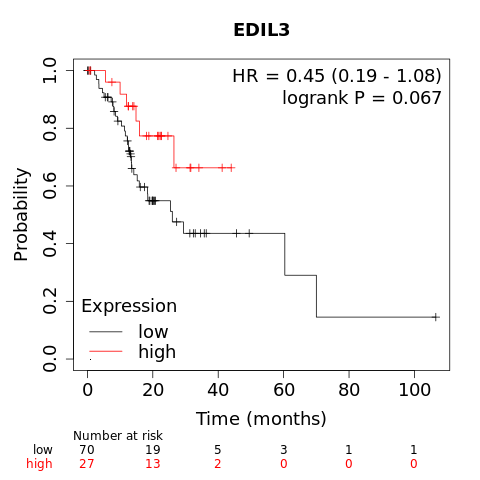

Supplement: Data S2 [file peerj-11-15559-s004.zip › Raw data 2/Raw figure 4-10/figure 10/Fig 10D Regulatory T decreased_EDIL3.png]

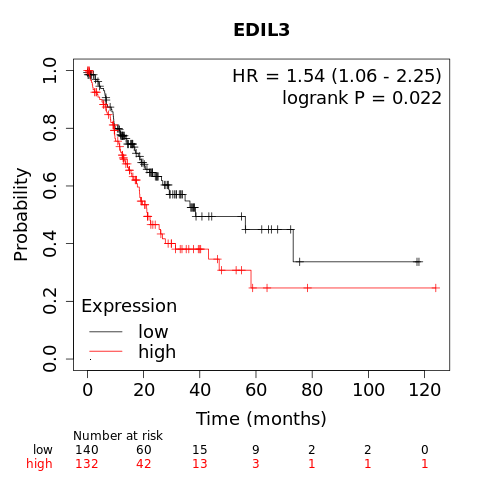

Supplement: Data S2 [file peerj-11-15559-s004.zip › Raw data 2/Raw figure 4-10/figure 10/Fig 10D Regulatory T enriched_EDIL3.png]

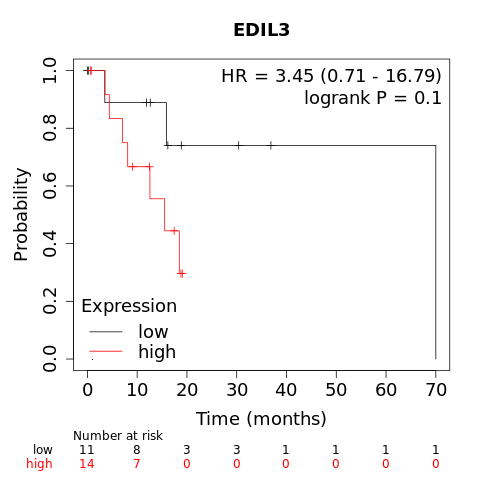

Supplement: Data S2 [file peerj-11-15559-s004.zip › Raw data 2/Raw figure 4-10/figure 10/Fig 10E Type 2 T-helper decreased_EDIL3.png]

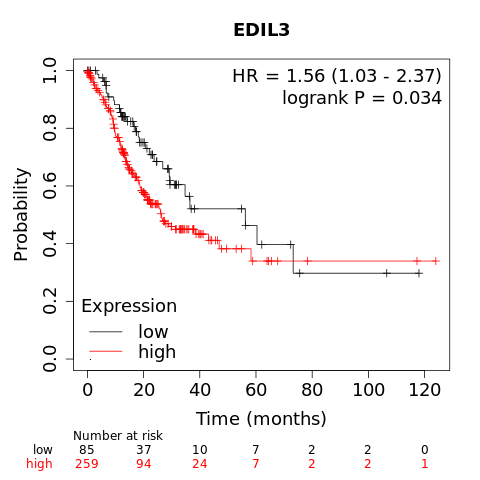

Supplement: Data S2 [file peerj-11-15559-s004.zip › Raw data 2/Raw figure 4-10/figure 10/Fig 10E Type 2 T-helper enriched_EDIL3.png]

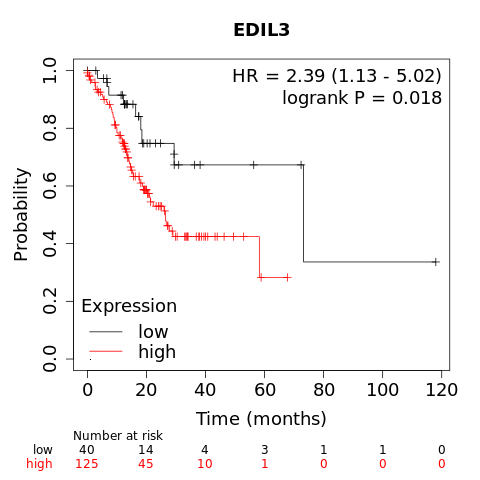

Supplement: Data S2 [file peerj-11-15559-s004.zip › Raw data 2/Raw figure 4-10/figure 10/Fig 10F decreased B cell.png]

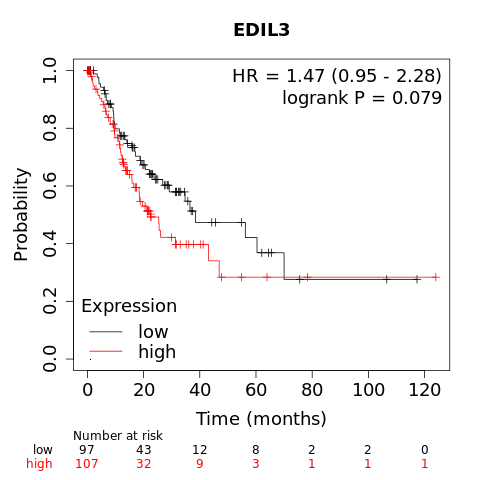

Supplement: Data S2 [file peerj-11-15559-s004.zip › Raw data 2/Raw figure 4-10/figure 10/Fig 10F enriched B cell EDIL3.png]

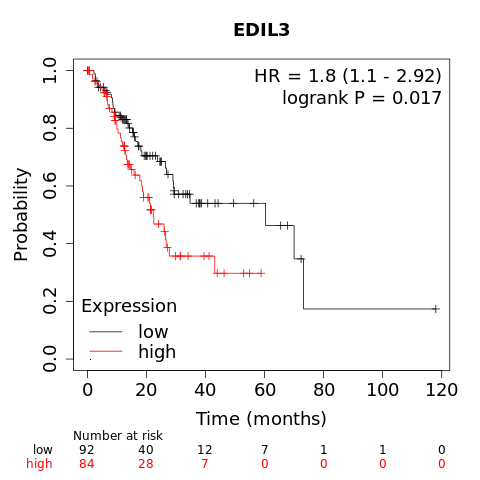

Supplement: Data S2 [file peerj-11-15559-s004.zip › Raw data 2/Raw figure 4-10/figure 10/Fig 10G Macrophages decreased_EDIL3.png]

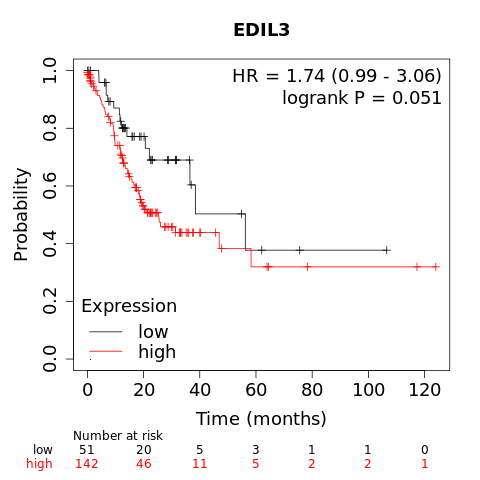

Supplement: Data S2 [file peerj-11-15559-s004.zip › Raw data 2/Raw figure 4-10/figure 10/Fig 10G Macrophages enriched_EDIL3.png]

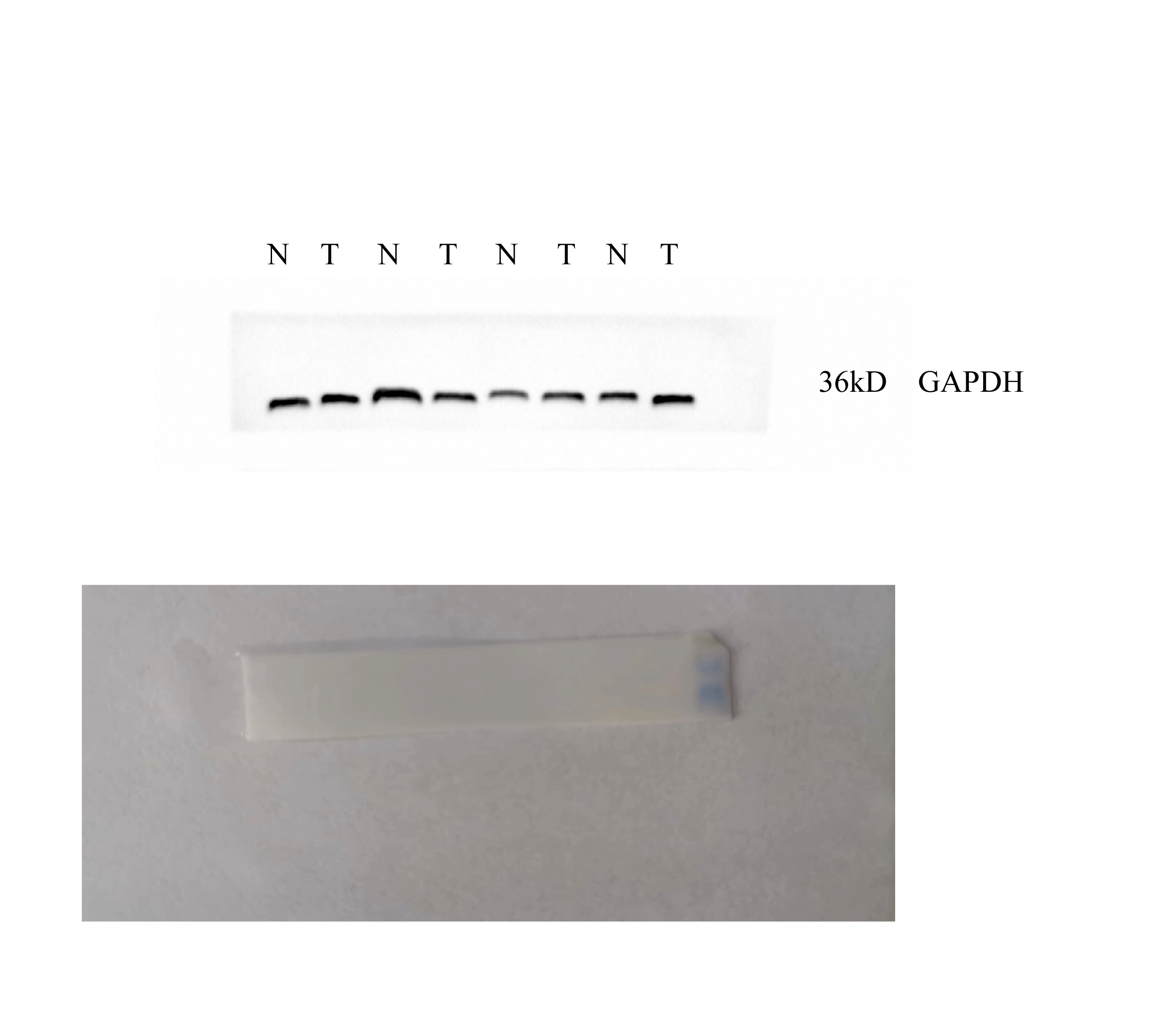

Supplement: Supplemental Information 5 — The protein extracts were separated and then electrotransferred to PVDF membranes. The PVDF membranes were cut at the level of the 36 and 52 kDa. The membranes were incubated with primary antibody and probed with HRP-coupled secondary antibody. Finally, the bands were visualized by using ECL reagents. [file peerj-11-15559-s005.zip › GAPDH.tif]

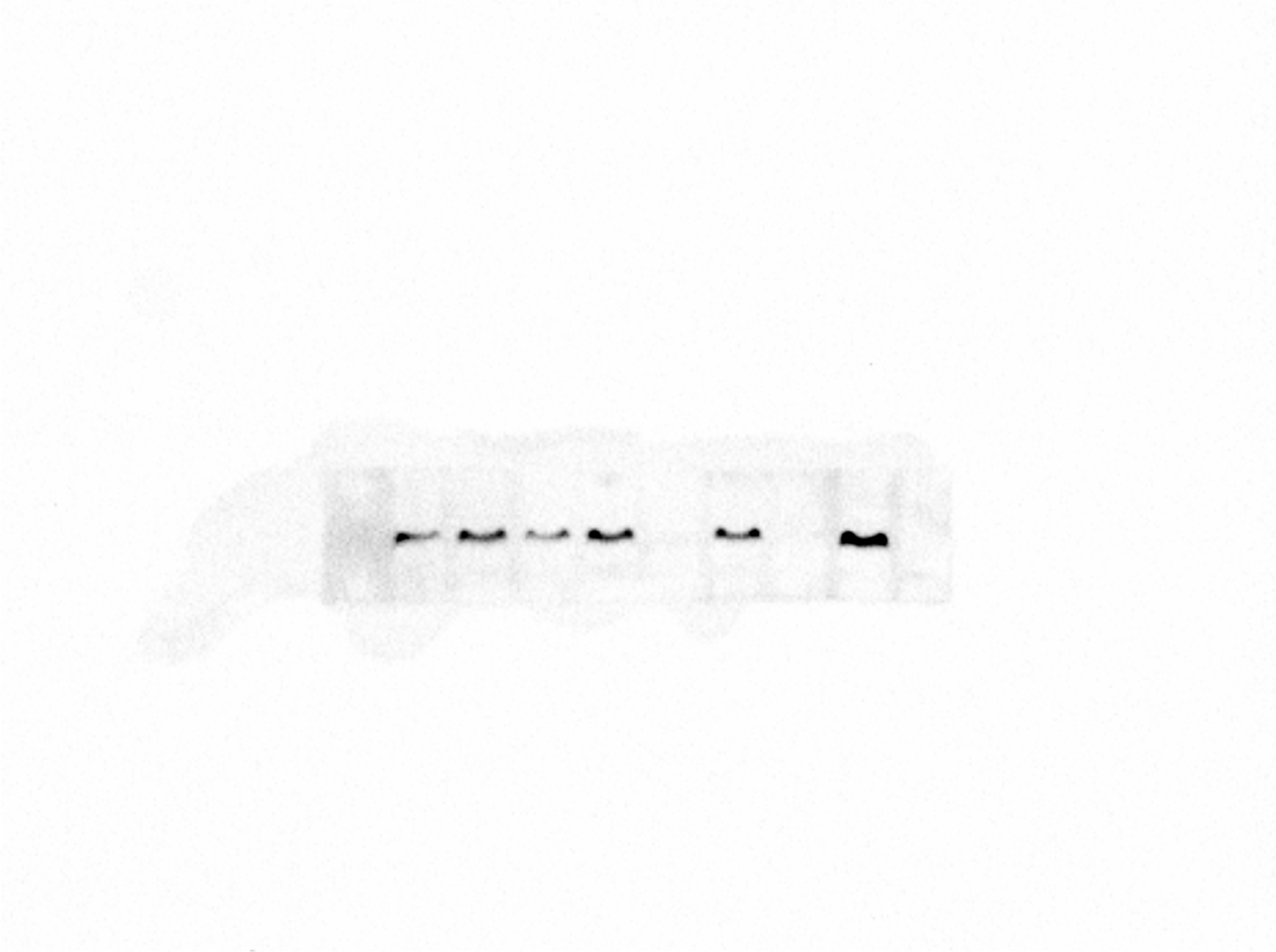

Supplement: Supplemental Information 5 — The protein extracts were separated and then electrotransferred to PVDF membranes. The PVDF membranes were cut at the level of the 36 and 52 kDa. The membranes were incubated with primary antibody and probed with HRP-coupled secondary antibody. Finally, the bands were visualized by using ECL reagents. [file peerj-11-15559-s005.zip › Raw EDIL3 -01.tif]

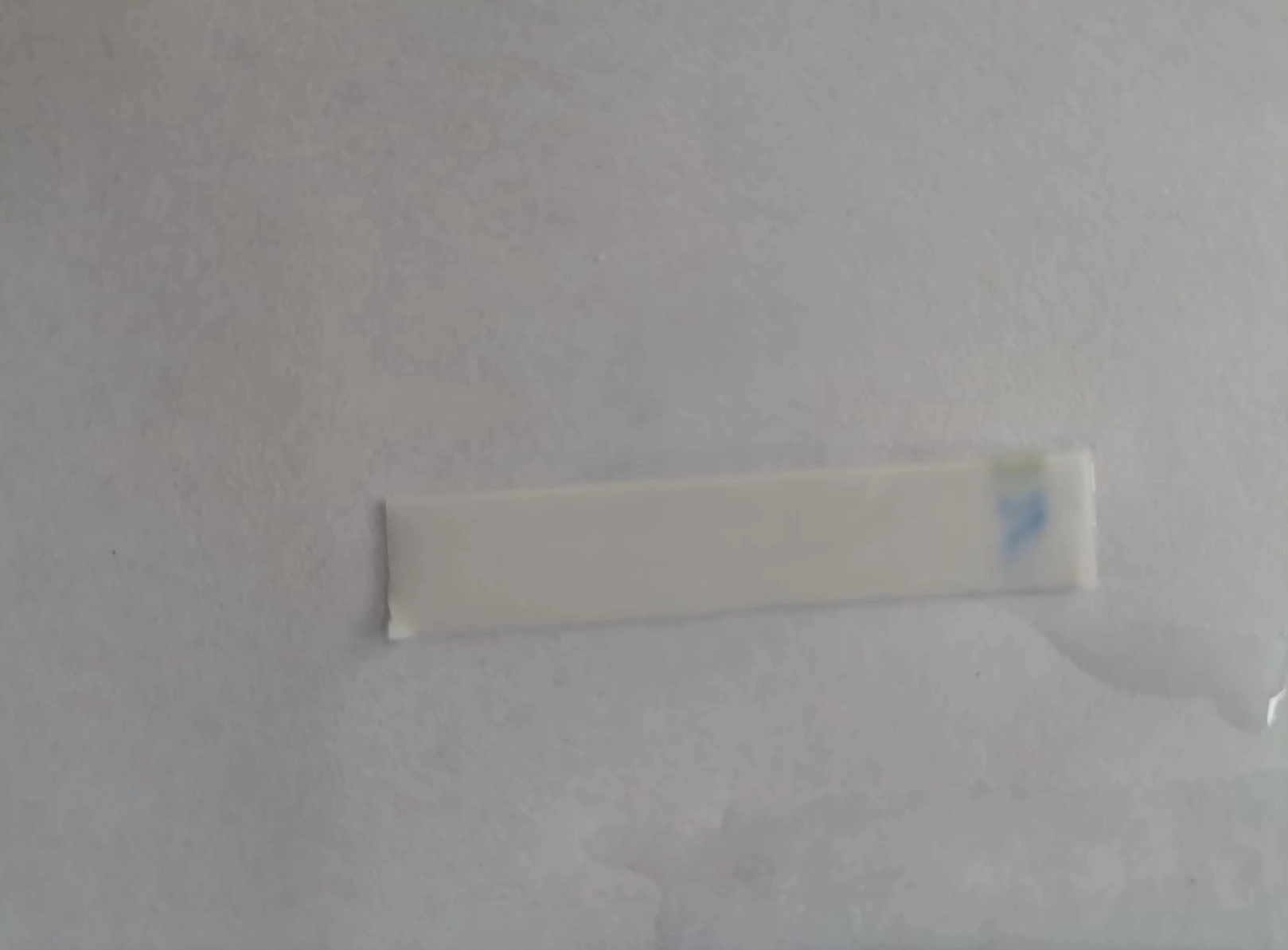

Supplement: Supplemental Information 5 — The protein extracts were separated and then electrotransferred to PVDF membranes. The PVDF membranes were cut at the level of the 36 and 52 kDa. The membranes were incubated with primary antibody and probed with HRP-coupled secondary antibody. Finally, the bands were visualized by using ECL reagents. [file peerj-11-15559-s005.zip › Raw EDIL3-02.tif]

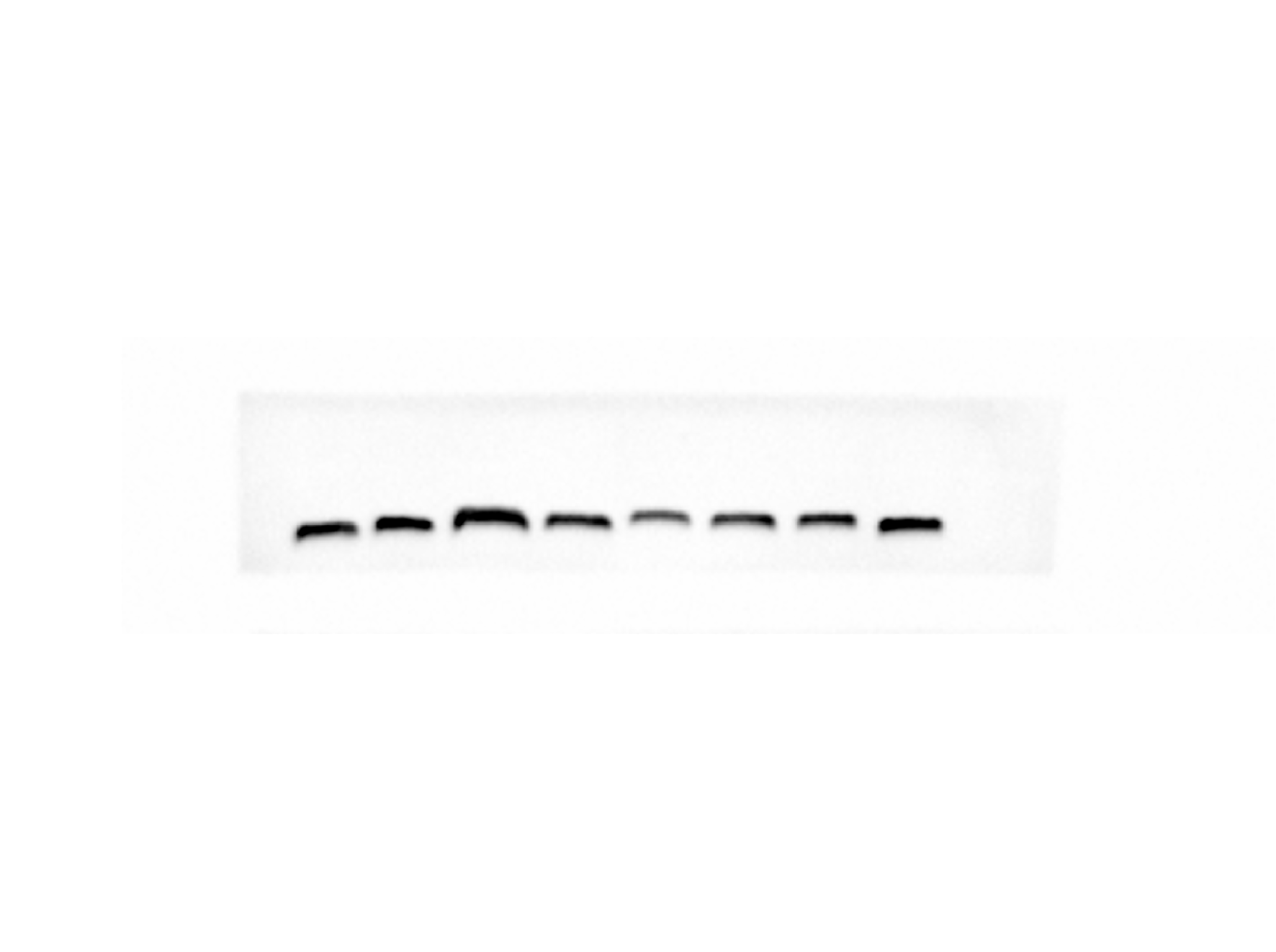

Supplement: Supplemental Information 5 — The protein extracts were separated and then electrotransferred to PVDF membranes. The PVDF membranes were cut at the level of the 36 and 52 kDa. The membranes were incubated with primary antibody and probed with HRP-coupled secondary antibody. Finally, the bands were visualized by using ECL reagents. [file peerj-11-15559-s005.zip › Raw GAPDH-01-01.tif]

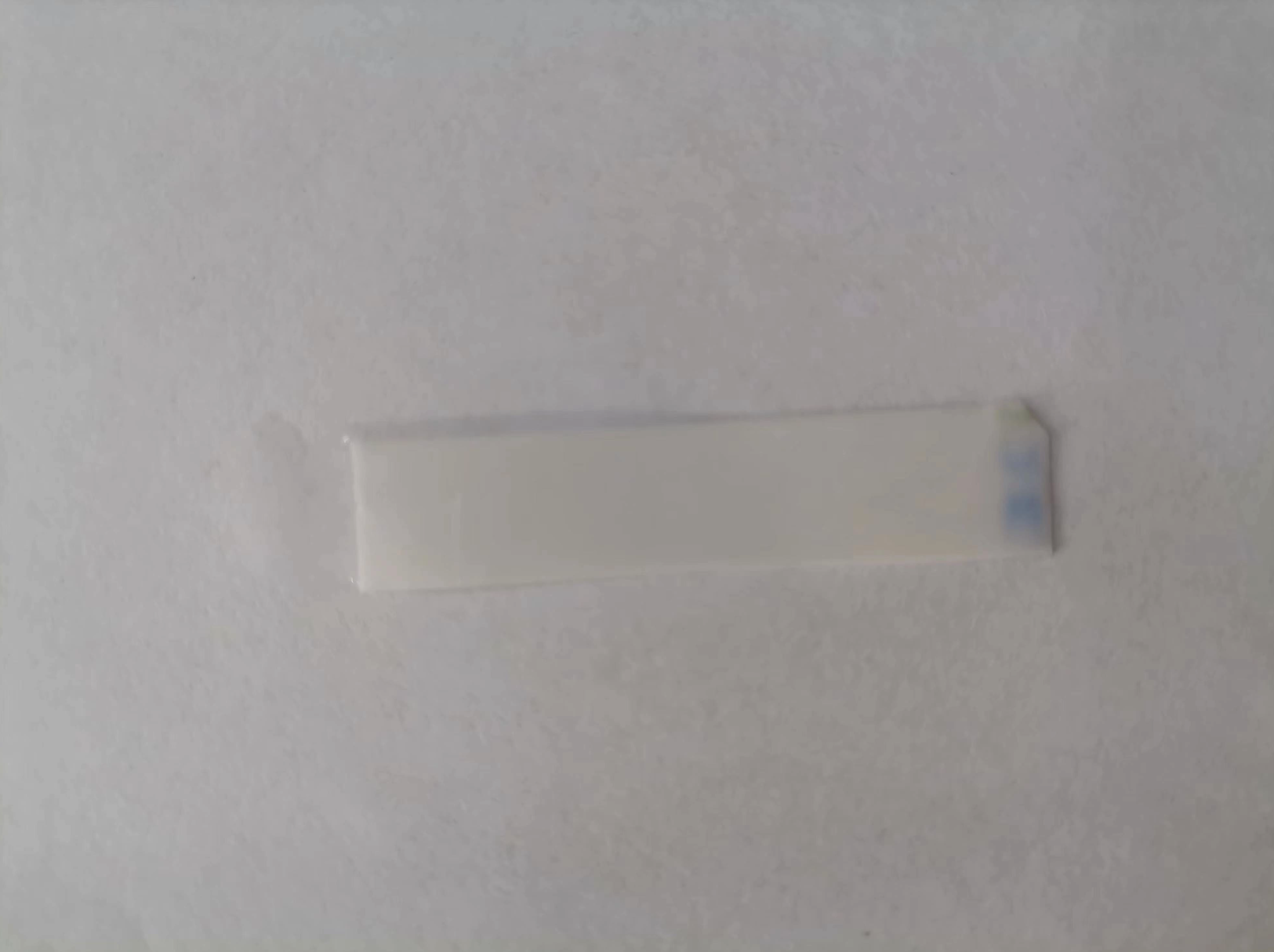

Supplement: Supplemental Information 5 — The protein extracts were separated and then electrotransferred to PVDF membranes. The PVDF membranes were cut at the level of the 36 and 52 kDa. The membranes were incubated with primary antibody and probed with HRP-coupled secondary antibody. Finally, the bands were visualized by using ECL reagents. [file peerj-11-15559-s005.zip › Raw GAPDH-02.tif]

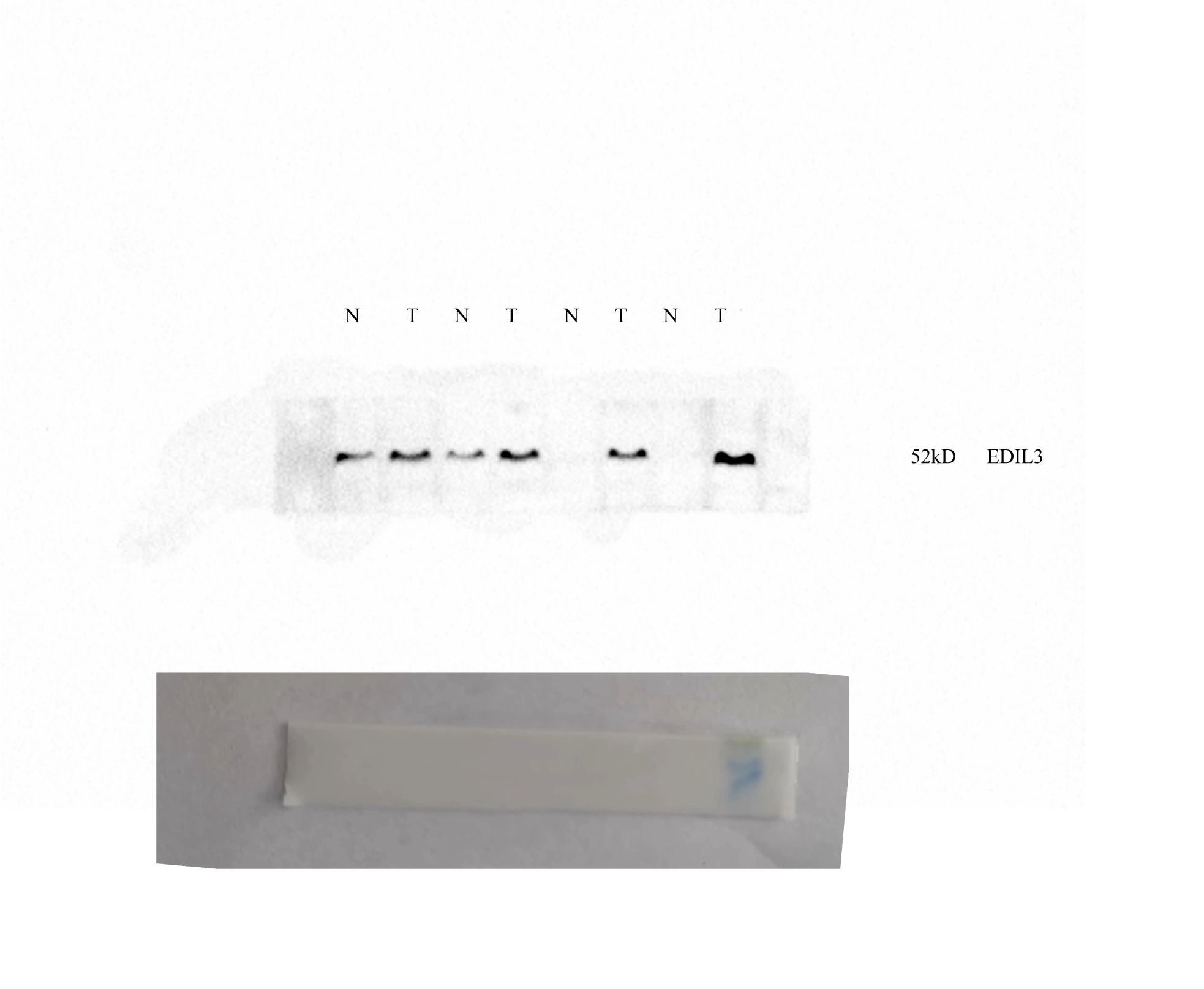

Supplement: Supplemental Information 5 — The protein extracts were separated and then electrotransferred to PVDF membranes. The PVDF membranes were cut at the level of the 36 and 52 kDa. The membranes were incubated with primary antibody and probed with HRP-coupled secondary antibody. Finally, the bands were visualized by using ECL reagents. [file peerj-11-15559-s005.zip › EDIL3.tif]
