# Supplementary material for: EDIL3 is a potential prognostic biomarker that correlates with immune infiltrates in gastric cancer
Source: PeerJ. 2023 Aug 9;11:e15559. doi: 10.7717/peerj.15559 (PMC10422953; doi:10.7717/peerj.15559)

## RealBand Pre-stained Protein Marker，3 colors，Wide Range，5~245 kDa

| Storage | -20°C |
| --- | --- |
| Categories | Protein Markers |

| Cat NO. | Package |
| --- | --- |
| [C610016-0001](http://www.life-biotech.com/product/productDetailForEN.html?productID=204296&sumProductID=204296) | 4 X 250 UL |


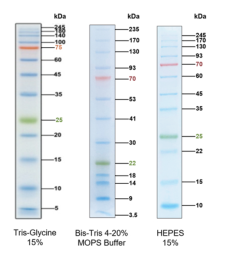

Supplement: Supplemental Information 5 — The protein extracts were separated and then electrotransferred to PVDF membranes. The PVDF membranes were cut at the level of the 36 and 52 kDa. The membranes were incubated with primary antibody and probed with HRP-coupled secondary antibody. Finally, the bands were visualized by using ECL reagents. [file peerj-11-15559-s005.zip › RealBand Protein Marker.docx]
